# Supplementary material for: Ultra-high-throughput mapping of the chemical space of asymmetric catalysis enables accelerated reaction discovery
Source: Nat Commun. 2023 Oct 21;14:6671. doi: 10.1038/s41467-023-42446-5 (PMC10590410; doi:10.1038/s41467-023-42446-5)
Supplement: Supplementary file 1 — Supplementary Information [file 41467_2023_42446_MOESM1_ESM.pdf]

Supplementary Information for

Ultra-High-Throughput Mapping of the Chemical Space of Asymmetric Catalysis Enables Accelerated Reaction Discovery

Wenjing Nie, Qiongqiong Wan, Jian Sun, Moran Chen, Ming Gao, Suming Chen*

Corresponding authors: sm.chen@whu.edu.cn

The PDF file includes:

Materials and Instrumentation
Supplementary Figure 1 to 11
Supplementary Table 1
Preparation of the compounds
Chiral HPLC data
NMR spectra
References

Table of contents

1. Materials...3

2. Instrumentation...3

3. Supplementary figures and table...4

Supplementary Fig. 1. Separation and quantification analysis of diastereomers **1a** by using IM-MS...4

Supplementary Fig. 2. The evaluation of the discrimination efficiency and qualification for the standard **2a** with five chiral resolving regents...5

Supplementary Fig. 3. Extracted ion mobilograms (EIMs) of underivatized and derivatized enantiomeric aldehyde and alcohols by chiral resolving reagent **D3**...7

Supplementary Fig. 4. Heatmap visualization of different *ee* values of **5b** analyzed by IM-MS...8

Supplementary Fig. 5. Reactions and corresponding compounds numbers of the direct asymmetric alkylation of aldehydes and Noyori asymmetric transfer hydrogenation...9

Supplementary Table 1 Enantiomers with different *ee* values analyzed by IM-MS method and chiral HPLC method...10

Supplementary Fig. 6. Photograph of the home-made setup for the high-throughput photochemical reaction, and the automated liquid handling system for liquid transfer...12

Supplementary Fig. 7. Chemical structures and numbers of the investigated 13 photocatalysts...13

Supplementary Fig. 8. Representative EIMs of the large-scale screening of direct asymmetric α-alkylation reactions with different organocatalysts and substrates...14

Supplementary Fig. 9. Investigation of the efficiency of CuAAC reaction and relative MS yields of the HTS 15

Supplementary Fig. 10. Optimization of reaction conditions with solvents, additive and forms of organocatalyst **A30**...16

Supplementary Fig. 11. The effect of the equivalence ratio of **A30** on the enantioselectivity of 96-well reactions ...16

4. Preparation of the compounds...17

5. Chiral HPLC data...29

6. NMR spectra...53

7. References...81

1. Materials

The chiral phosphoramidite diastereomeric standards were purchased form Sigma-Aldrich (USA), and enantiomeric standards Fmoc-propargyl-Gly-OH were purchased from Bide pharm company. All other commercially available regents for derivatization and synthesis were purchased form Bide pharm company (Shanghai, China) and Energy Chemical (Shanghai, China) with analytical grade. Dry solvents (water < 30 ppm) and deuterated solvents were purchased from J&K Scientific (Beijing, China). The chromatographic grade solvents (MeOH, ACN, and *i*-Pr-OH) for HPLC and IMS analysis were purchased form Thermo Fisher Scientific (USA). Silica gel (200-300 mesh) was used for column chromatography separation.

2. Instrumentation

Ion mobility (IM)-mass spectrometry (MS) analysis were performed on timsTOF Pro mass spectrometer (Bruker Daltonics, Germany) with a CaptiveSpray ion source. The final solutions were directly injected into the ion source by ultimate 3000 LC autosampler (Thermo Scientific, USA) with mobile phase. High-resolution (HR) MS data was acquired with Orbitrap Elite^{TM} mass spectrometer (Thermo Scientific, USA) with the following instrument parameters: FTMS positive mode, spray voltage: 3.8 kV, source heater temperature: 350 ^{o}C, sheath gas flow rate: 40, aux gas flow rate: 10. ^{1}H and ^{13}C NMR were recorded on Bruker Avance III NMR Spectrometer (600 M for ^{1}H NMR and 151 M for ^{13}C NMR). The chemical shift was referenced to corresponding residual solvent signals, such as data for ^{1}H NMR were recorded relative to $CHCl_3$ at δ 7.26 and ^{13}C NMR were recorded relative to $CHCl_3$ at δ 77.15. Abbreviations for multiplicity: s = singlet, d = doublet, t = triplet, q = quartet, m = multiplet, dd = doublet of doublet, dt = doublet of triplet, td = triplet of double. The enantiomeric excess (*ee*) values were determined by Agilent 1260 infinity with UV detector, and Chiralcel OD-H column and IC column were used for separation of synthesized enantiomers.

3. Supplementary Figures and Table

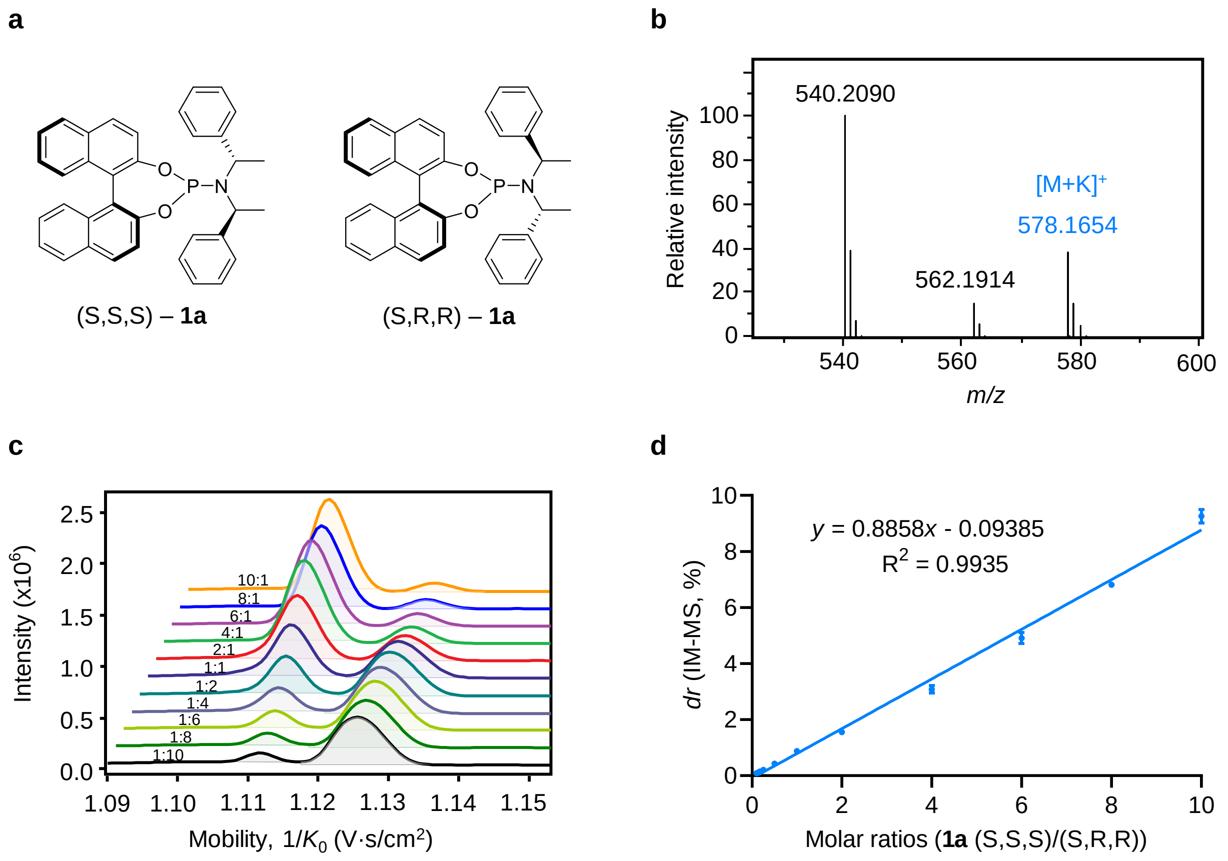

Supplementary Fig. 1. Separation and quantification analysis of diastereomers 1a by using IM-MS. (**a**) Structures of the chiral phosphoramidite **1a** diastereomers. (**b**) Mass spectrum of **1a** with different ion adducts, which potassium ion adduct shows more promising resolving power. Peaks at *m/z* 540.2090 and 562.1914 denote the protonated and sodiated ions of **1a**, respectively. (**c**) Stack of the extracted ion mobilograms (EIMs) of diastereomers **1a** in different ratios of (*S*,*S*,*S*)-configuration and (*S*,*R*,*R*)-configuration. (**d**) Liner relationship between diastereomers **1a** molar ratios and diastereomeric ratio (*dr*) determined by IM-MS, data are presented as mean values ± SD, $n = 3$ independent replicates.

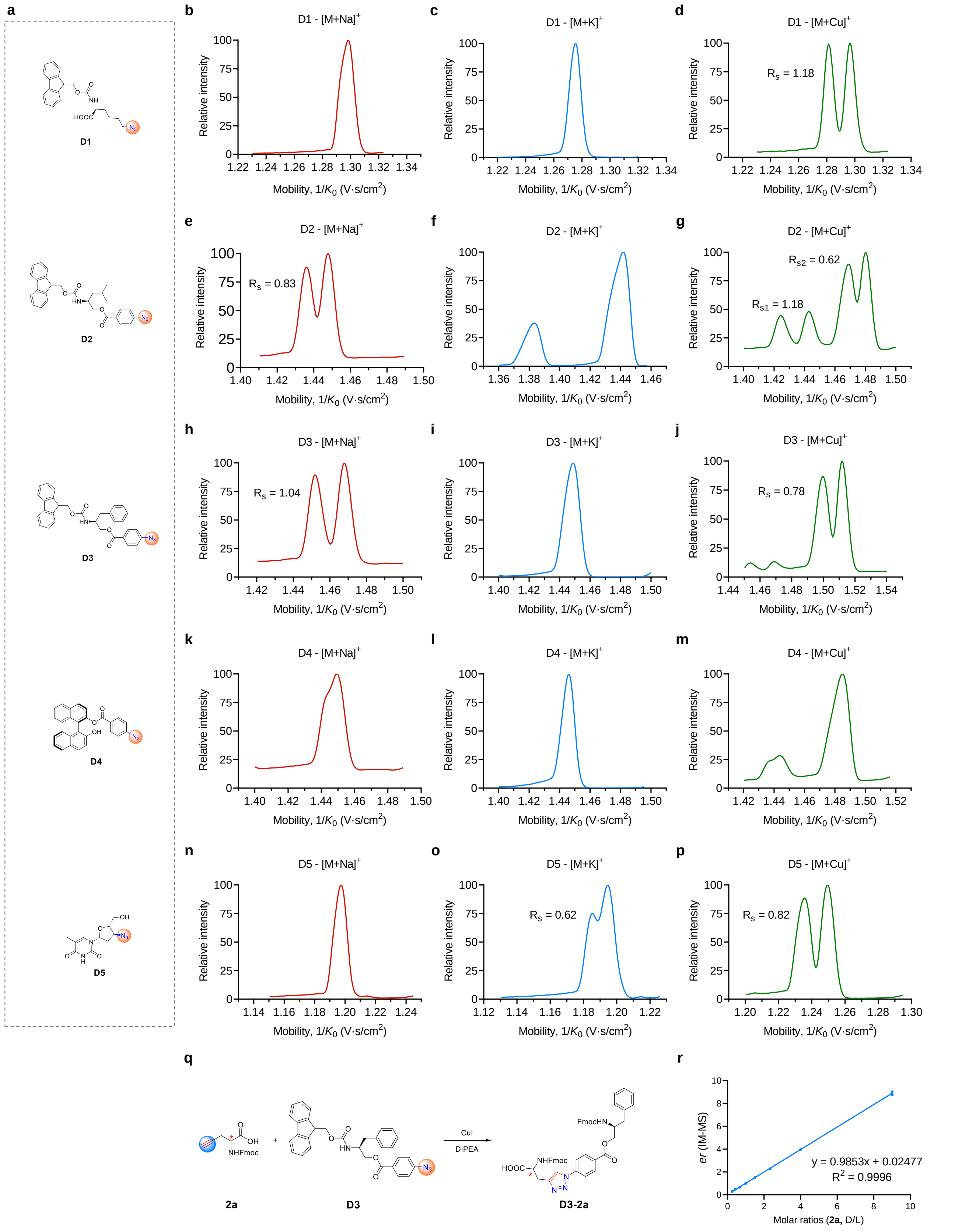

Supplementary Fig. 2. The evaluation of the discrimination efficiency and qualification for the standard 2a with five chiral resolving regents. (**a**) Structures of **D1-D5** chiral resolving reagents. (**b**)-(**d**) showing the extracted ion mobilograms (EIMs) of formed diastereomers **D1-2a** with sodium, potassium, and copper (I) adduct ions, and R_s

= 1.18 for copper (I) adduct ions. (**e**)-(**g**) EIMs for **D2-2a**, and R_s = 0.83 for sodium adduct ions and there are two complexation sites for copper (I) which R_{s1} = 1.18, R_{s2} = 0.62. (**h**)-(**j**) EIMs for **D3-2a**, and R_s = 1.04 for sodium adduct ion R_s = 0.78 for copper (I) adduct ions. (**k**)-(**m**) EIMs for **D4-2a**. (**n**)-(**p**) EIMs for **D5-2a**, and R_s = 0.62 for potassium adduct ions, R_s = 0.82 for copper (I) adduct ions. (**q**) Derivatization reaction for **2a** with **D3** chiral resolving reagent. (**r**) Qualification power for enantiomer **2a** with **D3** chiral resolving reagent by post-derivatization strategy, data are presented as mean values ± SD, n = 3 independent replicates. The $[M + Cu]^+$ showed higher peak resolution with R_s = 1.18 of diastereomer derivatized by **D1**, but the copper (I) adduct ions of diastereomers derivatized by **D2** and **D3** demonstrated that there were two complexation sites which made the IMS analysis more difficult when applied the strategy to other analysts. R_s represents the measured mobilograms of formed diastereomers and is defined as $R_S = 2.35 \times \frac{A_2 - A_1}{2 \times (W_{FWHM1} + W_{FWHM2})}$, where A_1 and A_2 represent the $1/K_0$ value of formed diastereomers with observed adduct ion and W_{FWHM1} and W_{FWHM2} represent the full peak width at half-maximum of mobilogram of formed diastereomers.

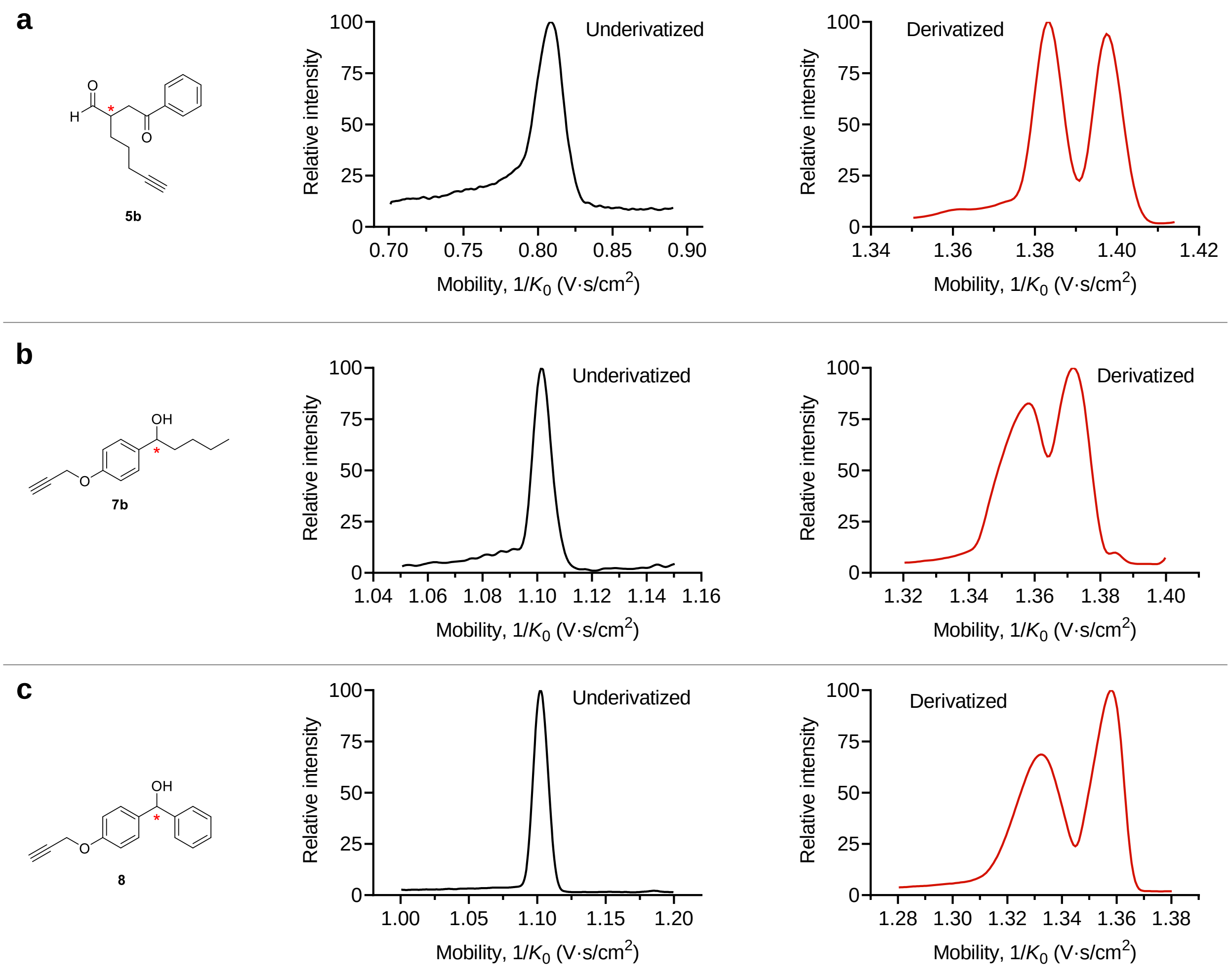

Supplementary Fig. 3. Extracted ion mobilograms (EIMs) of underivatized and derivatized racemic aldehyde and alcohols by chiral resolving reagent D3. (**a**)-(**c**) Ion mobility MS analysis of racemic (**a**) aldehyde **5b**, (**b**) alcohols **7b**, and (**c**) **8** before and after derivatization.

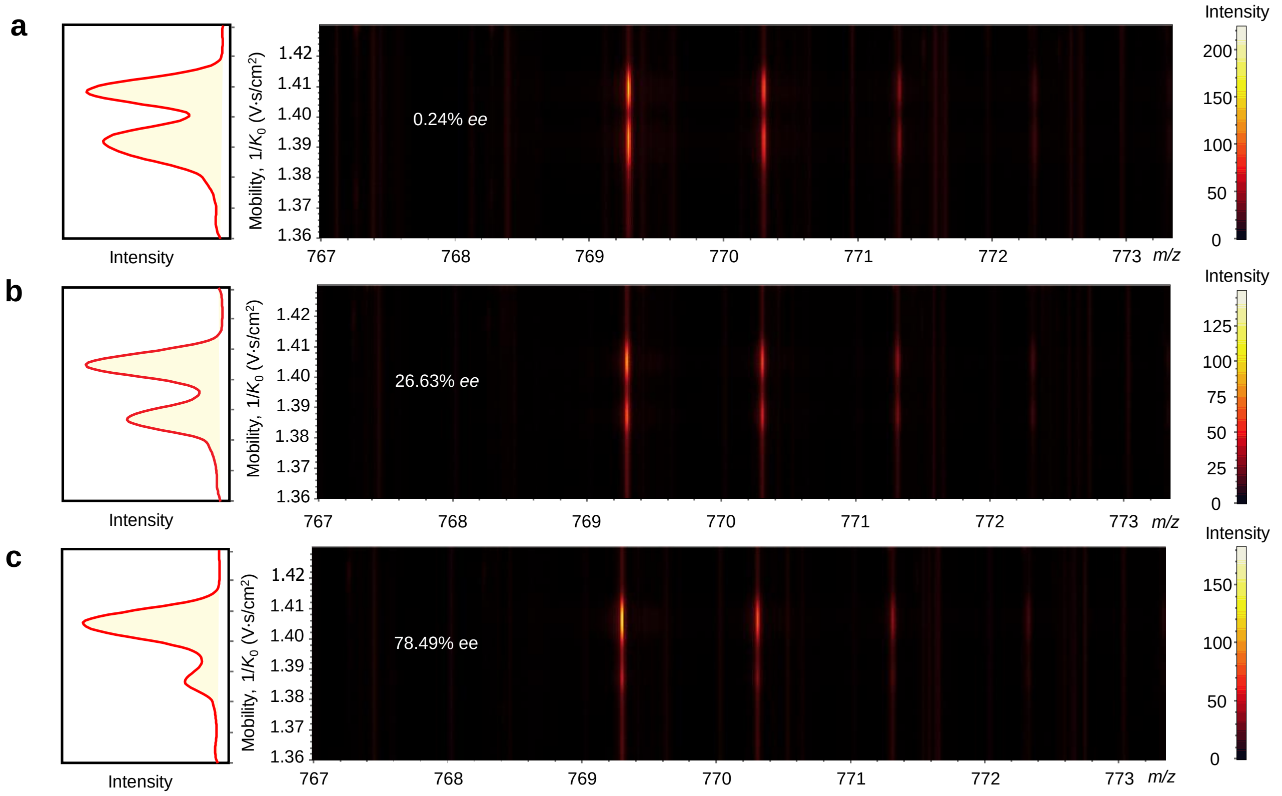

Supplementary Fig. 4. Heatmap visualization of different *ee* values of 5b analyzed by IM-MS. (**a**) Mobilogram and corresponding heatmap of **5b** with 0.24% *ee*; (**b**) Mobilogram and corresponding heatmap of **5b** with 26.63% *ee*; (**c**) Mobilogram and corresponding heatmap of **5b** with 78.49% *ee*.

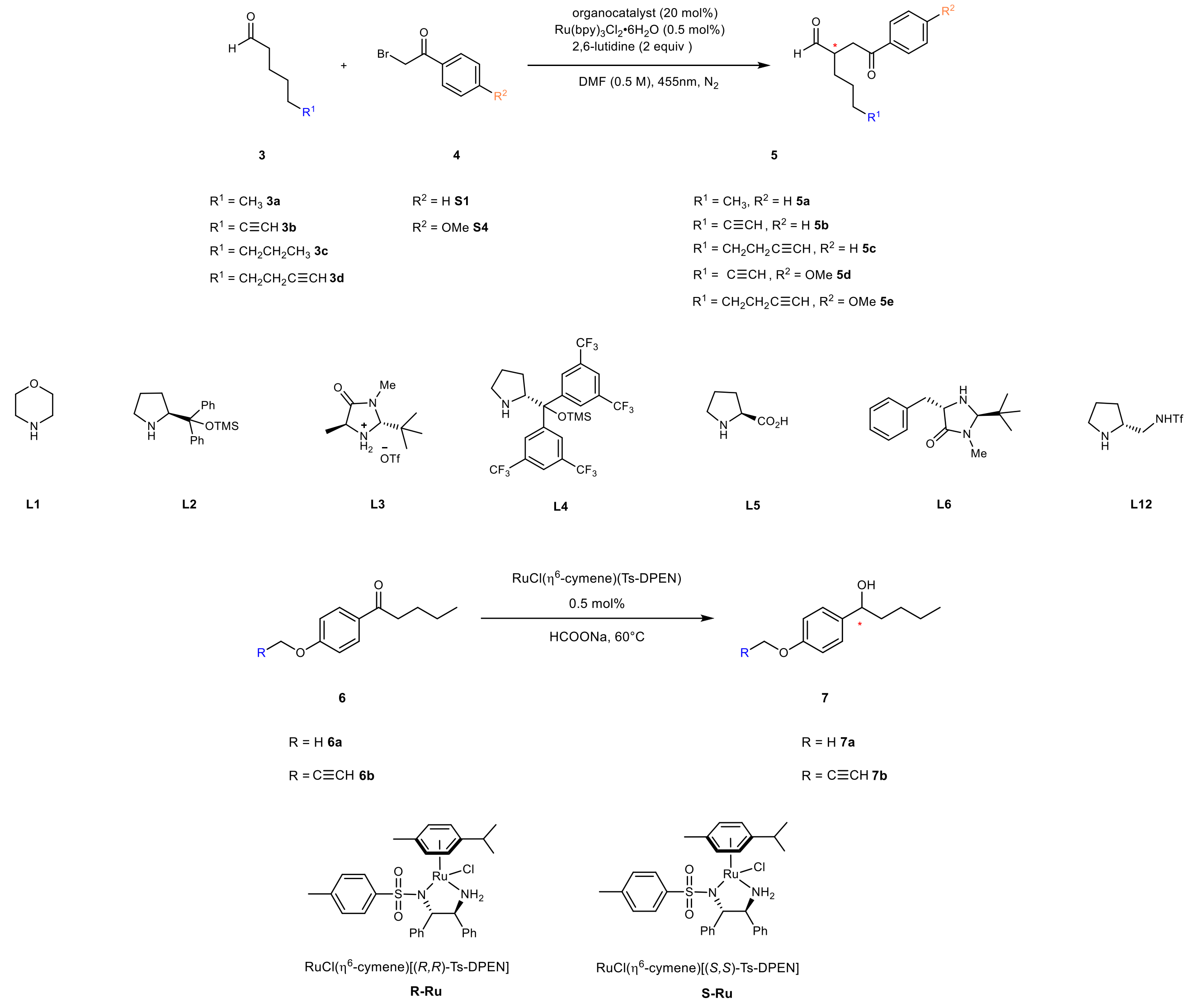

Supplementary Fig. 5. Reactions and corresponding compounds numbers of the direct asymmetric alkylation of aldehydes and Noyori asymmetric transfer hydrogenation.

Supplementary Table 1. Enantiomers (41) with different *ee* values analyzed by IM-MS method and chiral HPLC method.

enantiomers	condition	observed *m/z*	$1/K_0$ difference ($V\cdot s/cm^2$)	△ CCS($Å^2$)	R_s	*ee* (IM-MS, %)	*ee* (HPLC, %)
	D/L ratio						
2a	2:8	876.2990	0.018	3.7	0.78	-55.42	-60.00
2a	3:7	876.2991	0.017	3.5	0.77	-34.48	-40.00
2a	4:6	876.2991	0.017	3.5	0.77	-19.96	-20.00
2a	5:5	876.2993	0.017	3.5	0.80	-0.39	0.00
2a	6:4	876.2994	0.016	3.3	0.72	20.39	20.00
2a	7:3	876.2993	0.016	3.3	0.72	39.07	40.00
2a	8:2	876.2992	0.016	3.3	0.78	59.72	60.00
2a	9:1	876.2994	0.015	3.1	0.77	79.80	80.00
	Condition A						
L1-5b	L1	769.2980	0.017	3.5	0.82	0.82	-0.22
L3-5b	L3	769.2981	0.020	4.1	1.12	70.49	69.75
L4-5b	L4	769.2979	0.013	2.6	1.02	-52.26	-48.45
L5-5b	L5	769.2977	0.018	3.6	1.06	-7.31	-6.74
L12-5b	L13	769.2974	0.017	3.4	0.95	2.54	5.48
L1-5d	L1	839.2482	0.008	1.6	0.55	-3.48	-1.09
L3-5d-1	L3	839.2482	0.008	1.4	0.55	65.88	68.40
L3-5d	L3	839.2484	0.009	1.8	0.62	66.16	67.58
L4-5d	L4	839.2485	0.004	0.8	0.39	-59.02	-58.23
L5-5d	L5	839.2484	0.008	1.6	0.55	-4.23	-6.99
L6-5d	L6	839.2486	0.013	2.7	0.76	43.86	45.19
L12-5d	L13	839.2484	0.009	1.8	0.62	17.86	16.96
L1-5c	L1	713.2365	0.015	3.0	0.70	2.23	-0.65
L1-5e	L1	743.2472	0.011	2.2	0.68	5.26	-0.10
L2-5e	L2	743.2468	0.015	3.1	0.84	38.34	41.21
L1-5b-1	L1	769.2998	0.016	3.2	0.85	-0.43	0.24
L2-5b-1	L2	769.2996	0.020	3.4	1.31	9.86	9.58
L2-5b-2	L2	769.2998	0.017	3.4	1.18	12.45	13.17
L2-5b-3	L2	769.2999	0.017	3.4	1.18	23.41	23.49
	Condition B						
L2-5b-4	L2	769.2997	0.018	3.6	1.18	26.26	27.99
L2-5b-5	L2	769.2997	0.018	3.6	1.24	28.23	31.03
L2-5b-6	L2	769.2996	0.018	3.6	1.24	27.63	26.63
L3-5b-1	L3	769.2997	0.020	3.9	1.12	78.11	84.86
L3-5b-2	L3	769.2996	0.023	4.7	1.13	77.29	83.78
L3-5b-3	L3	769.2996	0.019	3.8	1.17	74.80	78.49
	Condition C						
$NaBH_4$-7b	$NaBH_4$	759.3148	0.014	2.9	0.57	-3.94	0.48

$NaBH_4$-7b-1	$NaBH_4$	759.3153	0.013	2.7	0.55	0.21	0.83
R-Ru-7b-1	R-Ru	759.3152	0.008	1.7	0.72	75.91	77.83
R-Ru-7b-2	R-Ru	759.3151	0.009	1.8	0.76	74.06	71.65
R-Ru-7b-3	R-Ru	759.3151	0.008	1.6	0.49	76.00	76.83
S-Ru-7b-1	S-Ru	759.3153	0.011	2.2	0.86	-77.20	-82.55
S-Ru-7b-2	S-Ru	759.3154	0.011	2.9	0.65	-74.16	-73.70
S-Ru-7b-3	S-Ru	759.3155	0.016	3.3	0.90	-75.67	-73.34

Note 1: condition A: The reactions were operated in a 5 W 455 nm blue LED photoreactor with different organocatalysts; condition B: The reactions were operated in a 5 W 455 nm blue LED photoreactor connected a water-cooling circulation system with different organocatalysts; condition C: The reactions were operated with $NaBH_4$ and different configuration Ru catalyst. Note 2: As for glycine derivative **2a**, the definition of *ee*: $ee(HPLC,\ \%) = \frac{C_D \times V_D - C_L \times V_L}{C_D \times V_D + C_L \times V_L} \times 100$, the corresponding calculation of *ee* determined by IMMS is defined as follow: $ee(IM - MS, \%) = \frac{M_D - M_L}{M_D + M_L} \times 100$; as for the calculation of chiral HPLC analysis, the definition of *ee*: $ee(HPLC,\ \%) = \frac{A_R - A_S}{A_R + A_S} \times 100$, the corresponding calculation of *ee* determined by IM-MS is defined as follow: $ee(IM - MS, \%) = \frac{M_R - M_S}{M_R + M_S} \times 100$. C_D and C_L refer to the origin concentration of **D-2a** and **L-2a**, V_D and V_L refer to the volume of **D-2a** and **L-2a**. M_R and M_S (M_D and M_L) refer to the mobilogram peak area the of corresponding diastereomers. A_R and A_S refer to the integral area of *R* and *S*-configuration reaction product with chiral HPLC analysis.

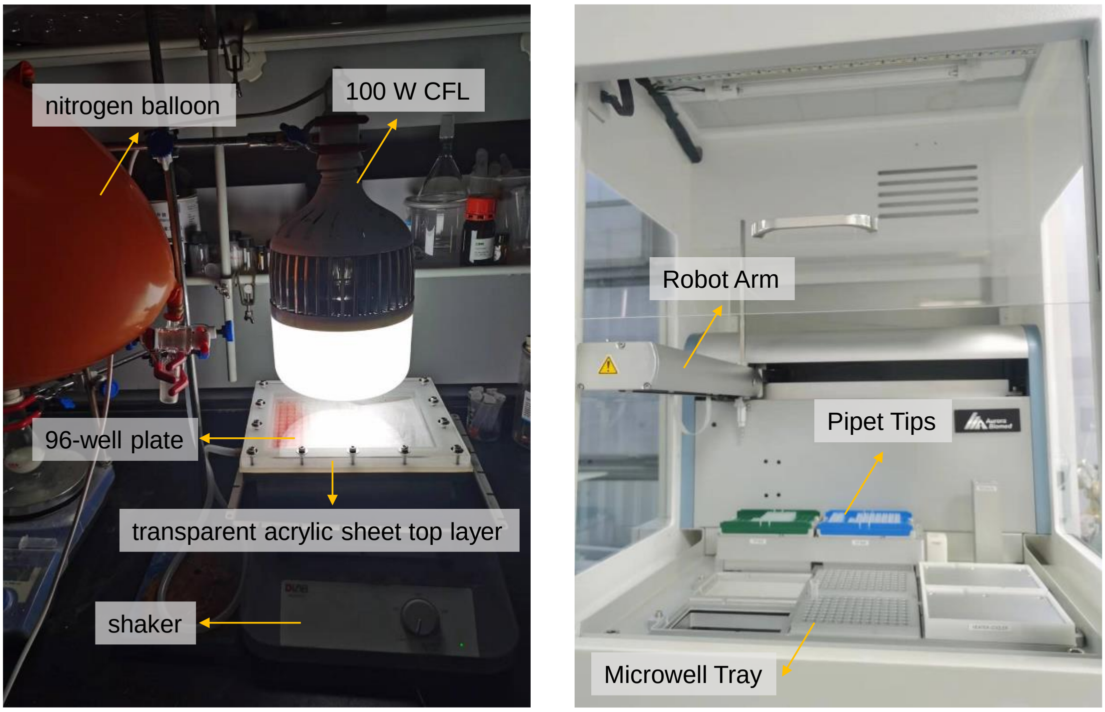

Supplementary Fig. 6. Photograph of the home-made setup for the high-throughput photochemical reaction, and the automated liquid handling system for liquid transfer (VERSA 110, Aurora Biomed, Canada).

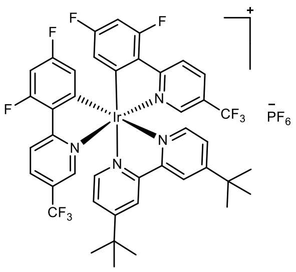

$[Ir(2',4'\text{-}dF\text{-}5\text{-}CF_3\text{-}ppy)_2(4,4'\text{-}dtb\text{-}bpy)]PF_6$

P1

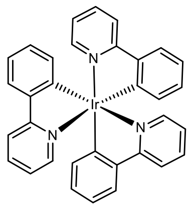

fac-Ir(ppy)$_3$

P2

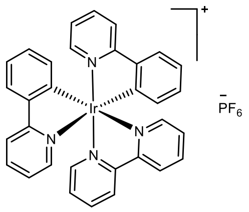

$[Ir(ppy)_2(bpy)]PF_6$

P3

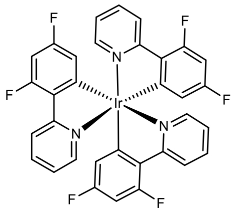

fac-Ir(2',4'-dF-ppy)$_3$

P4

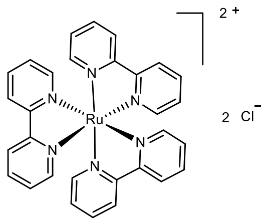

$[Ru(bpy)_3]Cl_2{\bullet}6H_2O$

P5

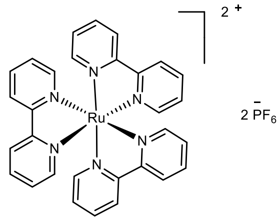

$[Ru(bpy)_3](PF_6)_2$

P6

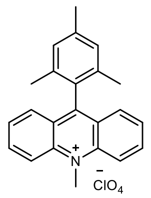

Mes-Acr-ClO$_4$

P7

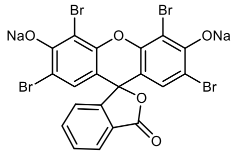

Na$_2$- Eosin Y

P8

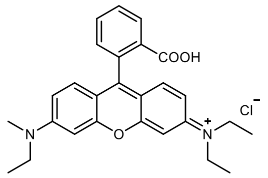

Rhodamine B

P9

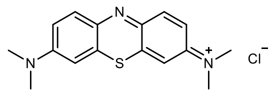

Methylene blue

P10

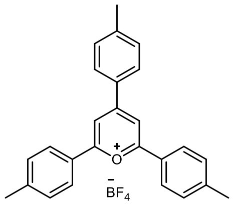

p-Me TPT

P11

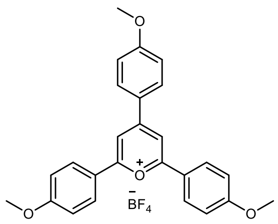

p-OMe TPT

P12

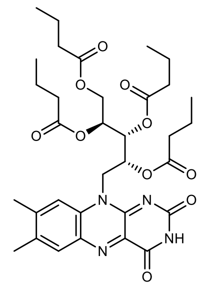

Riboflavin tetrabutyrate

P13

Supplementary Fig. 7. Chemical structures and numbers of the investigated 13 photocatalysts.

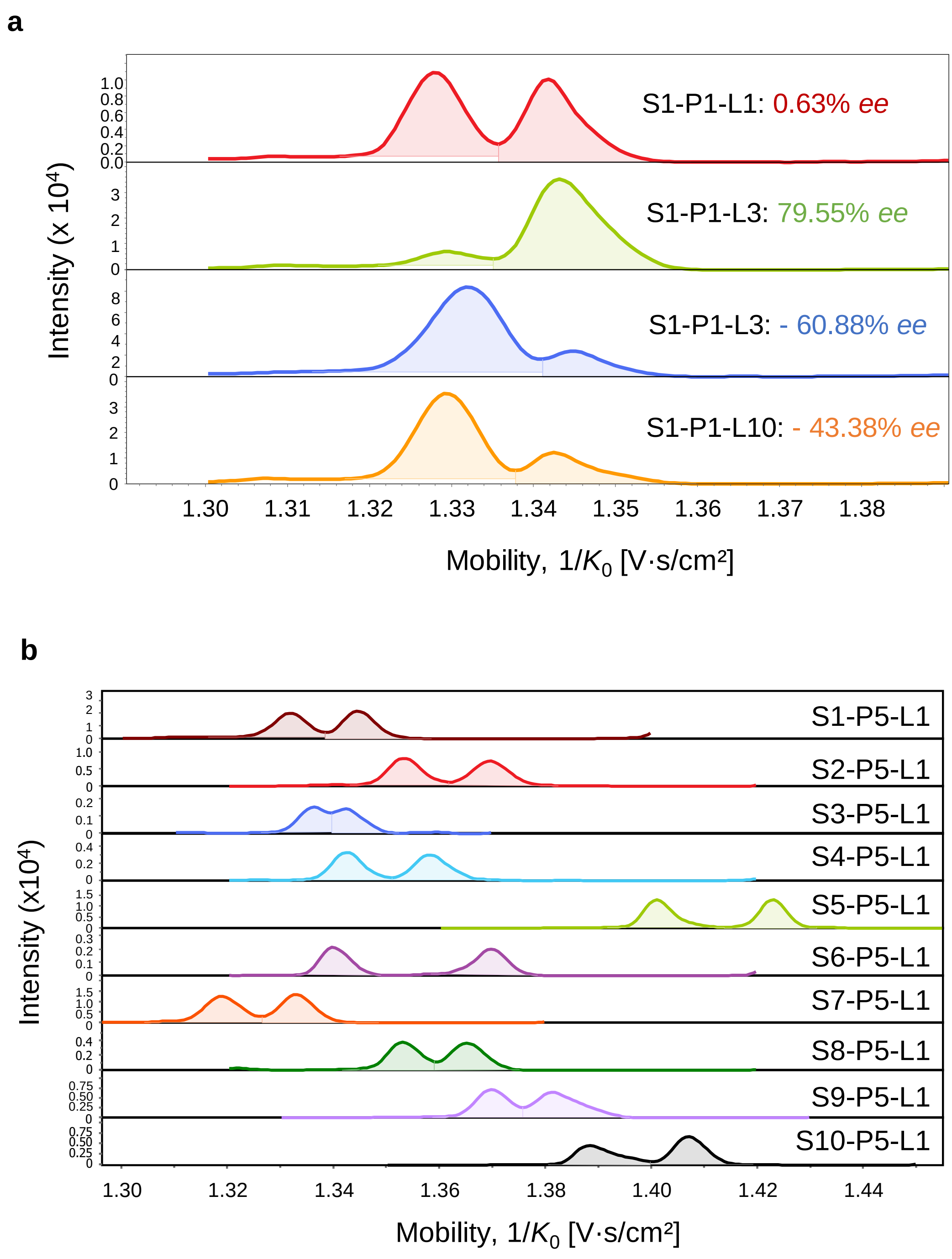

Supplementary Fig. 8. Representative EIMs of the large-scale screening of direct asymmetric α-alkylation reactions with different organocatalysts and substrates. (**a**) EIMs of reactions of substrate **S1** catalyzed by **P1** and different organocatalysts; (**b**) EIMs of reactions of different substrates catalyzed by **P5** and **L1**.

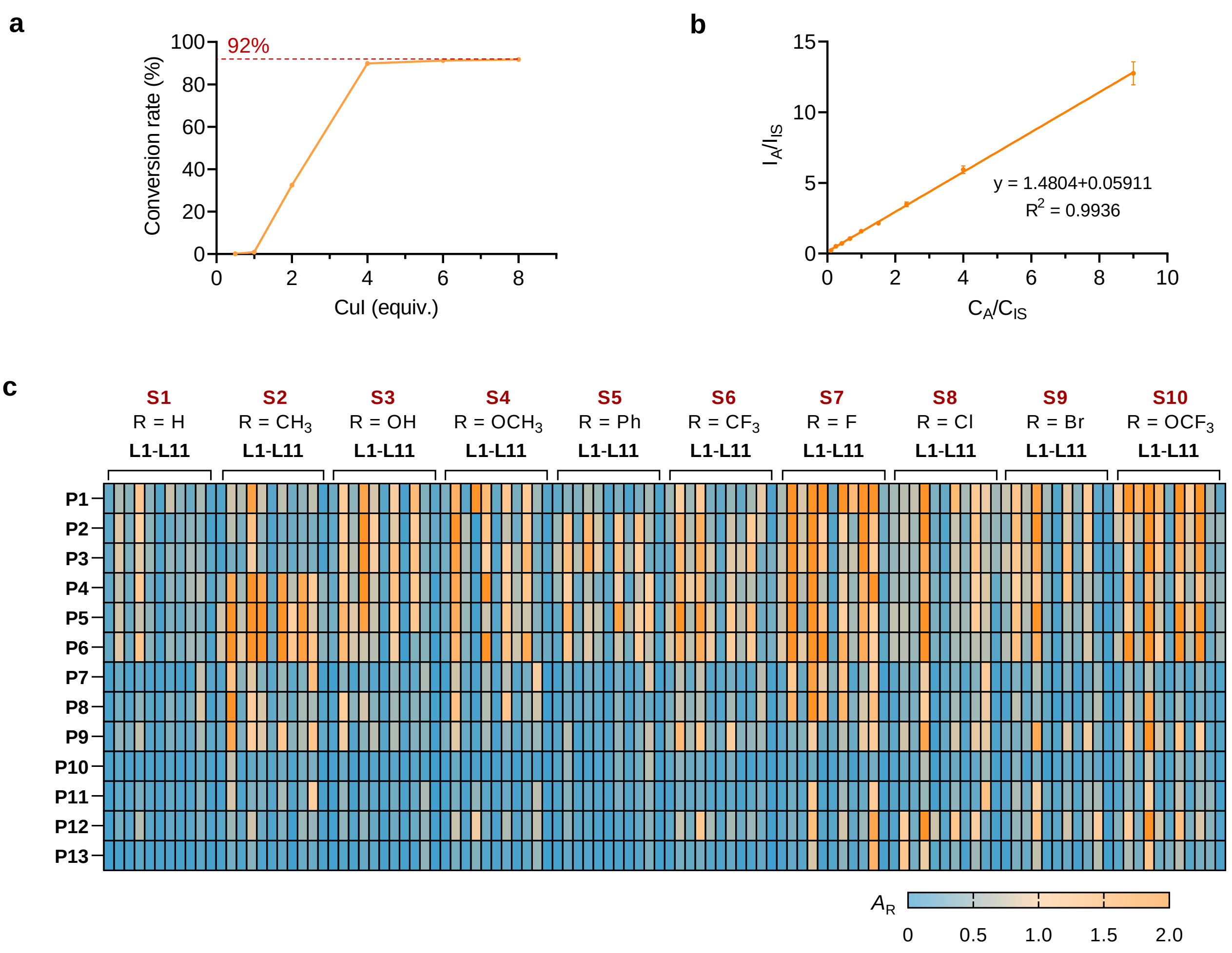

Supplementary Fig. 9. Investigation of the efficiency of CuAAC reaction and relative MS yields of the HTS. **(a)** Investigation of the efficiency of CuAAC reaction by optimizing the equivalent of CuI with **D3** and **5d**. **(b)** Linear relationship between internal standard **4** (1-phenylprop-2-yn-1-ol) and substrate **S1**. **(c)** Heatmap visualization of the relative MS yields of the 1430 reactions by calculating the relative peak areas of ions ($A_R = A_{product}/A_{internal\ standard}$) between the derivatives from the reaction products and internal standard **4**. Data are presented as mean values ± SD, n = 3 independent replicates.

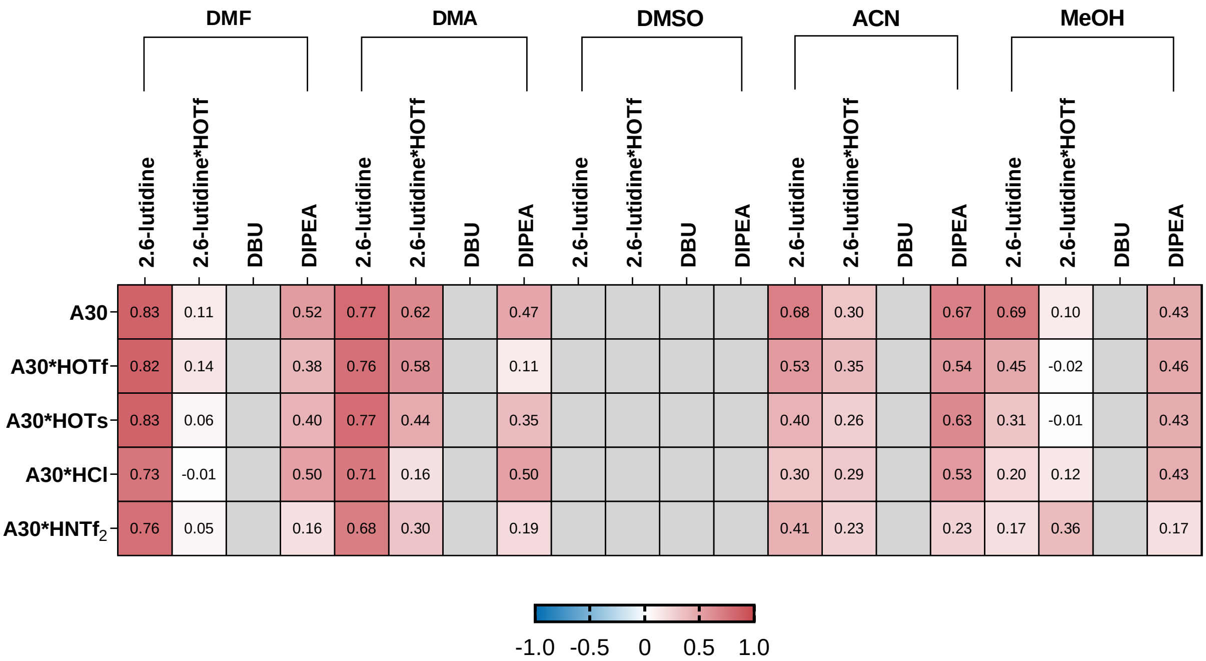

Supplementary Fig. 10. Optimization of reaction conditions with solvents (5), additive (4) and forms of organocatalyst A30 (5). The screening results indicated that the solvent of DMSO and the additive of DBU were unfavorable for the reaction, while amide solvents like DMF and DMA were compatible with the reaction. HOTf, trifluoromethanesulfonic acid; HOTs, 4-methylbenzenesulfonic acid; $HNTf_2$, 1,1,1-trifluoro-*N*-((trifluoromethyl)sulfonyl)methanesulfonamide; DBU, 1,8-Diazabicyclo[5,4,0]undec-7-ene; DIPEA, *N*,*N*-Diisopropylethylamine. Gray boxes indicate that the product was not acquired and detected by IM-MS.

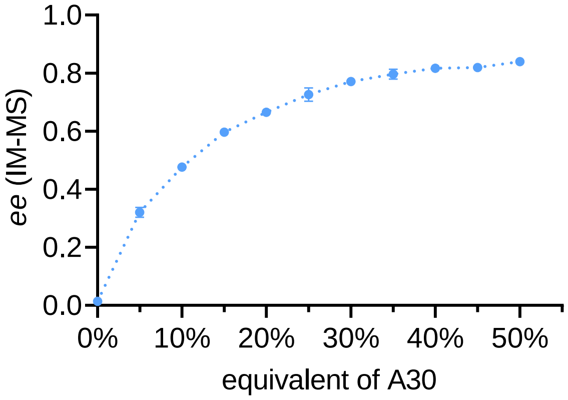

Supplementary Fig. 11. Optimization of the equivalent of organocatalyst A30. The enantioselectivity was gradually improved with the increase of the equivalent of **A30**, and the equivalent of **A30** was fixed as 30% for the optimization of reaction conditions. Data are presented as mean values ± SD, $n = 3$ independent replicates.

5. Preparation of the compounds

5.1 Synthesis of chemical derivatization reagents:

5.1.1 Synthesis of (*S*)-2-(((9*H*-fluoren-9-yl)methoxy)carbonyl)amino)-4-methylpentyl 4-azidobenzoate (D2) [1], [2]:

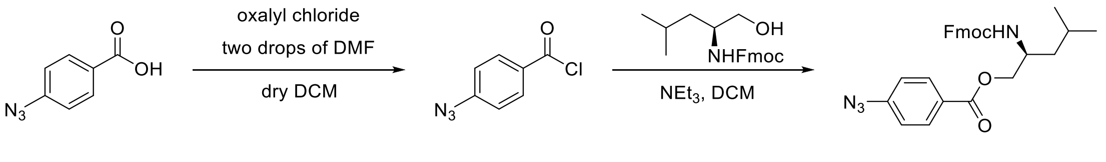

D2

To a 50 mL single-necked round-bottom flask equipped with magnetic stir bar 4-azidobenzoic acid (816 mg, 5 mmol) and 20 mL dry dichloromethane (DCM) were added. Then two drops of *N,N*-dimethylformamide (DMF) were added to the solution, followed by oxalyl chloride (850 μL, 2 equiv) was added portion-wise to the reaction mixture. After 30 min, the reaction mixture was concentrated by rotary evaporation to remove the solvent and excess oxalyl chloride to get crude 4-azidobenzoyl chloride as white solid. (9*H*-fluoren-9-yl) methyl (*S*)-(1-hydroxy-4-methylpentan-2-yl) carbamate (1.7 g, 1 equiv) and triethylamine (700 μL,1 equiv) were dissolved with 20 mL dry DCM and the solution was drop-wise added to a 50 mL single-necked flask contained 4-azidobenzoyl chloride crude product. After stirring overnight, the reaction mixture was concentrated by rotary evaporation to remove solvent and triethylamine. The crude product was purified with petroleum ether: ethyl acetate (5:1, v/v) to obtain the desire product **D2** as pale-yellow solid (1.16 g, 48%). ^{1}H NMR (600 MHz, $CDCl_3$) δ 7.99 (d, *J* = 8.3 Hz, 2H), 7.74 (d, *J* = 7.6 Hz, 2H), 7.61–7.48 (m, 2H), 7.38 (t, *J* = 7.5 Hz, 2H), 7.31–7.26 (m, 2H), 7.00 (d, *J* = 8.3 Hz, 2H), 4.73 (d, *J* = 8.9 Hz, 1H), 4.47–4.36 (m, 2H), 4.35–4.24 (m, 2H), 4.15 (t, *J* = 6.0 Hz, 2H), 1.80–1.58 (m, 2H), 1.52–1.32 (m, 2H), 0.96 (dd, *J* = 6.6, 4.1 Hz, 6H); ^{13}C NMR (151 MHz, $CDCl_3$) δ165.83, 156.15, 145.08, 143.95, 141.44, 131.64, 127.81, 127.17, 126.48, 125.11, 120.10, 118.99, 67.24, 66.71, 48.84, 47.43, 40.98, 24.87, 23.22, 22.24; HRMS (ESI+) exact mass calculated for ($C_{28}H_{28}N_4O_4$) $[M+H]^+$ requires *m/z* 485.2183, found *m/z* 485.2168.

5.1.2 Synthesis of (*S*)-2-(((9*H*-fluoren-9-yl)methoxy)carbonyl)amino)-3-phenylpropyl 4-azidobenzoate (D3) [1], [2]:

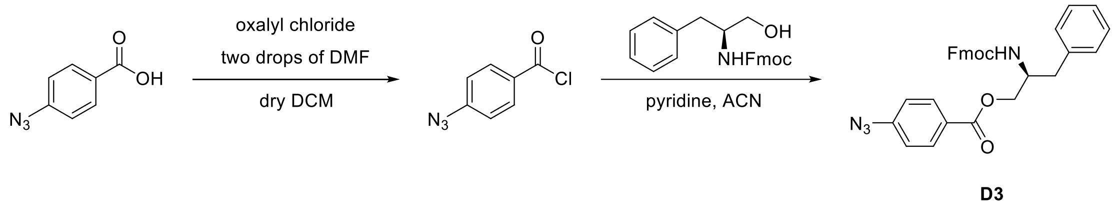

D3

To a 50 mL single-necked round-bottom flask equipped with magnetic stir bar 4-azidobenzoic acid (816 mg, 5 mmol) and 20 mL dry dichloromethane were added. Then two drops of DMF were added to the solution, followed by oxalyl chloride (850 μL, 2 equiv) drop-wisely added to the reaction mixture. After 30 min, the reaction mixture was concentrated by rotary evaporation to remove the solvent and excess oxalyl chloride to get 4-azidobenzoyl chloride crude product as white solid. Without further purification, the crude 4-azidobenzoyl chloride was dissolved with 20 mL acetonitrile (ACN). The solution was drop-wise added to a 50 mL single-necked flask contained Fmoc-Protected L-phenylalanine alcohol (1.87 g, 1 equiv) and pyridine (403 μL, 1 equiv). After stirring overnight, the reaction mixture turned into a white suspension and concentrated to remove ACN. The crude product was washed by ethanol (20 mL × 3) and dried in oven to get the desire product **D3** as white solid (1.94 g, 75%). ^{1}H NMR (600 MHz, $CDCl_3$)

δ 8.01 (d, J = 8.3 Hz, 2H), 7.75 (dd, J = 7.6, 4.0 Hz, 2H), 7.52 (d, J = 7.5 Hz, 2H), 7.39 (t, J = 7.5 Hz, 2H), 7.35–7.16 (m, 7H), 7.04 (d, J = 8.3 Hz, 2H), 4.95 (d, J = 8.2 Hz, 1H), 4.42–4.26 (m, 4H), 4.17 (t, J = 6.9 Hz, 1H), 2.90–3.15 (m, 2H); ^{13}C NMR (151 MHz, $CDCl_3$) δ 165.76, 155.91, 145.21, 143.92, 141.40, 136.83, 131.65, 129.41, 128.88, 127.83, 127.15, 127.04, 126.27, 125.14, 120.11, 119.04, 66.85, 65.58, 51.53, 47.30, 38.05; HRMS (ESI+) exact mass calculated for ($C_{31}H_{26}N_4O_4$) $[M+H]^+$ requires *m/z* 519.2027, found *m/z* 519.2013.

5.1.3 Synthesis of (*S*)-2'-hydroxy-[1,1'-binaphthalen]-2-yl 4-azidobenzoate (D4) [1], [2]:

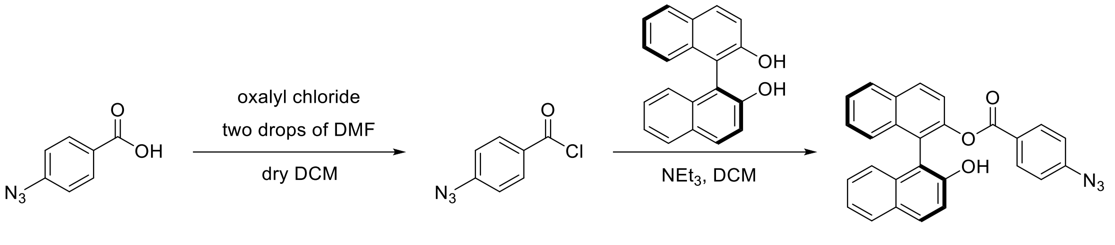

D4

To a 50 mL single-necked round-bottom flask equipped with magnetic stir bar 4-azidobenzoic acid (816 mg, 5 mmol) and 20 mL dry dichloromethane were added. Then two drops of DMF were added to the solution, followed by oxalyl chloride (850 μL, 2 equiv) slowly added to the reaction mixture. After 30 min, the reaction mixture was concentrated by rotary evaporation to remove the solvent and excess oxalyl chloride to get crude 4-azidobenzoyl chloride as white solid. (*S*)-(-)-1, 1'-bi-2-naphthol (1.43 g, 1 equiv) and triethylamine (700 μL,1 equiv) were dissolved with 20 mL dry DCM and the solution was drop-wise added to a 50 mL single-necked flask contained 4-azidobenzoyl chloride crude product. After stirring overnight, the reaction mixture was concentrated by rotary evaporation to remove solvent and triethylamine. The crude product was purified by recrystallization with petroleum ether: ethyl acetate (5:1, v/v) to obtain the desire product **D4** as white crystalline solid (2.1 g, 87%). ^{1}H NMR (600 MHz, $CDCl_3$) δ 8.04 (d, J = 8.9 Hz, 1H), 7.92 (d, J = 8.2 Hz, 1H), 7.76–7.67 (m, 2H), 7.54 (d, J = 8.6 Hz, 2H), 7.49–7.41 (m, 2H), 7.31–7.15 (m, 5H), 7.07 (dd, J = 8.2, 1.4 Hz, 1H), 6.77 (d, J = 8.6 Hz, 2H), 5.21 (s, 1H); ^{13}C NMR (151 MHz, $CDCl_3$) δ 165.13, 151.83, 148.34, 145.48, 133.61, 133.59, 132.42, 131.87, 130.99, 130.52, 129.08, 128.51, 128.15, 127.69, 126.84, 126.51, 125.89, 125.23, 124.68, 123.61, 123.19, 121.93, 118.87, 118.24, 113.97; HRMS (ESI+) exact mass calculated for ($C_{27}H_{17}N_3O_3$) $[M+H]^+$ requires *m/z* 432.1343, found *m/z* 432.1337.

5.2 Noyori asymmetric transfer hydrogenation

5.2.1 Synthesis of 1-(4-methoxyphenyl) pentan-1-one (6a) [3]:

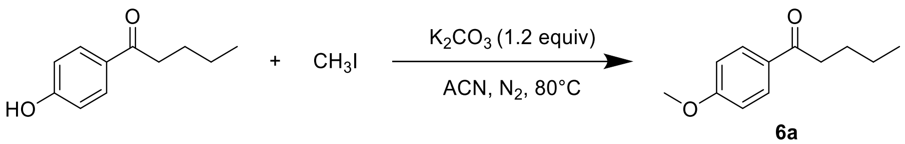

6a

To a 50 mL double-necked round-bottom flask equipped with a reflux condenser and magnetic stir bar 1-(4-hydroxyphenyl) pentan-1-one (892 mg, 5 mmol) and potassium carbonate (830 mg, 1.2 equiv) were added. 20 mL of ACN was added to the flask via syringe, followed by iodomethane (852 mg, 1.2 equiv) under nitrogen atmosphere. The reaction mixture was heated to 80 °C and reflux overnight. Upon monitored by thin layer chromatography (TLC), the reaction mixture was filtered to remove the potassium carbonate and the filtrate was concentrated by rotary evaporation. The crude product was purified by silica gel chromatography using hexane/ethyl acetate (10:1, v/v) as eluent to obtain 820 mg (85%) of **6a** product as white crystal solid. ^{1}H NMR (600 MHz, $CDCl_3$) δ 7.94 (d, J = 8.7

Hz, 2H), 6.92 (d, J = 8.8 Hz, 2H), 3.86 (s, 3H), 3.06–2.81 (m, 2H), 1.75–1.66 (m, 2H), 1.36–1.43 (m, 2H), 0.94 (t, J = 7.4 Hz, 3H); ^{13}C NMR (151 MHz, $CDCl_3$) δ 199.40, 163.39, 130.43, 130.25, 113.75, 55.56, 38.13, 26.84, 22.65, 14.09; HRMS (ESI+) exact mass calculated for ($C_{12}H_{16}O_2$) $[M+H]^+$ requires *m/z* 193.1223, found *m/z* 193.1221.

5.2.2 Synthesis of 1-(4-(prop-2-yn-1-yloxy) phenyl) pentan-1-one (6b) [4]:

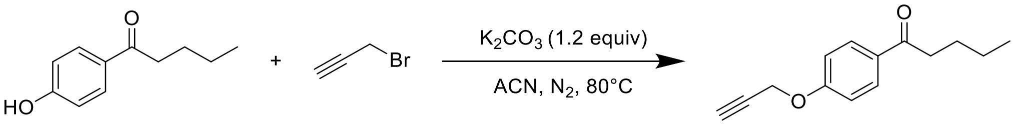

To a 50 mL double-necked round-bottom flask equipped with a reflux condenser and magnetic stir bar 1-(4-hydroxyphenyl) pentan-1-one (892 mg, 5 mmol) and potassium carbonate (830 mg, 1.2 equiv) were added. 20 mL of ACN was added to the flask via syringe, followed by 3-bromopropyne (714 mg, 1.2 equiv) under nitrogen atmosphere. The reaction mixture was heated to 80 °C and monitored by TLC. The reaction mixture was filtered to remove the potassium carbonate and the filtrate was concentrated by rotary evaporation. The crude product was purified by silica gel chromatography using hexane/ethyl acetate (10:1, v/v) as eluent to obtain 1.03 g (95%) of **6b** product as white crystal solid. ^{1}H NMR (600 MHz, $CDCl_3$) δ 7.96 (d, J = 8.8 Hz, 2H), 7.01 (d, J = 8.8 Hz, 2H), 4.75 (d, J = 2.4 Hz, 2H), 2.96–2.87 (m, 2H), 2.55 (t, J = 2.4 Hz, 1H), 1.74–1.67 (m, 2H), 1.46–1.34(m, 2H), 0.94 (t, J = 7.3 Hz, 3H); ^{13}C NMR (151 MHz, $CDCl_3$) δ 199.58, 161.41, 131.21, 130.59, 114.86, 78.12, 76.47, 56.14, 38.41, 27.00, 22.87, 14.32; HRMS (ESI+) exact mass calculated for ($C_{14}H_{16}O_2$) $[M+H]^+$ requires *m/z* 217.1223, found *m/z* 217.1216.

5.2.3 General Procedure A of Noyori Asymmetric transfer Hydrogenation [5]

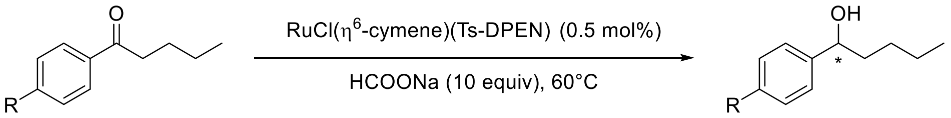

A 50 mL round-bottom flask equipped with magnetic stir bar was charged with Valerophenone (1 mmol), RuCl(η^6-cymene)(Ts-DPEN) (0.005 equiv) and HCOONa (10 equiv). 20 mL of *i*-PrOH : H_2O (2:1, v/v) was added to the flask via syringe and heated to 60 °C and refluxed overnight. The mixture was poured into a separatory funnel containing 25 mL of ethyl acetate and 25 mL of H_2O. The layers were separated and the aqueous layer was extracted with ethyl acetate (3 × 5 mL). The combined organic layers were dried (Na_2SO_4) and concentrated by rotary evaporation. The crude product was purified by silica gel chromatography using hexane/ethyl acetate (10:1, v/v) as eluent to obtain of corresponding product.

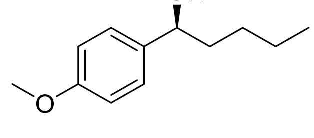

***S*-7a**

(*S*)-1-(4-methoxyphenyl)pentan-1-ol (*S*-7a):

Prepared according to the general procedure A using 1-(4-methoxyphenyl)pentan-1-one **6a** (192 mg, 1 mmol), RuCl[(1*S*,2*S*)-*N*–*p*-toluenesulfonyl-1,2-diphenylethanediamine](η^6-*p*-cymene) (830 mg, 0.005 equiv), HCOONa(680 mg, 10 equiv), and 20 mL *i*-PrOH: H_2O (2:1, v/v). The reaction mixture was subjected to the work up

protocol outlined in the general procedure and purified by flash chromatography using petroleum ether: ethyl acetate (10:1, v/v) as the eluent to afford 134 mg (69% yield, –73% *ee*) of the title compound as a colorless oil. ^{1}H NMR (600 MHz, $CDCl_3$) δ 7.34–7.17 (m, 2H), 6.98–6.73 (m, 2H), 4.58 (t, *J* = 6.8 Hz, 1H), 3.79 (s, 3H), 2.04 (s, 1H), 1.82–1.60 (m, 2H), 1.44–1.14 (m, 4H), 0.87 (t, *J* = 7.1 Hz, 3H); ^{13}C NMR (151 MHz, $CDCl_3$) δ 159.01, 137.20, 127.25, 113.83, 74.34, 55.34, 38.78, 28.15, 22.71, 14.14; HRMS (APCI+) exact mass calculated for ($C_{12}H_{18}O$) $[M+Na]^+$ requires *m/z* 217.1199, found *m/z* 217.1204. The *ee* was determined by chiral HPLC analysis using a Chiralcel OD-H (25 cm, 0.46 μm) column (Hexane: *i*-PrOH = 98:2, flow rate = 0.5 mL/min, λ = 220 nm); (*R*)-isomer: *t* = 36.0 min, (*S*)-isomer: *t* = 40.2 min.

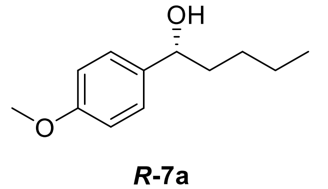

***R*-7a**

(*R*)-1-(4-methoxyphenyl)pentan-1-ol (*R*-7a):

Prepared according to the general procedure A using 1-(4-methoxyphenyl)pentan-1-one (192 mg, 1 mmol), RuCl[(1*R*,2*R*)-*N*–*p*-toluenesulfonyl-1,2-diphenylethanediamine](η^6-*p*-cymene) (830 mg, 0.005 equiv), HCOONa (680 mg, 10 equiv), and 20 mL *i*-PrOH : H_2O (2:1, v/v). The reaction mixture was subjected to the work up protocol outlined in the general procedure and purified by flash chromatography using petroleum ether: ethyl acetate (10:1, v/v) as the eluent to afford 120 mg (62% yield, 73% *ee*) of the title compound as a colorless oil. The *ee* was determined by chiral HPLC analysis using a Chiralcel OD-H (25 cm, 0.46 μm) column (Hexane: *i*-PrOH = 98:2, flow rate = 0.5 mL/min, λ = 220 nm); (*R*)-isomer: *t* = 35.9 min, (*S*)-isomer: *t* = 40.3 min.

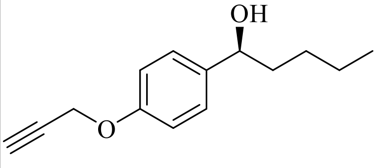

***S*-7b**

(*S*)-1-(4-(prop-2-yn-1-yloxy)phenyl)pentan-1-ol (*S*-7b):

Prepared according to the general procedure A using 1-(4-(prop-2-yn-1-yloxy) phenyl) pentan-1-one (216 mg, 1 mmol), RuCl[(1*S*,2*S*)-*N*–*p*-toluenesulfonyl-1,2-diphenylethanediamine](η^6-*p*-cymene) (830 mg, 0.005 equiv), HCOONa(680 mg, 10 equiv), and 20 mL of *i*-PrOH : H_2O(2:1, v/v). The reaction mixture was subjected to the workup protocol outlined in the general procedure and purified by flash chromatography using petroleum ether: ethyl acetate (10:1, v/v) as the eluent to afford 142 mg (65% yield, –74% *ee*) of the title compound as a colorless oil. ^{1}H NMR (600 MHz, $CDCl_3$) δ 7.32–7.26 (m, 2H), 6.99–6.92 (m, 2H), 4.69 (d, *J* = 2.4 Hz, 2H), 4.62 (t, *J* = 13.5 Hz, 1H), 2.52 (t, *J* = 2.4 Hz, 1H), 1.87–1.73 (m, 2H), 1.72–1.64 (m, 1H), 1.41–1.19 (m, 4H), 0.88 (t, *J* = 7.2 Hz, 3H); ^{13}C NMR (151 MHz, $CDCl_3$) δ 157.09, 138.20, 127.30, 114.93, 78.73, 75.64, 74.39, 55.98, 38.85, 28.17, 22.74, 14.16; HRMS (ESI+) exact mass calculated for ($C_{14}H_{18}O_2$) $[M+H–H_2O]^+$ ($C_{14}H_{17}O$) requires *m/z* 201.1274, found *m/z* 201.1273. The *ee* was determined by chiral HPLC analysis using a Chiralcel OD-H (25 cm, 0.46 μm) column (Hexane: *i*-PrOH = 90:10, flow rate = 1.0 mL/min, λ = 220 nm); (*S*)-isomer: *t* = 9.2 min, (*R*)-isomer: *t* = 10.7 min.

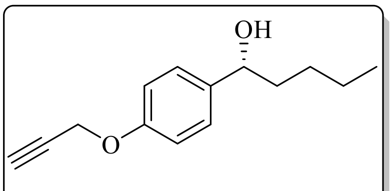

***R*-7b**

(*R*)-1-(4-(prop-2-yn-1-yloxy)phenyl)pentan-1-ol (*R*-7b):

Prepared according to the general procedure A using 1-(4-(prop-2-yn-1-yloxy) phenyl) pentan-1-one (216 mg, 1 mmol), RuCl[(1*R*,2*R*)-*N*–*p*-toluenesulfonyl-1,2-diphenylethanediamine](η^6-*p*-cymene) (830 mg, 0.005 equiv), HCOONa(680 mg, 10 equiv), and 20 mL of *i*-PrOH: H_2O (2:1, v/v). The reaction mixture was subjected to the workup protocol outlined in the general procedure and purified by flash chromatography using petroleum ether: ethyl acetate (10:1, v/v) as the eluent to afford 146 mg (67% yield, 72% *ee*) of the title compound as a colorless oil. The *ee* was determined by chiral HPLC analysis using a Chiralcel OD-H (25 cm, 0.46 μm) column (Hexane: *i*-PrOH = 90:10, flow rate = 1.0 mL/min, λ = 220 nm); (*S*)-isomer: t = 9.6 min, (*R*)-isomer: t = 11.1 min.

5.3 The Direct Asymmetric Alkylation of Aldehydes:

5.3.1 Synthesis of hept-6-ynal (3b) [6]:

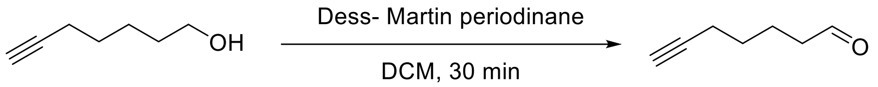

3b

To a 50 mL single-necked round-bottom flask equipped with magnetic stir bar Dess-Martin periodinane (12.73 g, 30 mmol) and 20 mL dichloromethane were added. After the reaction mixture was cooled to 0 °C by ice-water bath, hept-6-yn-1-ol (4 mL, 30 mmol) was slowly added to this suspension in 10 min. The reaction was allowed to warm up to room temperature and stirring for 30 minutes and monitored by TLC upon the completely consumption of hept-6-yn-1-ol. The reaction mixture was concentrated by rotary evaporation to remove the solvent, and 20 mL ethyl acetate was added, washed by saturated sodium hydrogen carbonate (20 mL × 3). The combine organic phase was dried by anhydrous sodium sulfate and concentrated under reduced pressure. The residue was purified by silica gel chromatography using hexane/ethyl acetate (10:1, v/v) as eluent to afford **3b** as colorless oil (2.8 g, 85%). ^{1}H NMR (600 MHz, $CDCl_3$) δ 9.77 (t, J = 1.7 Hz, 1H), 2.47 (dt, J = 7.3, 1.7 Hz, 2H), 2.22 (dt, J = 7.0, 2.7 Hz, 2H), 1.96 (t, J = 2.7 Hz, 1H), 1.80–1.72 (m, 2H), 1.61–1.54 (m, 2H); ^{13}C NMR (151 MHz, $CDCl_3$) δ 202.37, 83.91, 68.89, 43.44, 27.90, 21.22, 18.33; HRMS (ESI+) exact mass calculated for ($C_7H_{10}O$) $[M+Na]^+$ requires *m/z* 133.0624, found *m/z* 133.0619.

5.3.2 Synthesis of non-8-ynal (3d) [6]:

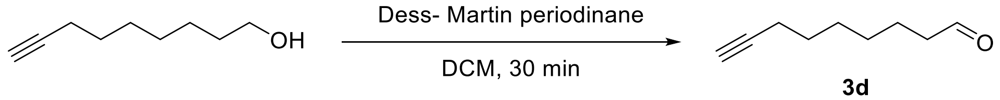

3d

To a 50 mL single-necked round-bottom flask equipped with magnetic stir bar Dess-Martin periodinane (2.12 g, 5 mmol) and 20 mL dichloromethane were added. After the reaction mixture was cooled to 0 °C by ice-water bath, non-8-yn-1-ol (825 μL, 5 mmol) was slowly added to this suspension in 10 min. The reaction was allowed to warm up to room temperature and stirring for 30 minutes and monitored by TLC upon the completely consumption of non-8-yn-1-ol. The reaction mixture was concentrated by rotary evaporation to remove the solvent, and 20 mL ethyl acetate was added, washed by saturated sodium hydrogen carbonate (20 mL × 3). The combined organic phase was

dried by anhydrous sodium sulfate and concentrated under reduced pressure. The residue was purified by silica gel chromatography using hexane/ethyl acetate (10:1, v/v) as eluent to afford **3c** as colorless oil (497 mg, 72%). ^{1}H NMR (600 MHz, $CDCl_3$) δ 9.73 (t, J = 1.8, 1H), 2.42 (dt, J = 7.2, 2.1 Hz, 2H), 2.17 (dt, J = 7.0, 2.6 Hz, 2H), 1.93 (t, J = 1.8, 1H), 1.67–1.58 (m, 2H), 1.56–1.48 (m, 2H), 1.46–1.37 (m, 2H), 1.37–1.30 (m, 2H); ^{13}C NMR (151 MHz, $CDCl_3$) δ 202.86, 84.57, 68.40, 43.92, 28.72, 28.51, 28.29, 22.02, 18.42; HRMS (ESI+) exact mass calculated for ($C_9H_{14}O$) $[M+Na]^+$ requires *m/z* 161.0937, found *m/z* 161.0928

5.3.3 Synthesis of (2*R*,5*S*)-2-tert-Butyl-3,5-dimethylimidazolidin-4-one (L3) [7]:

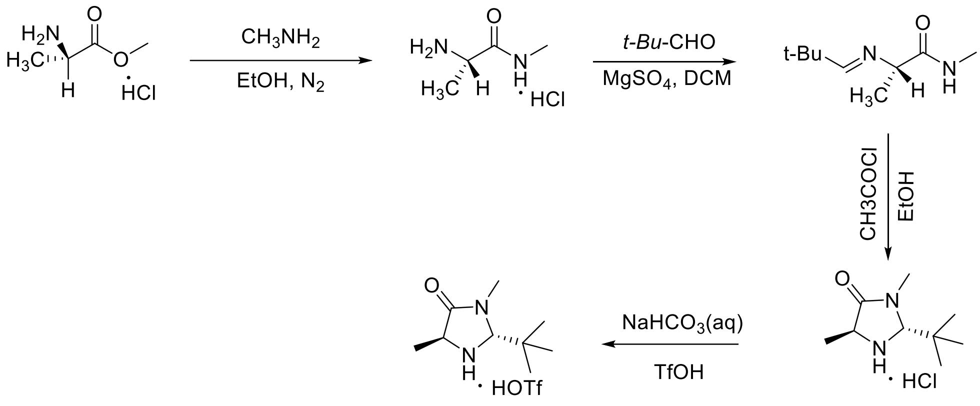

L3

According to the reported literature of Thomas H. Graham [7], (2*R*, 5*S*)-2-tert-Butyl-3,5-dimethylimidazolidin-4-one (**L3**) was obtained as white solid. ^{1}H NMR (600 MHz, CD_3OD) δ 4.76 (s, 1H), 4.27 (q, J = 7.0 Hz, 1H), 3.06 (s, 3H), 1.56 (d, J = 7.2 Hz, 3H), 1.16 (s, 9H); ^{13}C NMR (151 MHz, CD_3OD) δ 170.98, 81.76, 54.74, 37.53, 32.36, 25.26, 14.66. HRMS (ESI+) exact mass calculated for ($C_9H_{18}N_2O$) $[M+H]^+$ requires *m/z* 171.1492, found *m/z* 171.1485.

5.3.4 General procedure B for the preparation of primary amine organocatalysts:

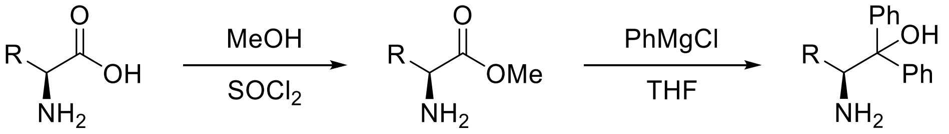

To a 50 mL single-necked round-bottom flask equipped with magnetic stir bar amino acid (1 equiv) was dissolved with MeOH (10 mL), then thionyl chloride (1.2 equiv) was dropwisely added to the flask at 0 °C, and the reaction was stirred for 12h. After the complete consumption of amino acid, the reaction mixture was concentrated by rotary evaporation to get the amino acid methyl ether crude product without further purification. The amino acid methyl ether crude product was added to a double-necked round-bottom flask and degassed via freeze pump thaw (x 3), and the tube refilled with nitrogen. Dry THF (10 mL) and Grignard reagent PhMgCl (3 equiv) were added to the reaction mixture via syringe. Monitoring the reaction by TLC, the reaction mixture distilled with ethyl acetate (20 mL), and washed with water three times. The combined organic layer was dried by anhydrous sodium sulfate and concentrated under reduced pressure. The crude product was purified by silica gel chromatography using dichloromethane/methanol (10:1, v/v) as eluent to obtain of corresponding product.

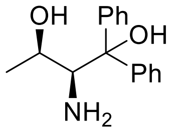

A15

(2*S*,3*R*)-2-amino-1,1-diphenylbutane-1,3-diol (A15):

Prepared according to the general procedure B using L-threonine (119 mg, 1 mmol), thionyl chloride (73 μL, 1.2 equiv), 2 M PhMgCl in THF (1.5 mL, 3 equiv) and 10 mL dry THF. The reaction mixture was purified by silica gel chromatography using dichloromethane/methanol (10:1, v/v) as the eluent to afford 136 mg (53% yield) of the title compound as pale-yellow solid. ^{1}H NMR (600 MHz, $CDCl_3$) δ 7.59–7.46 (m, 4H), 7.34–7.28 (m, 4H), 7.22–7.15 (m, 2H), 3.96–3.88 (m, 1H), 3.73–3.67 (m, 1H), 1.22 (d, *J* = 6.4 Hz, 3H). ^{13}C NMR (151 MHz, $CDCl_3$) δ 145.38, 144.41, 128.77, 128.65, 127.16, 126.95, 125.64, 125.21, 81.51, 67.06, 59.09, 21.51. HRMS (ESI+) exact mass calculated for ($C_{16}H_{19}NO_2$) $[M+H]^+$ requires *m/z* 258.1489, found *m/z* 258.1484.

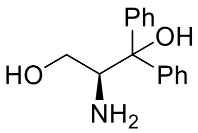

A18

(*S*)-2-amino-1,1-diphenylpropane-1,3-diol (A18):

Prepared according to the general procedure B using L-serine (105 mg, 1 mmol), thionyl chloride (73 μL, 1.2 equiv), 2 M PhMgCl in THF (1.5 mL, 3 equiv) and 10 mL dry THF. The reaction mixture was purified by silica gel chromatography using dichloromethane/methanol (10:1, v/v) as the eluent to afford 109 mg (45% yield) of the title compound as pale-yellow oil. ^{1}H NMR (600 MHz, $CDCl_3$) δ 7.55–7.40 (m, 4H), 7.32–7.23 (m, 4H), 7.21–7.11 (m, 2H), 3.85 (dd, *J* = 5.7, 3.3 Hz, 1H), 3.57 (qd, *J* = 11.2, 4.4 Hz, 2H). ^{13}C NMR (151 MHz, $CDCl_3$) δ 145.56, 144.78, 128.63, 128.37, 126.99, 126.81, 125.53, 125.20, 79.71, 63.12, 56.97. HRMS (ESI+) exact mass calculated for ($C_{15}H_{17}NO_2$) $[M+H]^+$ requires *m/z* 244.1332, found *m/z* 244.1331.

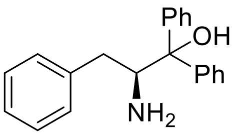

A21

(*S*)-2-amino-1,1,3-triphenylpropan-1-ol (A21):

Prepared according to the general procedure B using L-phenylalanine (165 mg, 1 mmol), thionyl chloride (73 μL, 1.2 equiv), 2 M PhMgCl in THF (1.5 mL, 3 equiv) and 10 mL dry THF. The reaction mixture was purified by silica gel chromatography using dichloromethane/methanol (10:1, v/v) as the eluent to afford 224 mg (74% yield) of the title compound as white solid. ^{1}H NMR (600 MHz, $CDCl_3$) δ 7.74–7.54 (m, 4H), 7.38–7.28 (m, 6H), 7.24–7.15 (m, 5H), 4.18 (dd, *J* = 10.8, 2.6 Hz, 1H), 2.65 (dd, *J* = 13.9, 2.5 Hz, 1H), 2.46 (dd, *J* = 13.9, 10.8 Hz, 1H). ^{13}C NMR (151 MHz, $CDCl_3$) δ 146.89, 144.42, 139.71, 129.26, 128.85, 128.68, 128.42, 126.96, 126.72, 126.65, 125.94, 125.54, 78.66, 58.42, 36.85. HRMS (ESI+) exact mass calculated for ($C_{21}H_{21}NO$) $[M+H]^+$ requires *m/z* 304.1696, found *m/z* 304.1692.

5.3.5 General procedure C for the preparation of DPEN-based primary amine organocatalysts [2]:

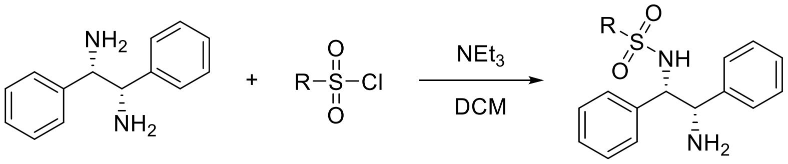

To a 50 mL single-necked round-bottom flask equipped with magnetic stir bar (1*S*,2*S*)-1,2-diphenylethane-1,2-diamine (1 equiv) and 20 mL of dry dichloromethane were added. Then triethylamine (1.2 equiv) was added to the flask via pipette, the reaction mixture turned to pale yellow, followed by corresponding sulfonyl chloride or acyl chloride (1 equiv). The reaction was stirred at room temperature for 30 min and monitored by TLC. Upon the completely consumption of diamine, the reaction mixture was washed with $NaHCO_3$, water, and ethyl acetate, and the combined organic layer was dried over Na_2SO_4 and concentrated by rotary evaporation. The crude product was purified by silica gel chromatography using hexane/ethyl acetate as eluent to obtain the desire product.

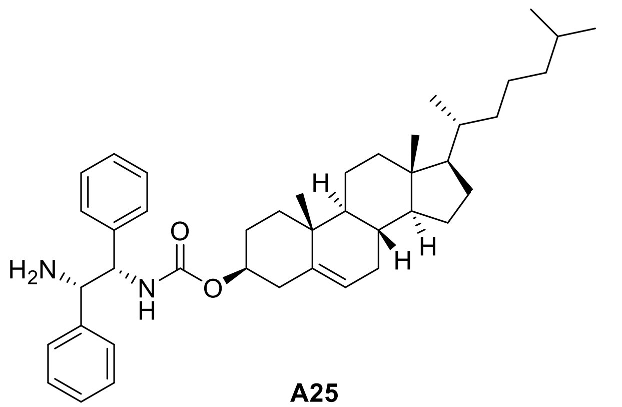

A25

Cholesteryl *N*- ((1*S*,2*S*)-2-amino-1,2-diphenylethyl) carbamate (A25):

Prepared according to the general procedure C using (1*S*,2*S*)-1,2-diphenylethane-1,2-diamine (424 mg, 2 mmol), triethylamine (334 μL, 1.2 equiv), Cholesteryl chloroformate (899 mg, 1 equiv) and 20 mL dry DCM. The reaction mixture was purified by silica gel chromatography using petroleum ether: ethyl acetate (2:1, v/v) and ethyl acetate as the eluent to afford 512 mg (41% yield) of the title compound as white solid. ^{1}H NMR (600 MHz, $CDCl_3$) δ 7.42–7.10 (m, 10H), 5.97 (d, *J* = 8.5 Hz, 1H), 5.38–5.24 (m, 1H), 4.90 (d, *J* = 6.6 Hz, 1H), 4.38 (d, *J* = 4.0 Hz, 2H), 2.28 (d, *J* = 11.8 Hz, 2H), 2.06–1.77 (m, 5H), 1.60–1.21 (m, 12H), 1.20–0.96 (m, 12H), 0.96–0.82 (m, 11H), 0.68 (s, 3H); ^{13}C NMR (151 MHz, CDCl3) δ 156.01, 142.07, 139.99, 129.03, 128.66, 128.50, 127.65, 127.42, 126.94, 126.65, 126.59, 122.46, 74.46, 60.05, 56.80, 56.24, 50.07, 42.42, 39.85, 39.64, 38.54, 37.04, 36.64, 36.31, 35.93, 32.00, 31.97, 28.36, 28.19, 28.14, 24.40, 23.96, 22.96, 22.70, 21.14, 19.45, 18.84, 12.00, 11.98; HRMS (ESI+) exact mass calculated for ($C_{42}H_{60}N_2O_2$) $[M+H]^+$ requires *m/z* 625.4728, found *m/z* 625.4715.

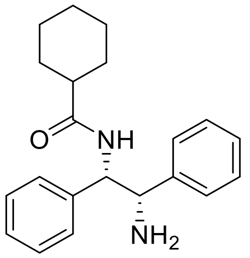

A26

***N*-((1*S*,2*S*)-2-amino-1,2-diphenylethyl) cyclohexanecarboxamide (A26):**

Prepared according to the general procedure C using (1*S*,2*S*)-1,2-diphenylethane-1,2-diamine (424 mg, 2 mmol), triethylamine (334 μL, 1.2 equiv), cyclohexanecarbonyl chloride (268 μL, 1 equiv) and 20 mL dry DCM. The reaction mixture was purified by silica gel chromatography using petroleum ether: ethyl acetate (2:1, v/v) as the eluent to afford 258 mg (40% yield) of the title compound as white solid. ^{1}H NMR (600 MHz, $CDCl_3$) δ 7.46–7.00 (m, 10H), 5.16 (dd, *J* = 8.1, 3.9 Hz, 1H), 4.45 (d, *J* = 3.9 Hz, 1H), 2.14–2.09 (m, 1H), 1.87–1.56 (m, 5H), 1.40–1.12 (m, 5H); ^{13}C NMR (151 MHz, $CDCl_3$) δ 175.87, 140.60, 128.75, 128.55, 127.77, 127.48, 126.76, 126.52, 59.48, 57.98, 45.58, 29.75, 29.74, 25.89, 25.86, 25.84; HRMS (ESI+) exact mass calculated for ($C_{21}H_{26}N_2O$) $[M+H]^+$ requires *m/z* 323.2118, found *m/z* 323.2111.

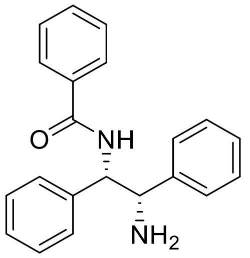

A27

***N*-((1*S*,2*S*)-2-amino-1,2-diphenylethyl) benzamide (A27):**

Prepared according to the general procedure C using (1*S*,2*S*)-1,2-diphenylethane-1,2-diamine (424 mg, 2 mmol), triethylamine (334 μL, 1.2 equiv), benzoyl chloride (241 μL, 1 equiv) and 20 mL dry DCM. The reaction mixture was purified by silica gel chromatography using petroleum ether: ethyl acetate (2:1, v/v) as the eluent to afford 228 mg (36% yield) of the title compound as white solid. ^{1}H NMR (600 MHz, $CDCl_3$) δ 7.73–7.60 (m, 3H), 7.43–7.36 (m, 1H), 7.35–7.30 (m, 4H), 7.27–7.21 (m, 5H), 7.19–7.13 (m, 2H), 5.23 (dd, *J* = 7.8, 3.6 Hz, 1H), 4.42 (d, *J* = 3.6 Hz, 1H); ^{13}C NMR (151 MHz, $CDCl_3$) δ 167.00, 141.96, 140.56, 134.60, 131.49, 128.78, 128.63, 128.60, 127.76, 127.52, 127.11, 126.61, 126.49, 59.63, 59.03; HRMS (ESI+) exact mass calculated for ($C_{20}H_{20}N_2O_2$) $[M+H]^+$ requires *m/z* 317.1648, found *m/z* 317.1639.

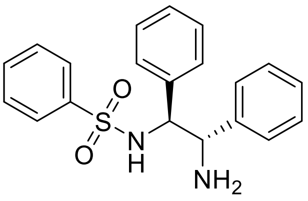

A28

***N*-((1*S*,2*S*)-2-amino-1,2-diphenylethyl) benzenesulfonamide (A28):**

Prepared according to the general procedure C using (1*S*,2*S*)-1,2-diphenylethane-1,2-diamine (424 mg, 2 mmol),

triethylamine (334 μL, 1.2 equiv), benzenesulfonyl chloride (256 μL, 1 equiv) and 20 mL dry DCM. The reaction mixture was purified by silica gel chromatography using petroleum ether: ethyl acetate (2:1, v/v) and ethyl acetate as the eluent to afford 324 mg (46% yield) of the title compound as white solid. ^{1}H NMR (600 MHz, $CDCl_3$) δ 7.44 (d, *J* = 7.7 Hz, 2H), 7.33 (t, *J* = 7.4 Hz, 1H), 7.20–7.04 (m, 12H), 4.48 (d, *J* = 6.1 Hz, 1H), 4.24 (d, *J* = 6.1 Hz, 1H); ^{13}C NMR (151 MHz, $CDCl_3$) δ 140.73, 140.31, 138.75, 131.96, 128.60, 128.34, 127.86, 127.55, 127.22, 126.95, 126.86, 63.34, 60.48; HRMS (ESI+) exact mass calculated for ($C_{20}H_{20}N_2O_2S$) $[M+H]^+$ requires *m/z* 353.1318, found *m/z* 353.1304.

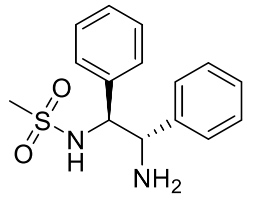

A29

***N*-((1*S*,2*S*)-2-amino-1,2-diphenylethyl) methanesulfonamide (Ms-DPEN, A29):**

Prepared according to the general procedure C using (1*S*,2*S*)-1,2-diphenylethane-1,2-diamine (424 mg, 2 mmol), triethylamine (334 μL, 1.2 equiv), methanesulfonyl chloride (155 μL, 1 equiv) and 20 mL dry DCM. The reaction mixture was purified by silica gel chromatography using petroleum ether: ethyl acetate (2:1, v/v) as the eluent to afford 280 mg (48% yield) of the title compound as white solid. ^{1}H NMR (600 MHz, $CDCl_3$) δ 7.43–7.08 (m, 10H), 4.59 (d, *J* = 6.5 Hz, 1H), 4.37 (d, *J* = 6.5 Hz, 1H), 2.27 (s, 3H); ^{13}C NMR (151 MHz, $CDCl_3$) δ 140.38, 139.20, 128.84, 128.80, 128.22, 128.08, 127.31, 127.20, 63.36, 60.09, 41.06; HRMS (ESI+) exact mass calculated for ($C_{15}H_{18}N_2O_2S$) $[M+H]^+$ requires *m/z* 291.1162, found *m/z* 291.1149.

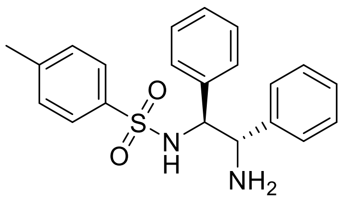

A30

***N*-((1*S*,2*S*)-2-amino-1,2-diphenylethyl)-4-methylbenzenesulfonamde (Ts-DPEN, A30):**

Prepared according to the general procedure C using (1*S*,2*S*)-1,2-diphenylethane-1,2-diamine (424 mg, 2 mmol), triethylamine (334 μL, 1.2 equiv), 4-methylbenzenesulfonyl chloride (381 mg, 1 equiv) and 20 mL dry DCM. The reaction mixture was purified by silica gel chromatography using petroleum ether: ethyl acetate (2:1, v/v) and ethyl acetate as the eluent to afford 320 mg (44% yield) of the title compound as white solid. ^{1}H NMR (600 MHz, $CDCl_3$) δ 7.34–7.30 (m, 2H), 7.17–7.10 (m, 8H), 6.96 (dd, *J* = 8.3, 2.3 Hz, 2H), 4.42 (t, *J* = 6.3 Hz, 1H), 4.19 (t, *J* = 8.4 Hz, 1H), 2.31 (s, 3H); ^{13}C NMR (151 MHz, $CDCl_3$) δ 142.60, 141.34, 139.27, 137.27, 129.22, 128.52, 128.33, 127.58, 127.45, 127.16, 126.96, 126.72, 63.33, 60.58, 21.52; HRMS (ESI+) exact mass calculated for ($C_{21}H_{22}N_2O_2S$) $[M+H]^+$ requires *m/z* 367.1475, found *m/z* 367.1459.

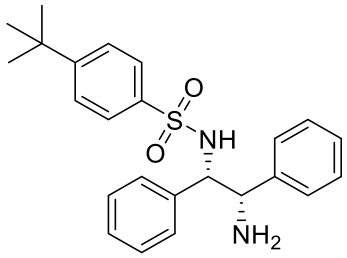

A31

***N*-((1*S*,2*S*)-2-amino-1,2-diphenylethyl)-4-(tert-butyl) benzenesulfonamide (A31):**

Prepared according to the general procedure C using (1*S*,2*S*)-1, 2-diphenylethane-1,2-diamine (424 mg, 2 mmol), triethylamine (334 μL, 1.2 equiv), 4-(tert-butyl) benzenesulfonyl chloride (466 mg, 1 equiv) and 20 mL dry DCM. The reaction mixture was purified by silica gel chromatography using petroleum ether: ethyl acetate (2:1, v/v) and ethyl acetate as the eluent to afford 367mg (45% yield) of the title compound as white solid. ^{1}H NMR (600 MHz, $CDCl_3$) δ 7.39–7.30 (m, 2H), 7.21–7.03 (m, 11H), 4.41 (d, *J* = 5.6 Hz, 1H), 4.16 (d, *J* = 5.6 Hz, 1H), 1.27 (s, 9H); ^{13}C NMR (151 MHz, $CDCl_3$) δ 155.49, 141.42, 139.12, 137.12, 128.49, 128.29, 127.68, 127.42, 127.14, 126.82, 126.75, 125.53, 63.37, 60.58, 35.00, 31.17; HRMS (ESI+) exact mass calculated for ($C_{24}H_{28}N_2O_2S$) $[M+H]^+$ requires *m/z* 409.1944, found *m/z* 409.1931.

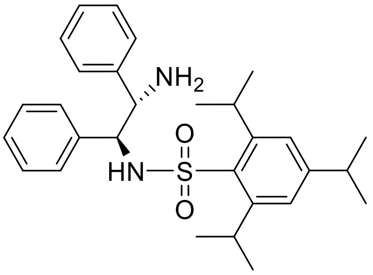

A32

***N*-((1*S*,2*S*)-2-amino-1,2-diphenylethyl)-2,4,6-triisopropylbenzenesulfonamide (A32):**

Prepared according to the general procedure C using (1*S*,2*S*)-1,2-diphenylethane-1,2-diamine (424 mg, 2 mmol), triethylamine (334 μL, 1.2 equiv), 2, 4, 6-triisopropylbenzenesulfonyl chloride (606 mg, 1 equiv) and 20 mL dry DCM. The reaction mixture was purified by silica gel chromatography using petroleum ether: ethyl acetate (2:1, v/v) and ethyl acetate as the eluent to afford 230 mg (48% yield) of the title compound as white solid. ^{1}H NMR (600 MHz, $CDCl_3$) δ 7.20–7.12 (m, 3H), 7.11–7.03 (m, 2H), 7.02–6.88 (m, 5H), 6.84–6.78 (m, 2H), 4.59–4.50 (m, 1H), 4.08–3.88 (m, 3H), 2.87–2.77 (m, 1H), 1.22–1.07 (m, 18H); ^{13}C NMR (151 MHz, $CDCl_3$) δ 152.45, 149.80, 141.98, 138.75, 134.09, 128.51, 128.51, 127.97, 127.97, 127.66, 127.48, 127.48, 127.48, 127.42, 126.99, 123.41, 63.67, 61.30, 34.27, 29.93, 25.04, 24.90, 23.82, 23.79; HRMS (ESI+) exact mass calculated for ($C_{29}H_{38}N_2O_2S$) $[M+H]^+$ requires *m/z* 479.2727, found *m/z* 479.2711.

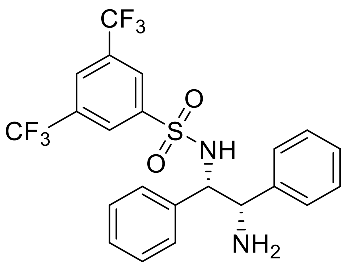

***N*-((1*S*,2*S*)-2-amino-1,2-diphenylethyl)-3,5-bis(trifluoromethyl) benzenesulfonamide (A33):**

Prepared according to the general procedure C using (1*S*,2*S*)-1,2-diphenylethane-1,2-diamine (424 mg, 2 mmol), triethylamine (334 μL, 1.2 equiv), 3,5-bis(trifluoromethyl)benzenesulfonyl chloride (625 mg, 1 equiv) and 20 mL dry DCM. The reaction mixture was purified by silica gel chromatography using petroleum ether: ethyl acetate (2:1, v/v) and ethyl acetate as the eluent to afford 606 mg (62% yield) of the title compound as white solid. ^{1}H NMR (600 MHz, $CDCl_3$) δ 7.87 (s, 2H), 7.78 (s, 1H), 7.16–7.06 (m, 10H), 4.55 (d, *J* = 5.5 Hz, 1H), 4.23 (d, *J* = 5.6 Hz, 1H); ^{13}C NMR (151 MHz, $CDCl_3$) δ 143.28, 138.00, 132.63, 132.40, 132.17, 131.95, 128.70, 128.64, 128.11, 128.07, 127.24, 127.22, 127.12, 126.43, 125.59, 123.40, 121.59, 119.78, 63.53, 60.12; HRMS (ESI+) exact mass calculated for ($C_{22}H_{18}F_6N_2O_2S$) $[M+H]^+$ requires *m/z* 489.1066, found *m/z* 489.1051.

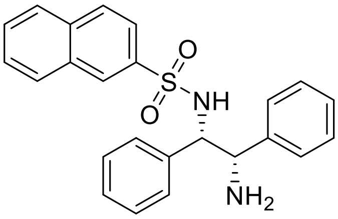

***N*-((1*S*,2*S*)-2-amino-1,2-diphenylethyl) naphthalene-2-sulfonamide (A34):**

Prepared according to the general procedure C using (1*S*,2*S*)-1,2-diphenylethane-1,2-diamine (424 mg, 2 mmol), triethylamine (334 μL, 1.2 equiv), naphthalene-2-sulfonyl chloride (454 mg, 1 equiv) and 20 mL dry DCM. The reaction mixture was purified by silica gel chromatography using petroleum ether: ethyl acetate (2:1, v/v) and ethyl acetate as the eluent to afford 330 mg (41% yield) of the title compound as white solid. ^{1}H NMR (600 MHz, $CDCl_3$) δ 7.97 (d, *J* = 1.9 Hz, 1H), 7.79 (d, *J* = 8.2 Hz, 1H), 7.71 (d, *J* = 8.7 Hz, 1H), 7.62 (d, *J* = 8.7 Hz, 1H), 7.60–7.55 (m, 1H), 7.54–7.48 (m, 1H), 7.44 (dd, *J* = 8.6, 1.9 Hz, 1H), 7.16–7.11 (m, 2H), 7.11–7.04 (m, 4H), 7.04–6.93 (m, 4H), 4.51 (d, *J* = 5.6 Hz, 1H), 4.20 (d, *J* = 5.6 Hz, 1H); ^{13}C NMR (151 MHz, $CDCl_3$) δ 140.98, 139.03, 137.09, 134.57, 132.02, 129.32, 128.96, 128.48, 128.44, 128.32, 128.29, 127.74, 127.65, 127.56, 127.12, 127.08, 126.59, 122.30, 63.40, 60.45; HRMS (ESI+) exact mass calculated for ($C_{24}H_{22}N_2O_2S$) $[M+H]^+$ requires *m/z* 403.1475, found *m/z* 403.1465.

6. Chiral HPLC data

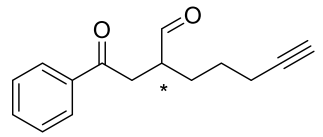

L1-5b

Signal 1: VWD1 B, Wavelength=254 nm

Peak #	RetTime [min]	Type	Width [min]	Area [mAU*s]	Height [mAU]	Area %
1	19.370	MM	0.3990	1160.37122	48.46618	50.1083
2	24.588	MM	0.5066	1155.35315	38.00904	49.8917
Totals :				2315.72437	86.47522	

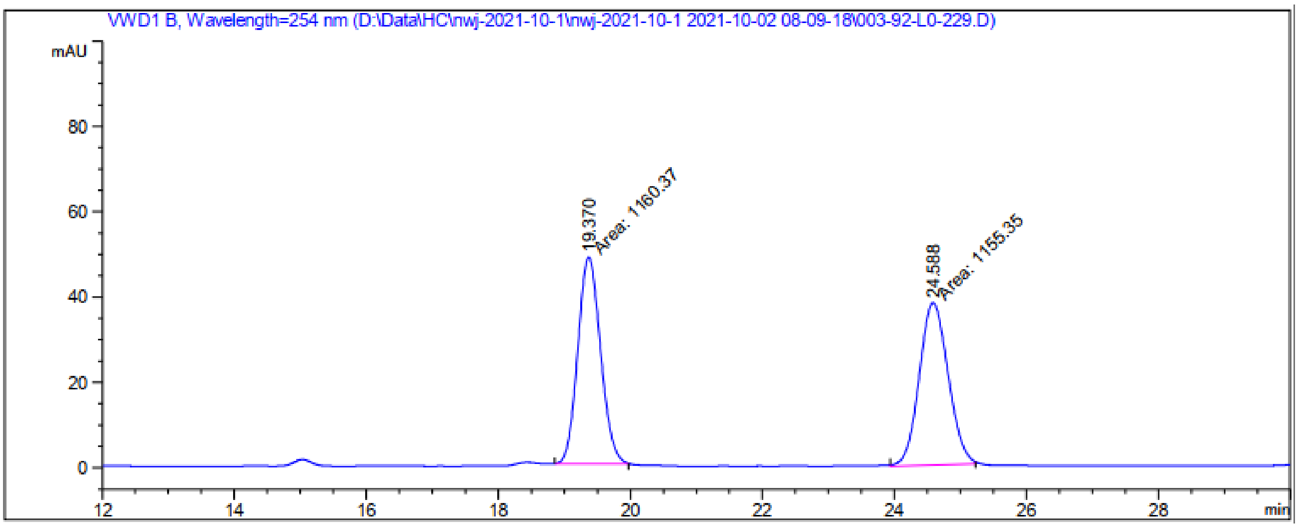

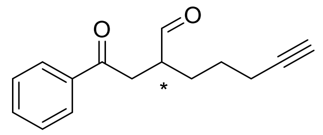

L2-5b

Signal 1: VWD1 B, Wavelength=254 nm

Peak #	RetTime [min]	Type	Width [min]	Area [mAU*s]	Height [mAU]	Area %
1	19.392	MM	0.3969	1600.32703	67.19326	41.5745
2	24.604	MM	0.5115	2248.97339	73.28675	58.4255
Totals :				3849.30042	140.48001	

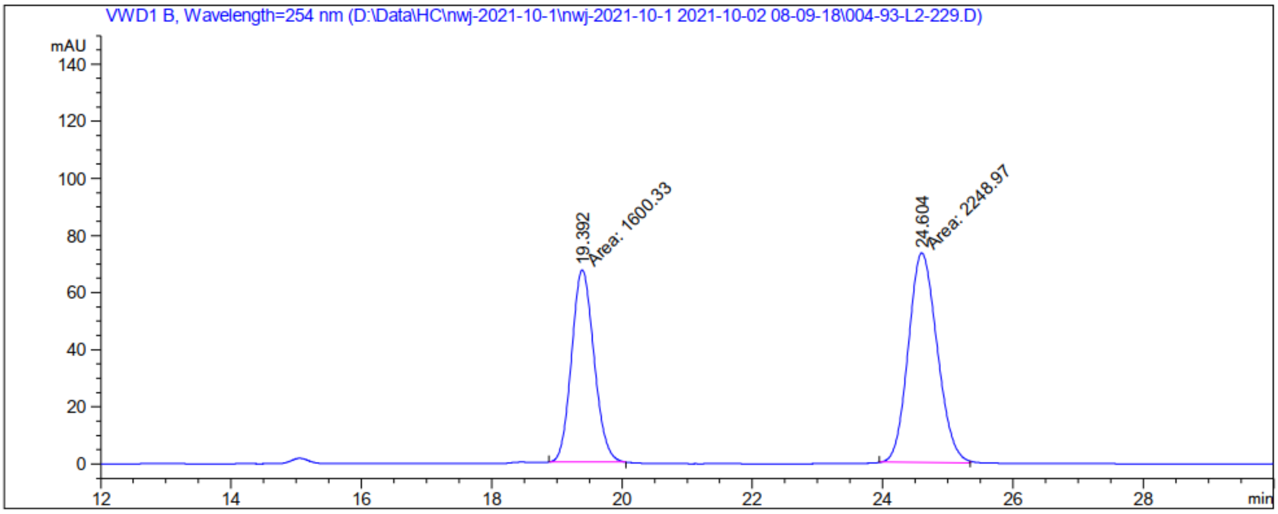

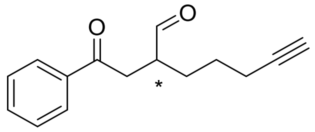

L3-5b

Signal 1: VWD1 B, Wavelength=254 nm

Peak #	RetTime [min]	Type	Width [min]	Area [mAU*s]	Height [mAU]	Area %
1	19.341	MM	0.4009	648.41901	26.95647	15.1266
2	24.486	MM	0.5183	3638.18604	116.98744	84.8734

Totals : 4286.60504 143.94391

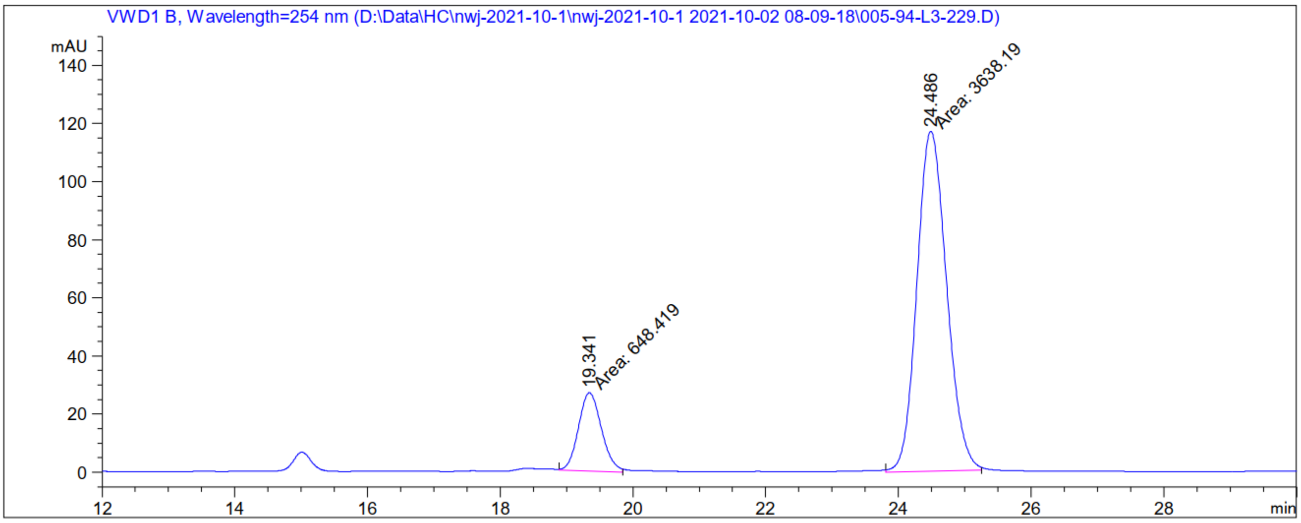

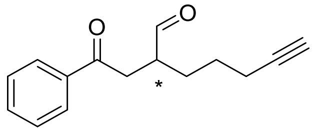

L4-5b

Signal 1: VWD1 B, Wavelength=254 nm

Peak #	RetTime [min]	Type	Width [min]	Area [mAU*s]	Height [mAU]	Area %
1	19.255	MM	0.4067	3246.40161	133.04265	74.2251
2	24.420	MM	0.5155	1127.32568	36.44425	25.7749

Totals : 4373.72729 169.48690

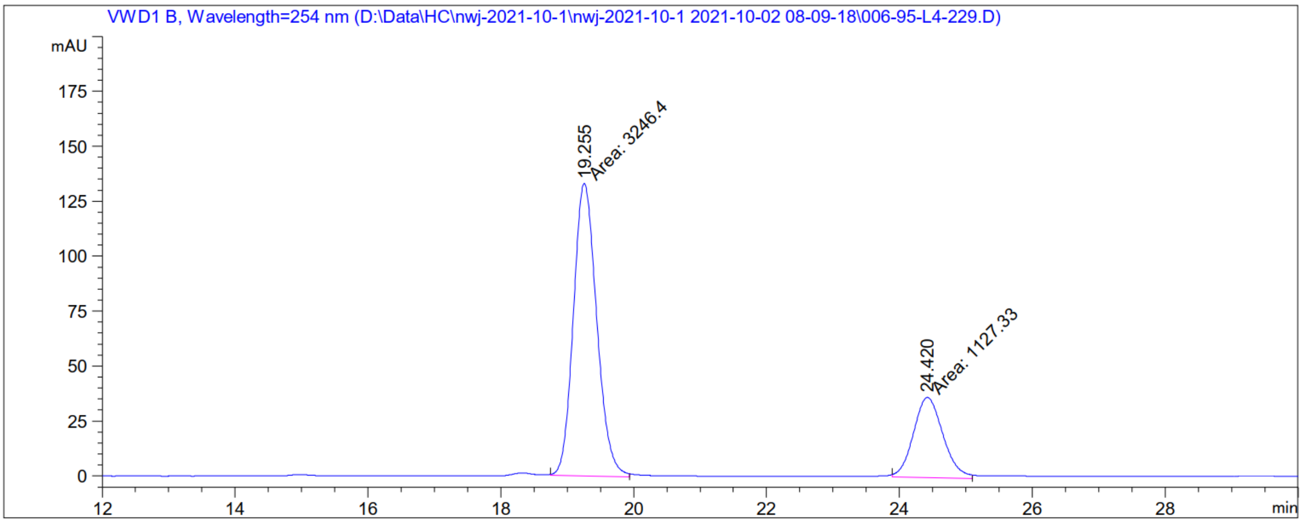

Signal 1: VWD1 B, Wavelength=254 nm

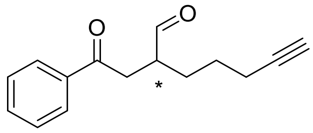

L5-5b

Peak #	RetTime [min]	Type	Width [min]	Area [mAU*s]	Height [mAU]	Area %
1	19.130	MM	0.4062	1790.87183	73.48791	53.3678
2	24.203	MM	0.5009	1564.84607	52.06800	46.6322
Totals :				3355.71790	125.55590	

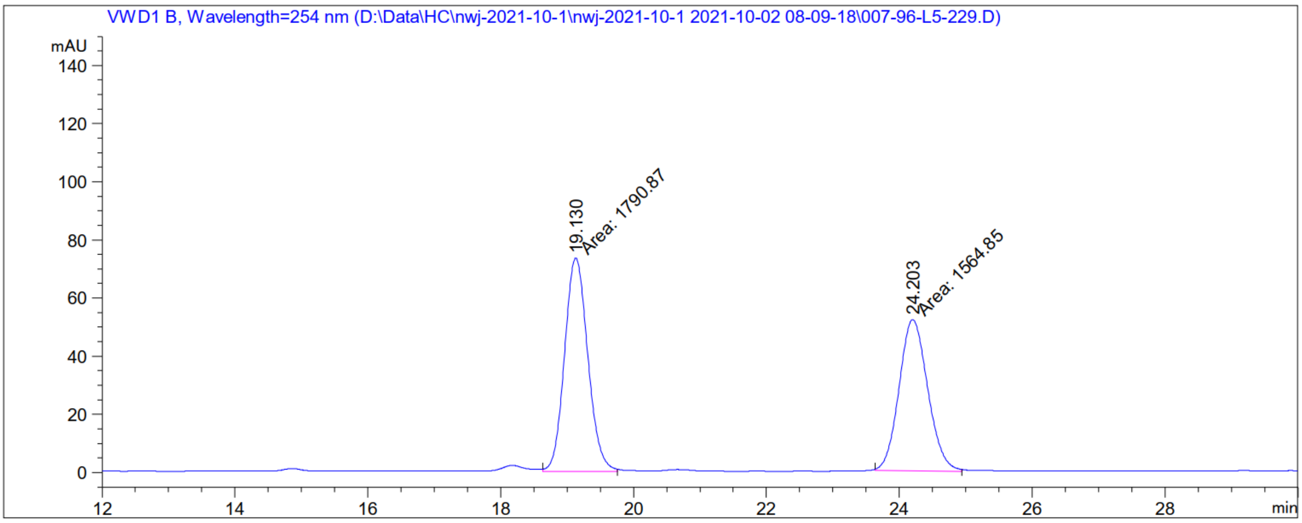

Signal 1: VWD1 B, Wavelength=254 nm

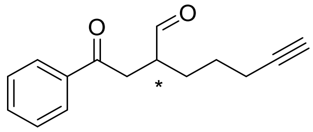

L6-5b

Peak #	RetTime [min]	Type	Width [min]	Area [mAU*s]	Height [mAU]	Area %
1	19.081	MM	0.3584	106.02132	4.93057	20.7509
2	24.133	MM	0.4933	404.90308	13.68046	79.2491
Totals :				510.92440	18.61103	

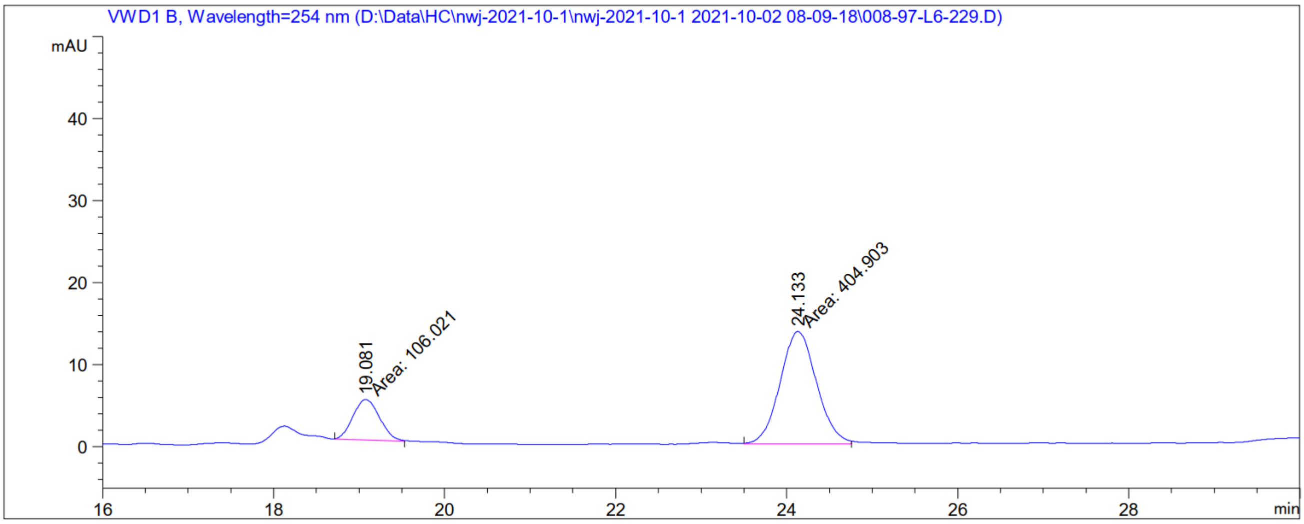

Signal 1: VWD1 B, Wavelength=254 nm

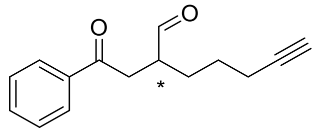

L12-5b

Peak #	RetTime [min]	Type	Width [min]	Area [mAU*s]	Height [mAU]	Area %
1	18.966	MM	0.3862	930.85986	40.16780	47.2588
2	23.959	MM	0.4937	1038.84668	35.06691	52.7412
Totals :				1969.70654	75.23471	

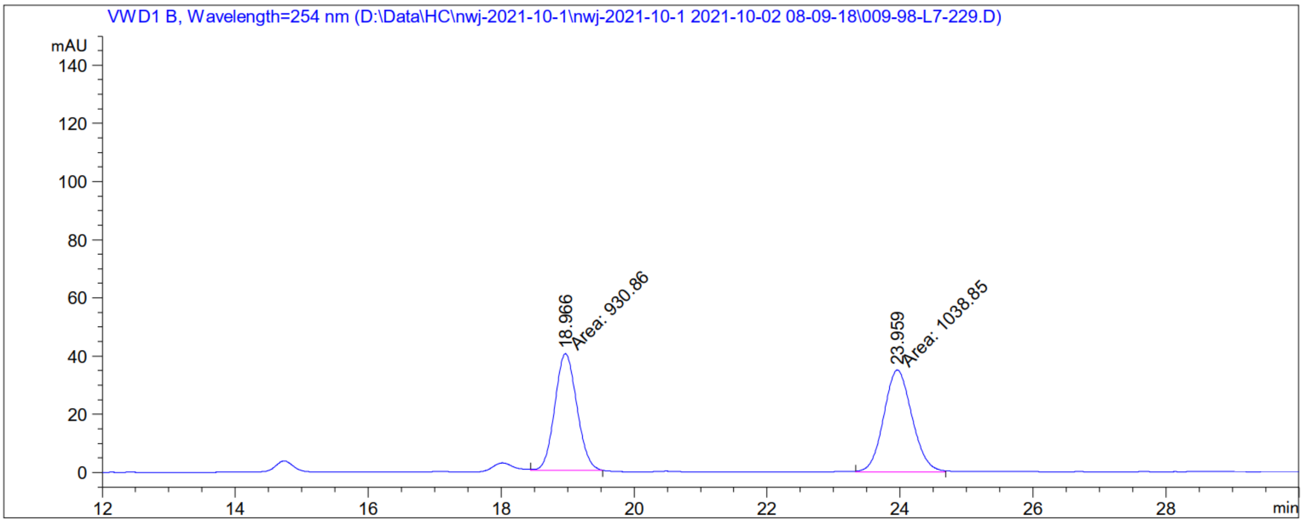

Signal 1: VWD1 B, Wavelength=254 nm

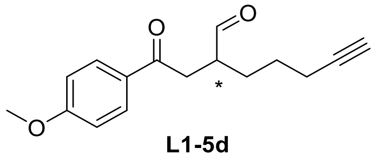

L1-5d

Peak #	RetTime [min]	Type	Width [min]	Area [mAU*s]	Height [mAU]	Area %
1	49.879	BB	0.9880	3405.94531	54.13810	50.5472
2	54.490	BB	1.0928	3332.20947	48.06826	49.4528
Totals :				6738.15479	102.20636	

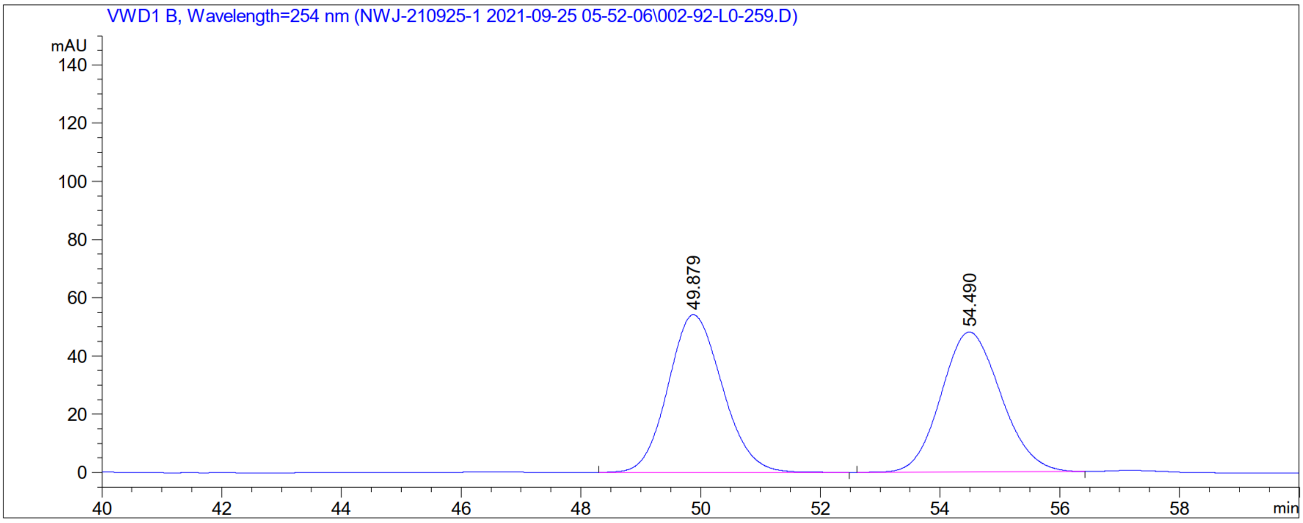

Signal 1: VWD1 B, Wavelength=254 nm

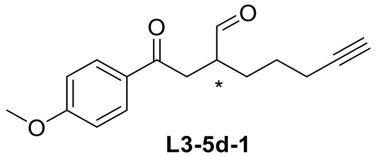
L3-5d-1

Peak #	RetTime [min]	Type	Width [min]	Area [mAU*s]	Height [mAU]	Area %
1	49.680	MM	1.0586	1205.28174	18.97606	15.5965
2	54.194	MM	1.1905	6522.62158	91.31143	84.4035

Totals : 7727.90332 110.28749

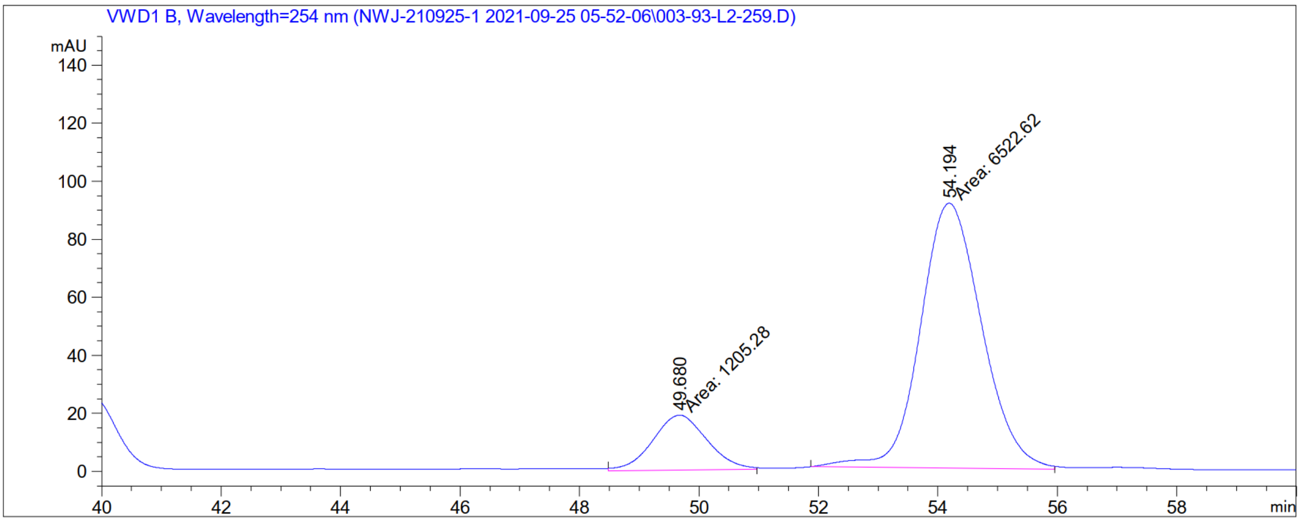

Signal 1: VWD1 B, Wavelength=254 nm

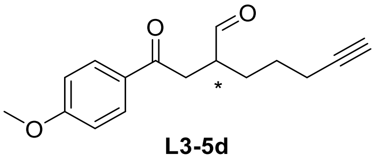
L3-5d

Peak #	RetTime [min]	Type	Width [min]	Area [mAU*s]	Height [mAU]	Area %
1	49.889	MM	1.0825	1220.33911	18.78965	16.2084
2	54.435	MM	1.1902	6308.71777	88.34576	83.7916

Totals : 7529.05688 107.13541

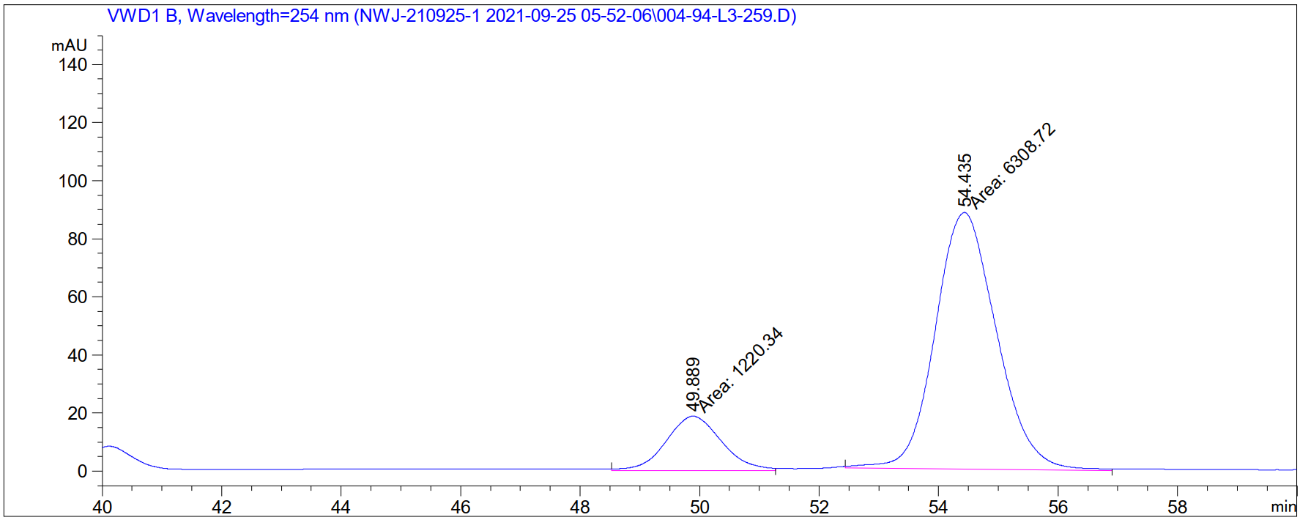

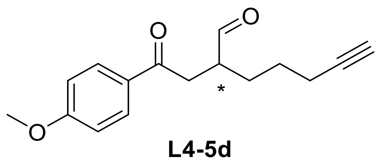

L4-5d

Signal 1: VWD1 B, Wavelength=254 nm

Peak #	RetTime [min]	Type	Width [min]	Area [mAU*s]	Height [mAU]	Area %
1	49.645	BB	0.9681	9381.27832	151.15567	79.1132
2	54.311	BB	1.0766	2476.76221	35.91512	20.8868
Totals :				1.18580e4	187.07079	

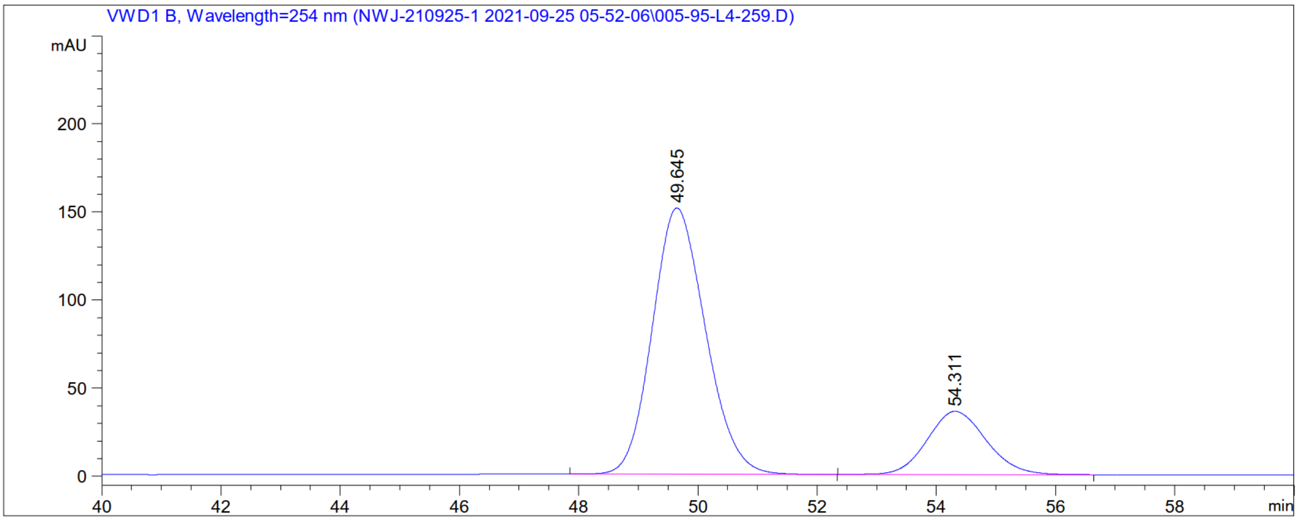

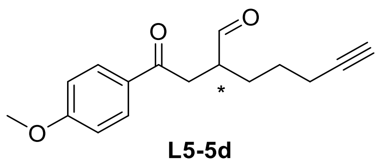

L5-5d

Signal 1: VWD1 B, Wavelength=254 nm

Peak #	RetTime [min]	Type	Width [min]	Area [mAU*s]	Height [mAU]	Area %
1	49.374	MM	1.0425	6232.62207	99.64343	53.4948
2	53.826	MM	1.1025	5418.28174	81.90585	46.5052
Totals :				1.16509e4	181.54929	

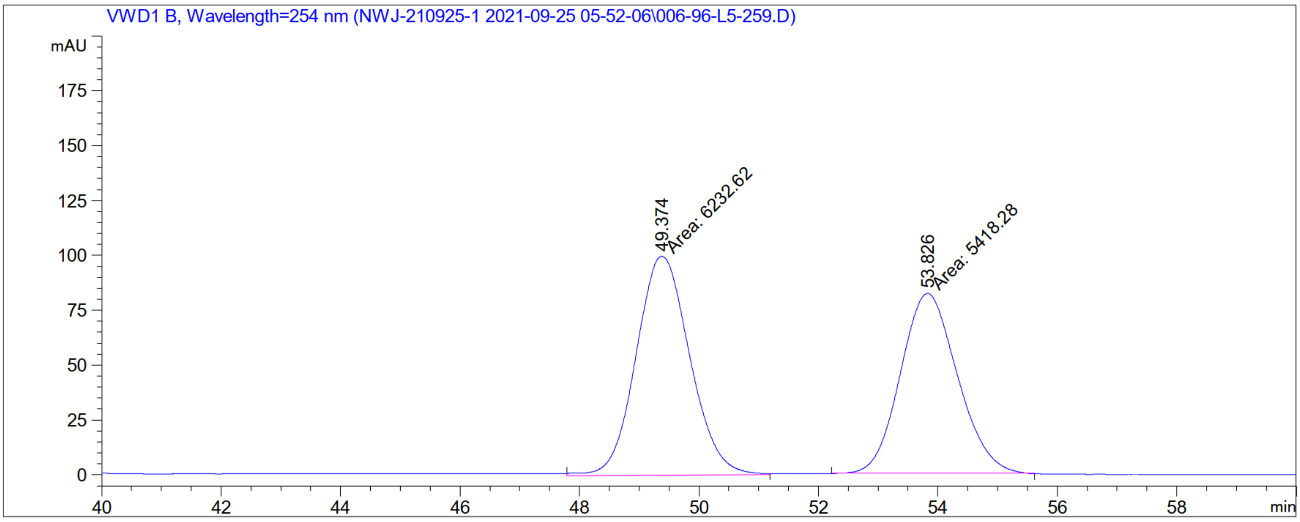

Signal 1: VWD1 B, Wavelength=254 nm

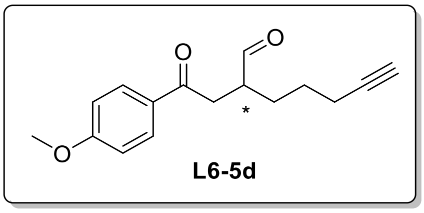

Peak #	RetTime [min]	Type	Width [min]	Area [mAU*s]	Height [mAU]	Area %
1	47.456	MM	1.0252	392.60043	6.38270	27.4034
2	51.749	MM	1.0861	1040.07080	15.96074	72.5966
Totals :				1432.67123	22.34344	

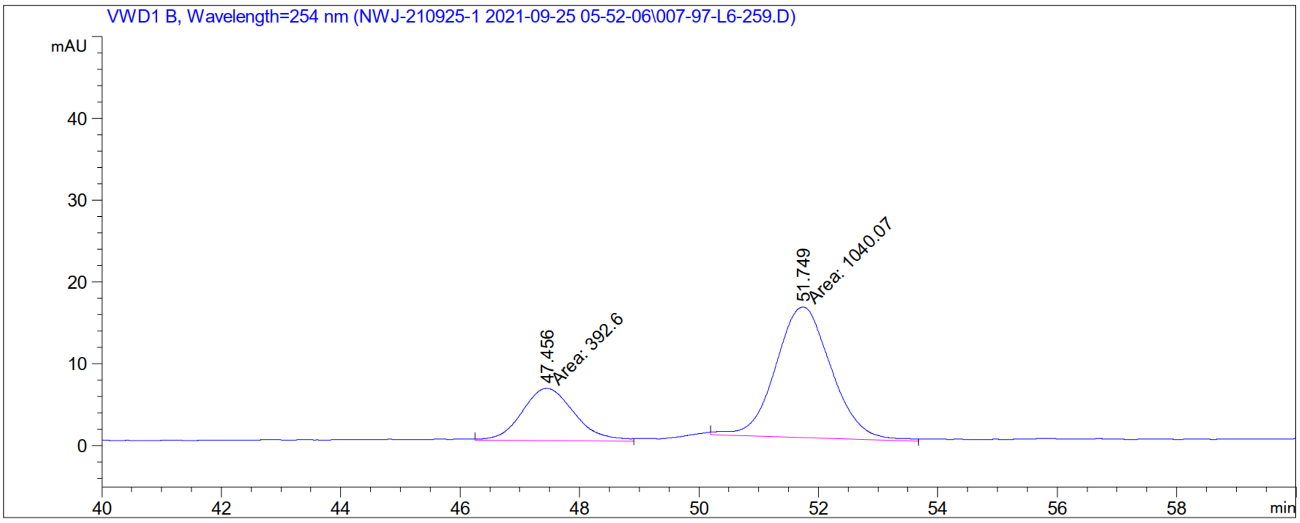

Signal 1: VWD1 B, Wavelength=254 nm

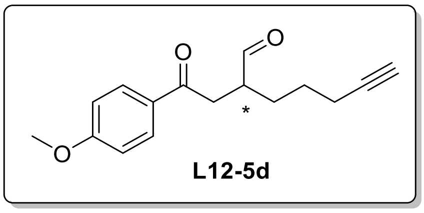

Peak #	RetTime [min]	Type	Width [min]	Area [mAU*s]	Height [mAU]	Area %
1	46.434	MM	0.9595	4829.73291	83.89601	41.5220
2	50.573	MM	1.0481	6801.99805	108.16827	58.4780
Totals :				1.16317e4	192.06429	

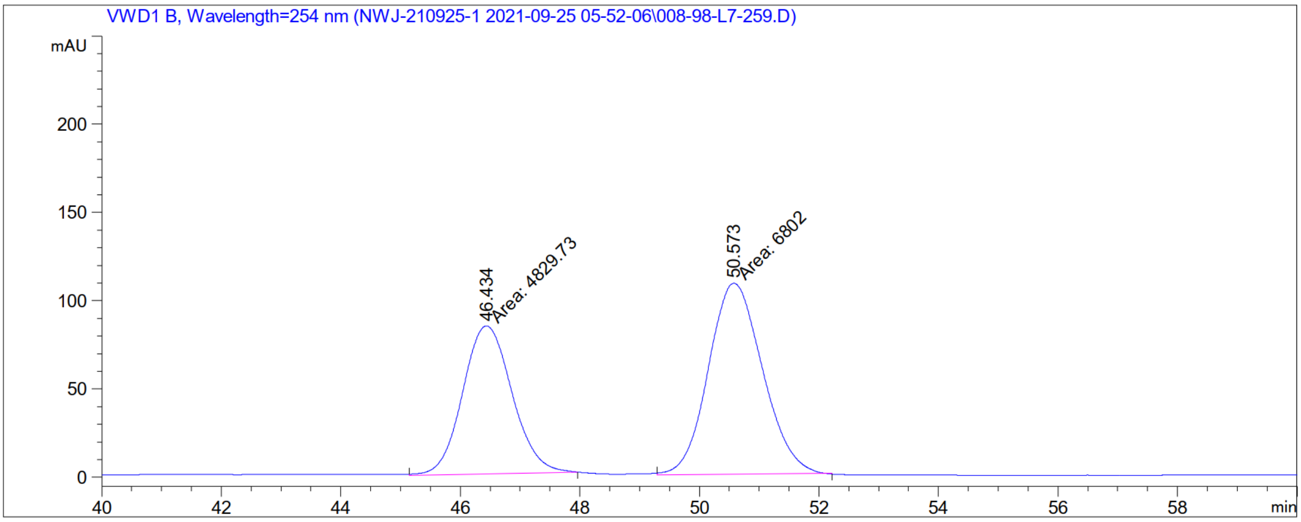

Signal 1: VWD1 B, Wavelength=254 nm

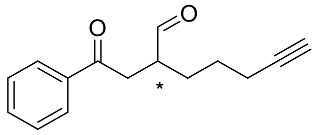

L2-5b-1

Peak #	RetTime [min]	Type	Width [min]	Area [mAU*s]	Height [mAU]	Area %
1	19.154	MM	0.4113	2205.75366	89.37522	45.2076
2	24.359	MM	0.5273	2673.40747	84.50108	54.7924
Totals :				4879.16113	173.87630	

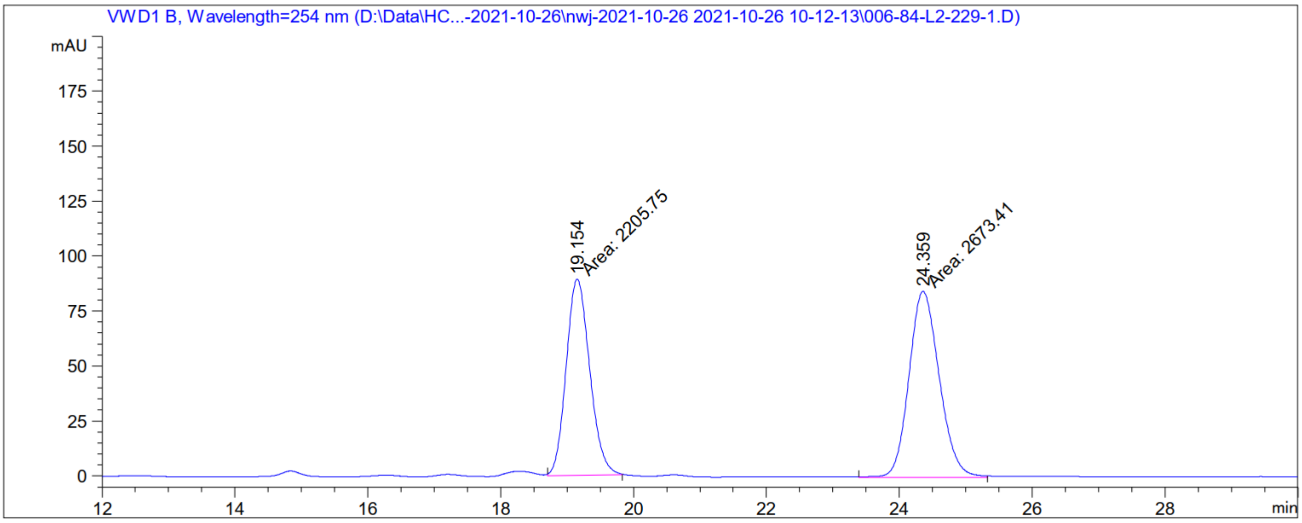

Signal 1: VWD1 B, Wavelength=254 nm

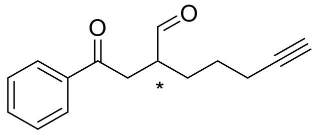

L2-5b-2

Peak #	RetTime [min]	Type	Width [min]	Area [mAU*s]	Height [mAU]	Area %
1	19.168	MM	0.4133	1863.02271	75.13192	43.4150
2	24.383	MM	0.5256	2428.16943	76.99593	56.5850
Totals :				4291.19214	152.12785	

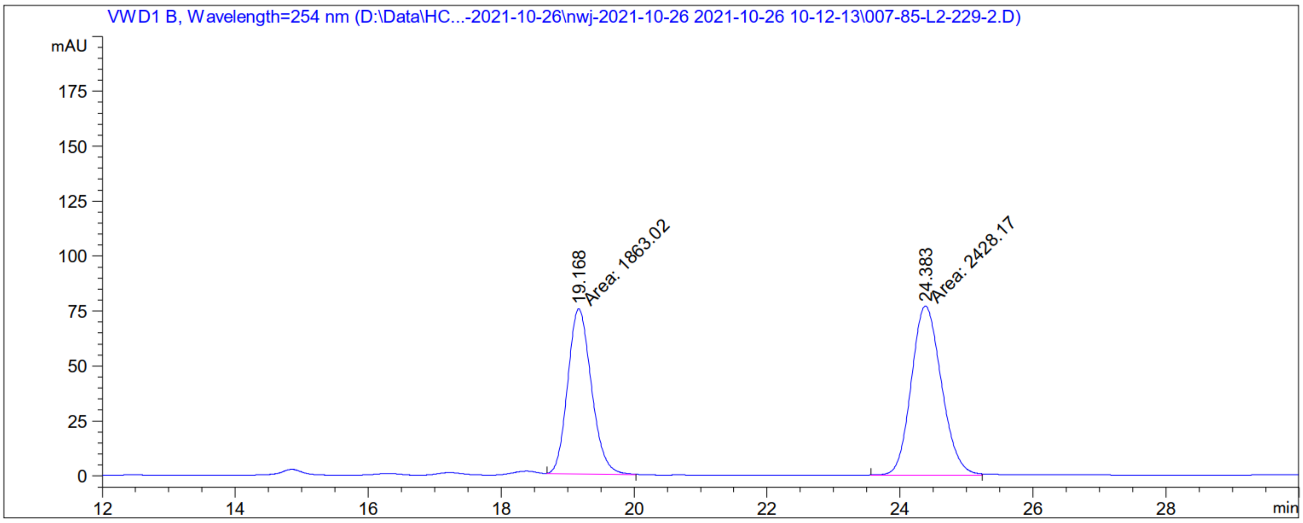

Signal 1: VWD1 B, Wavelength=254 nm

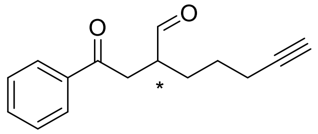

L2-5b-3

Peak #	RetTime [min]	Type	Width [min]	Area [mAU*s]	Height [mAU]	Area %
1	19.198	MM	0.4100	959.97247	39.02415	38.2549
2	24.409	MM	0.5230	1549.44006	49.37812	61.7451
Totals :				2509.41254	88.40228	

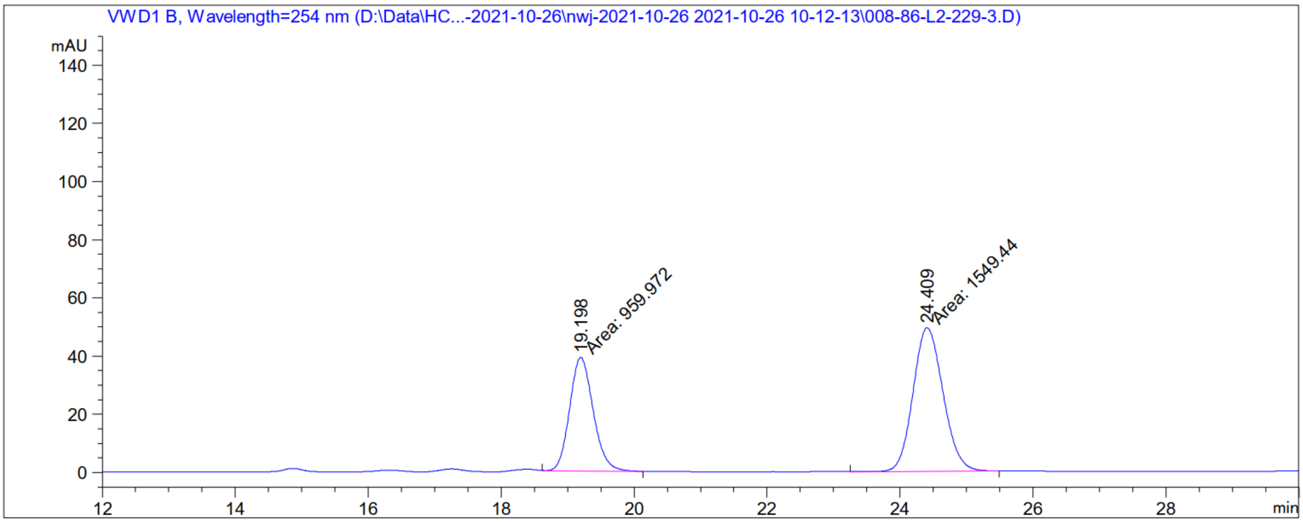

Signal 1: VWD1 B, Wavelength=254 nm

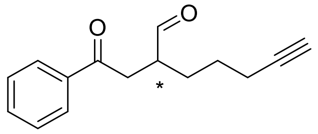

L2-5b-4

Peak #	RetTime [min]	Type	Width [min]	Area [mAU*s]	Height [mAU]	Area %
1	19.510	MM	0.4113	633.89301	25.68637	36.0034
2	24.706	BB	0.4756	1126.75452	36.75682	63.9966
Totals :				1760.64752	62.44319	

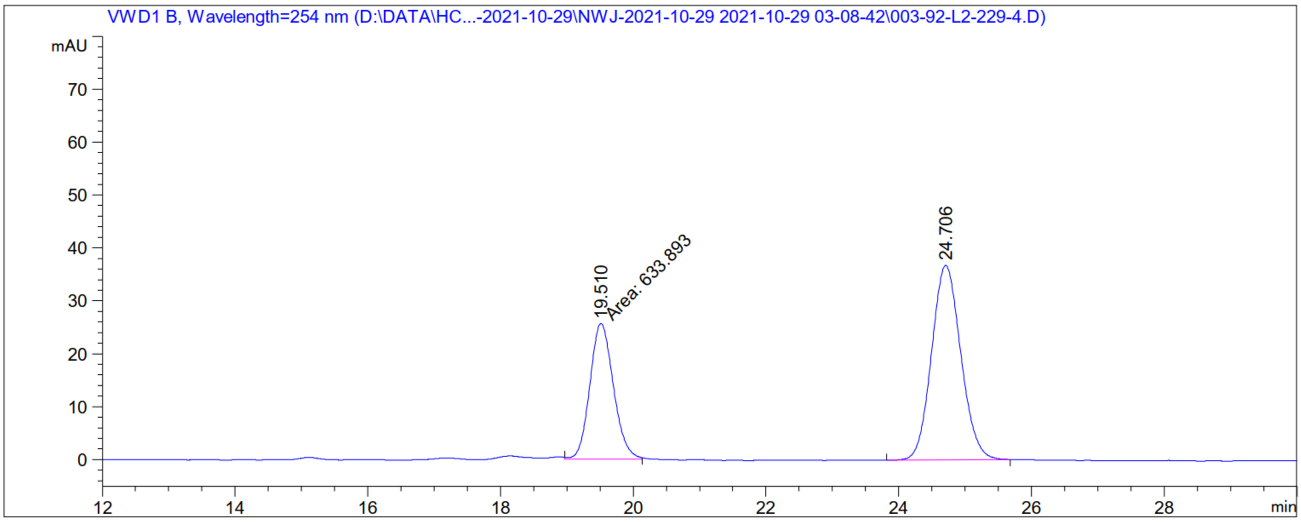

Signal 1: VWD1 B, Wavelength=254 nm

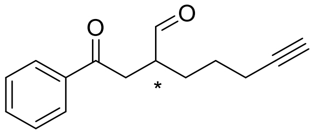

L2-5b-5

Peak #	RetTime [min]	Type	Width [min]	Area [mAU*s]	Height [mAU]	Area %
1	19.388	MM	0.3995	1169.93518	48.80291	34.4853
2	24.530	MM	0.5148	2222.63037	71.95550	65.5147
Totals :				3392.56555	120.75840	

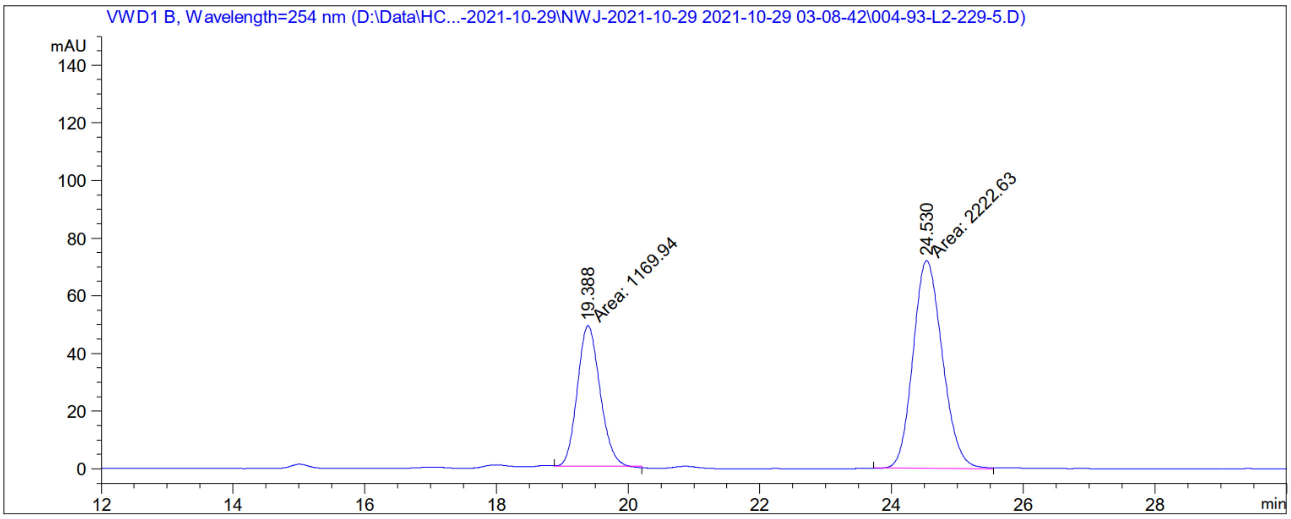

Signal 1: VWD1 B, Wavelength=254 nm

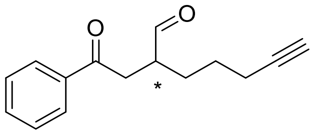

L2-5b-6

Peak #	RetTime [min]	Type	Width [min]	Area [mAU*s]	Height [mAU]	Area %
1	19.389	MM	0.4082	909.89380	37.14894	36.6829
2	24.535	MM	0.5109	1570.53955	51.23894	63.3171
Totals :				2480.43335	88.38787	

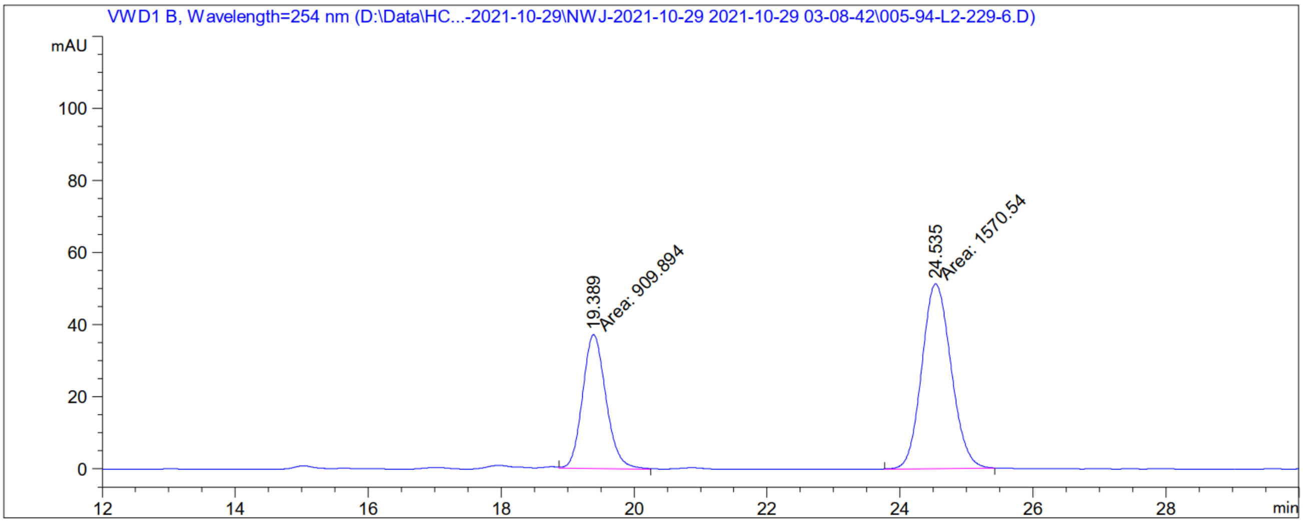

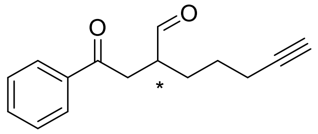

L3-5b-1

Signal 1: VWD1 B, Wavelength=254 nm

Peak #	RetTime [min]	Type	Width [min]	Area [mAU*s]	Height [mAU]	Area %
1	19.167	MM	0.4055	412.84268	16.96670	7.5695
2	24.312	MM	0.5239	5041.15381	160.38297	92.4305

Totals : 5453.99649 177.34967

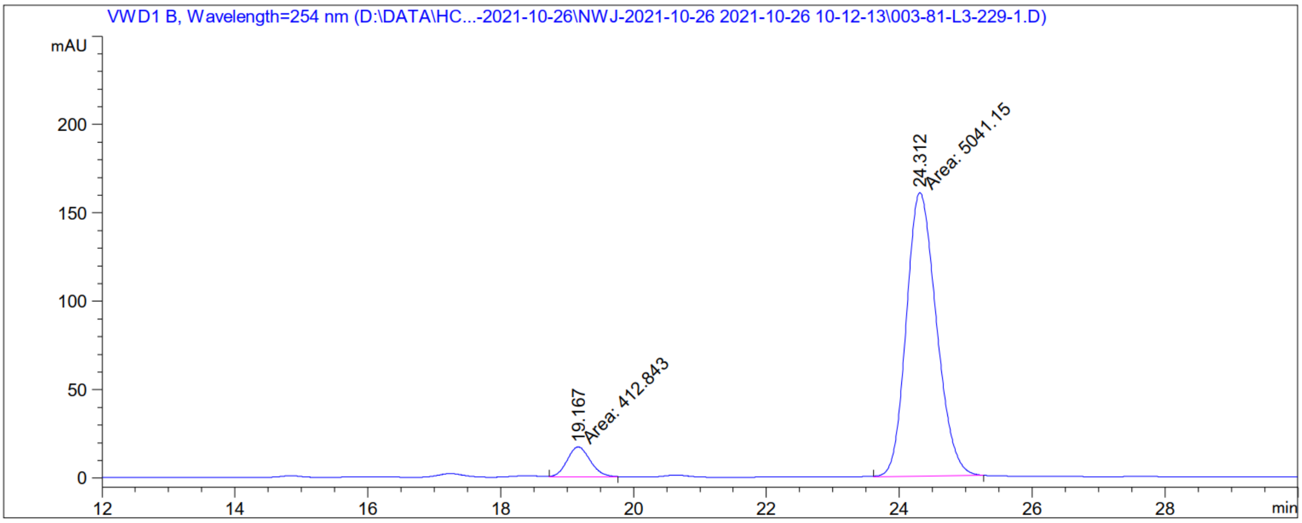

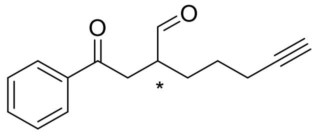

L3-5b-2

Signal 1: VWD1 B, Wavelength=254 nm

Peak #	RetTime [min]	Type	Width [min]	Area [mAU*s]	Height [mAU]	Area %
1	19.173	MM	0.4054	472.30164	19.41597	8.1076
2	24.315	MM	0.5336	5353.14795	167.19966	91.8924

Totals : 5825.44958 186.61563

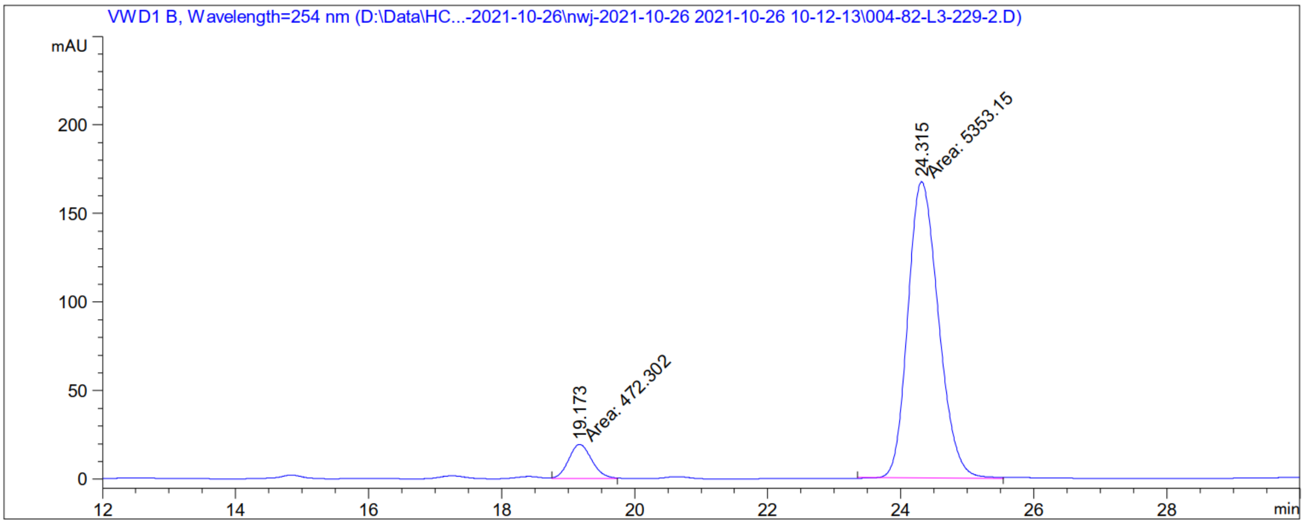

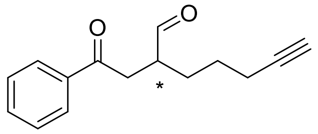

L3-5b-3

Signal 1: VWD1 B, Wavelength=254 nm

Peak #	RetTime [min]	Type	Width [min]	Area [mAU*s]	Height [mAU]	Area %
1	19.155	MM	0.3990	557.27625	23.28060	10.7544
2	24.309	MM	0.5422	4624.57959	142.15356	89.2456
Totals :				5181.85583	165.43416	

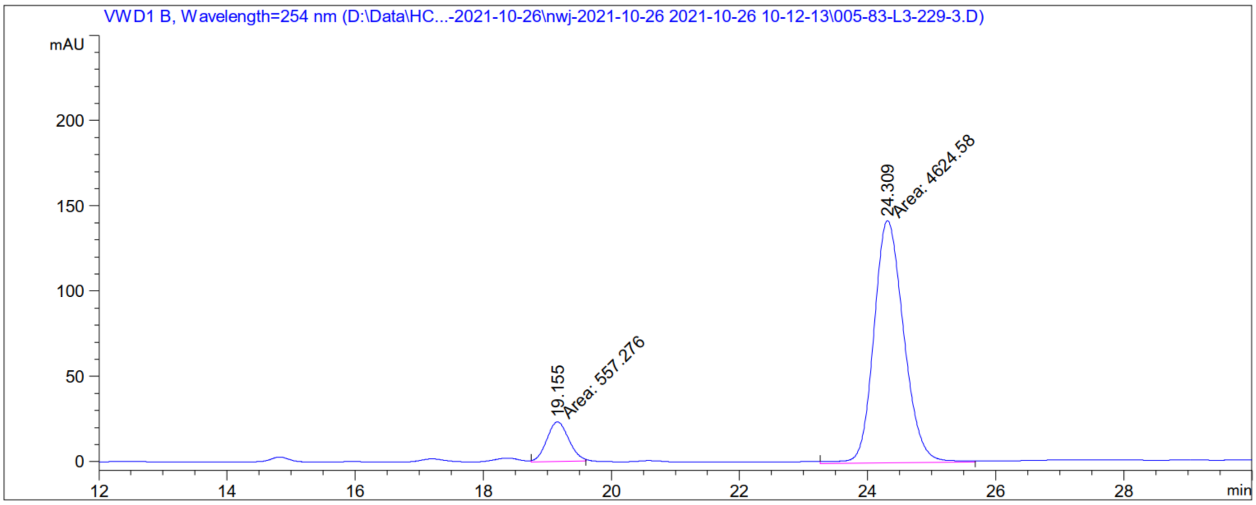

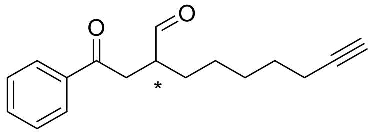

L1-5c

Signal 1: VWD1 B, Wavelength=254 nm

Peak #	RetTime [min]	Type	Width [min]	Area [mAU*s]	Height [mAU]	Area %
1	21.508	MM	0.4559	1979.57397	72.37373	50.3262
2	28.314	MM	0.6032	1953.90991	53.99047	49.6738
Totals :				3933.48389	126.36420	

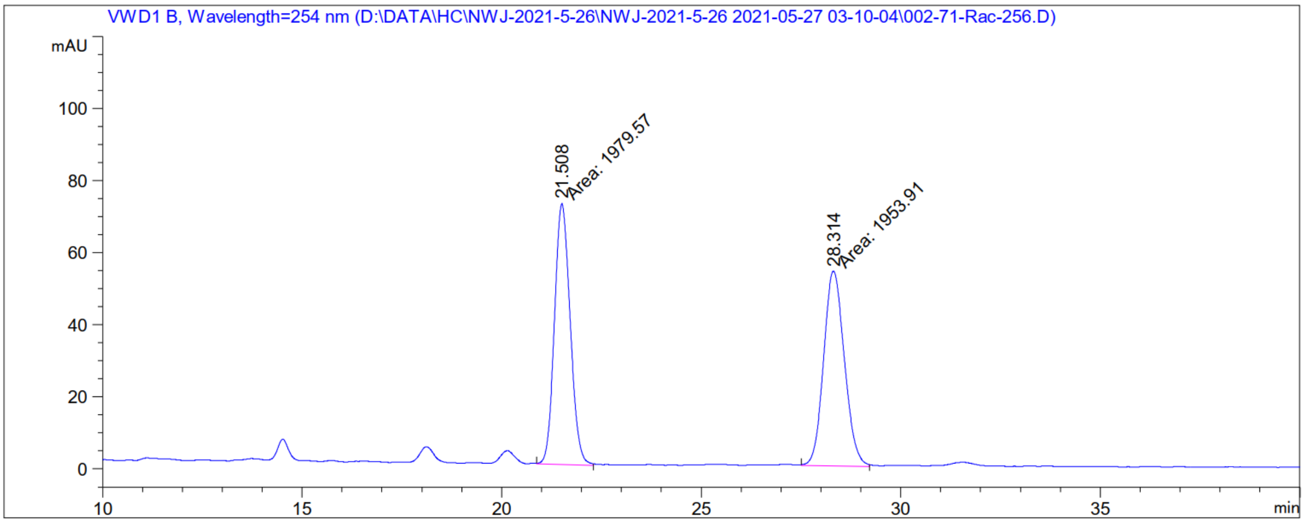

Signal 1: VWD1 B, Wavelength=254 nm

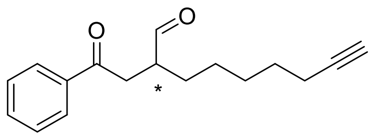

L2-5c

Peak #	RetTime [min]	Type	Width [min]	Area [mAU*s]	Height [mAU]	Area %
1	21.478	BB	0.4238	1226.22473	45.01035	34.7539
2	28.271	BB	0.5690	2302.08057	63.18792	65.2461
Totals :				3528.30530	108.19827	

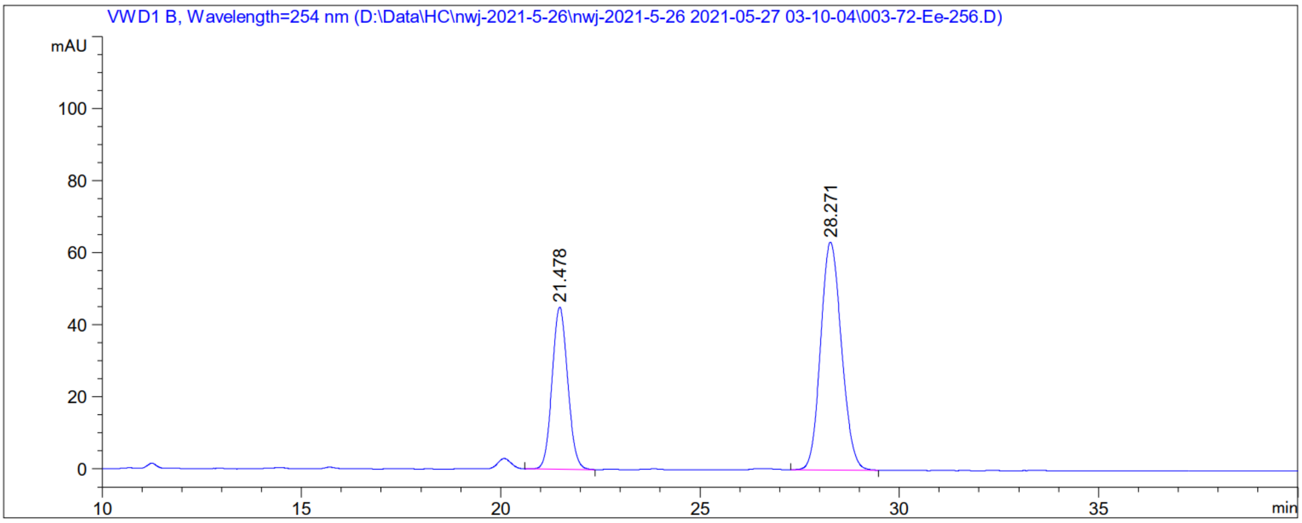

Signal 1: VWD1 B, Wavelength=254 nm

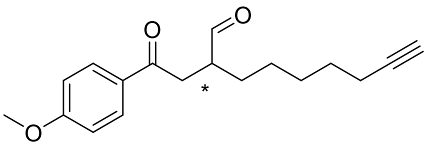

L1-5e

Peak #	RetTime [min]	Type	Width [min]	Area [mAU*s]	Height [mAU]	Area %
1	38.315	BB	0.8154	7397.99072	143.52214	50.0520
2	42.848	BB	0.8940	7382.62402	126.66630	49.9480
Totals :				1.47806e4	270.18844	

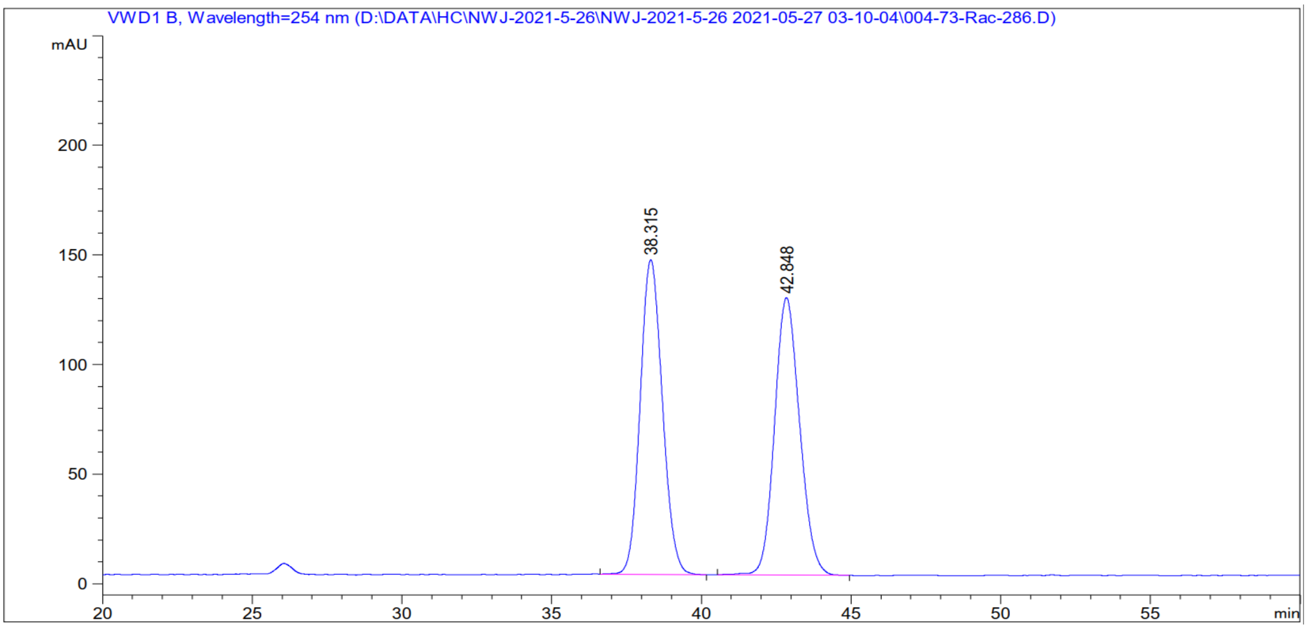

Signal 1: VWD1 B, Wavelength=254 nm

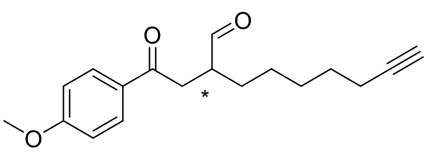

Peak #	RetTime [min]	Type	Width [min]	Area [mAU*s]	Height [mAU]	Area %
1	38.059	MM	0.8389	2853.45459	56.69153	29.3938
2	42.484	MM	0.9506	6854.21826	120.17277	70.6062
Totals :				9707.67285	176.86430	

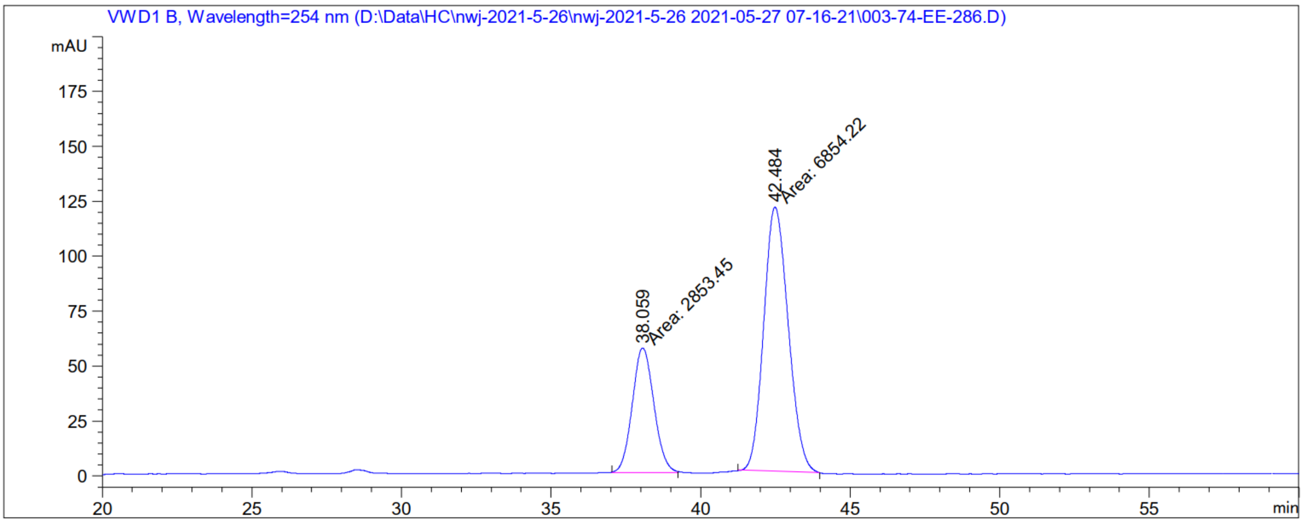

Signal 1: VWD1 B, Wavelength=254 nm

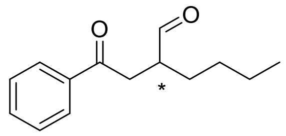

Peak #	RetTime [min]	Type	Width [min]	Area [mAU*s]	Height [mAU]	Area %
1	13.944	MM	0.2980	1971.41431	110.24115	35.7407
2	18.755	MM	0.4128	3544.46558	143.09004	64.2593
Totals :				5515.87988	253.33119	

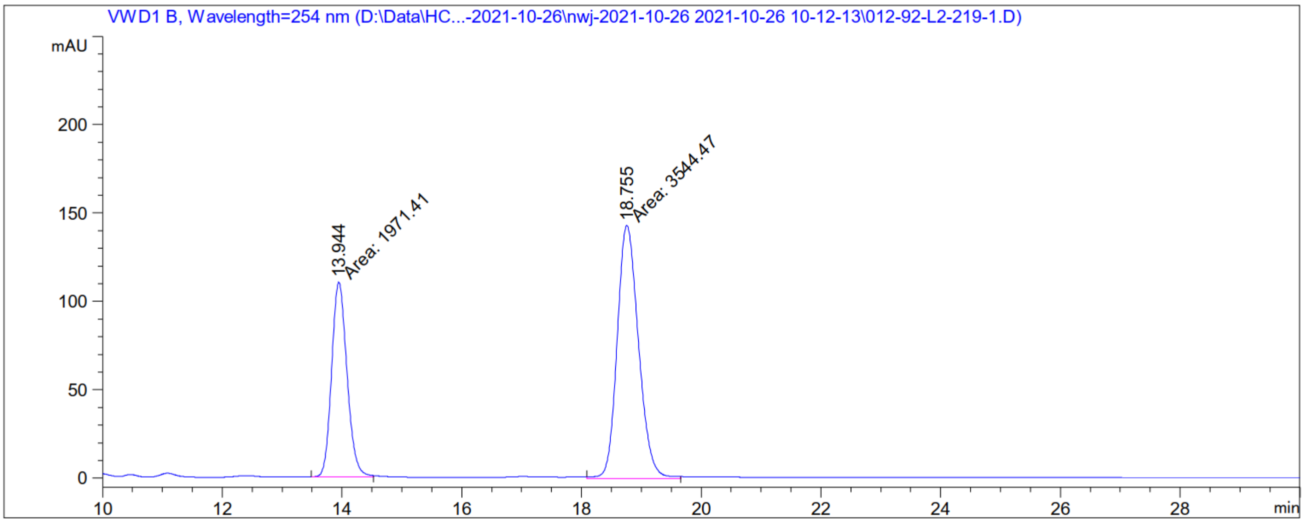

Signal 1: VWD1 B, Wavelength=254 nm

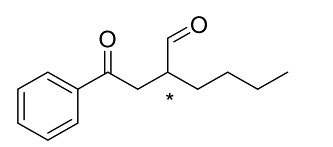

L2-5a-2

Peak #	RetTime [min]	Type	Width [min]	Area [mAU*s]	Height [mAU]	Area %
1	13.964	BB	0.2819	1243.99402	68.48808	33.5866
2	18.780	BB	0.3769	2459.84570	101.45307	66.4134
Totals :				3703.83972	169.94115	

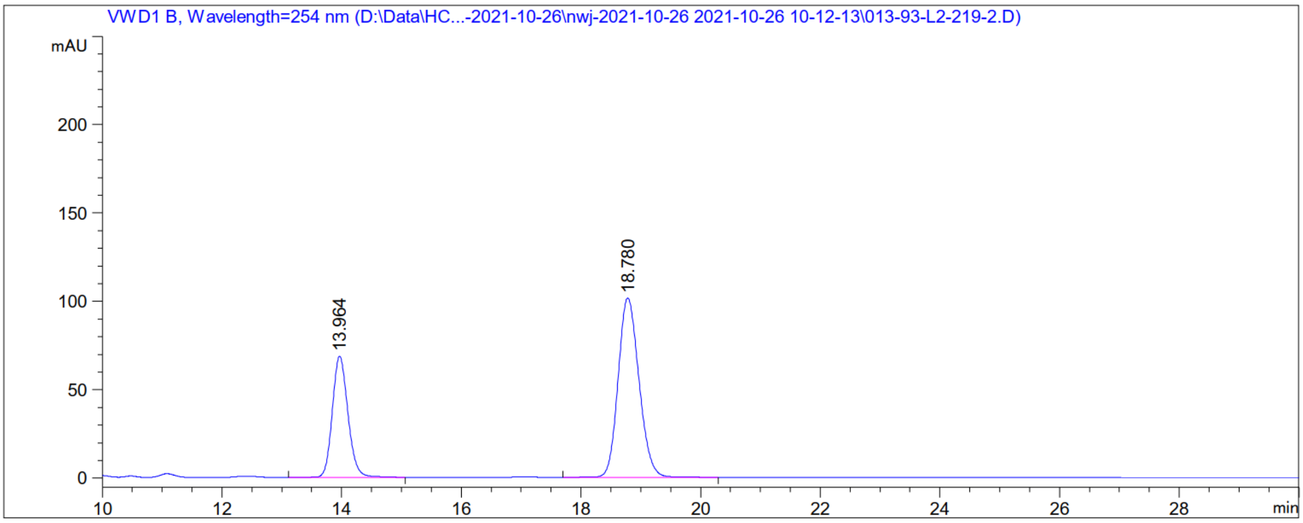

Signal 1: VWD1 B, Wavelength=254 nm

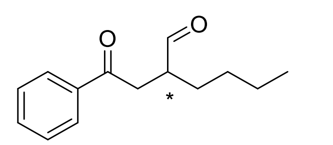

L2-5a-3

Peak #	RetTime [min]	Type	Width [min]	Area [mAU*s]	Height [mAU]	Area %
1	13.947	MM	0.3058	1370.20349	74.67972	31.0022
2	18.772	MM	0.4124	3049.49780	123.23063	68.9978
Totals :				4419.70129	197.91035	

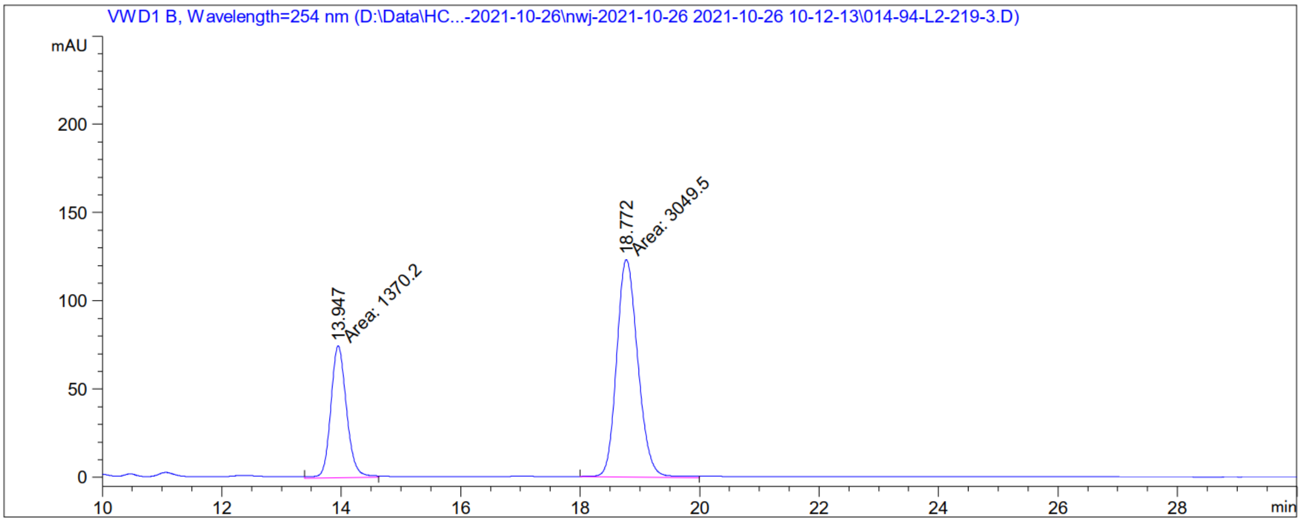

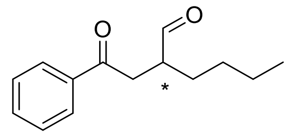

L3-5a-1

Signal 1: VWD1 B, Wavelength=254 nm

Peak #	RetTime [min]	Type	Width [min]	Area [mAU*s]	Height [mAU]	Area %
1	13.973	MM	0.2933	628.37695	35.70458	12.2327
2	18.747	MM	0.4132	4508.48047	181.83612	87.7673
Totals :				5136.85742	217.54070	

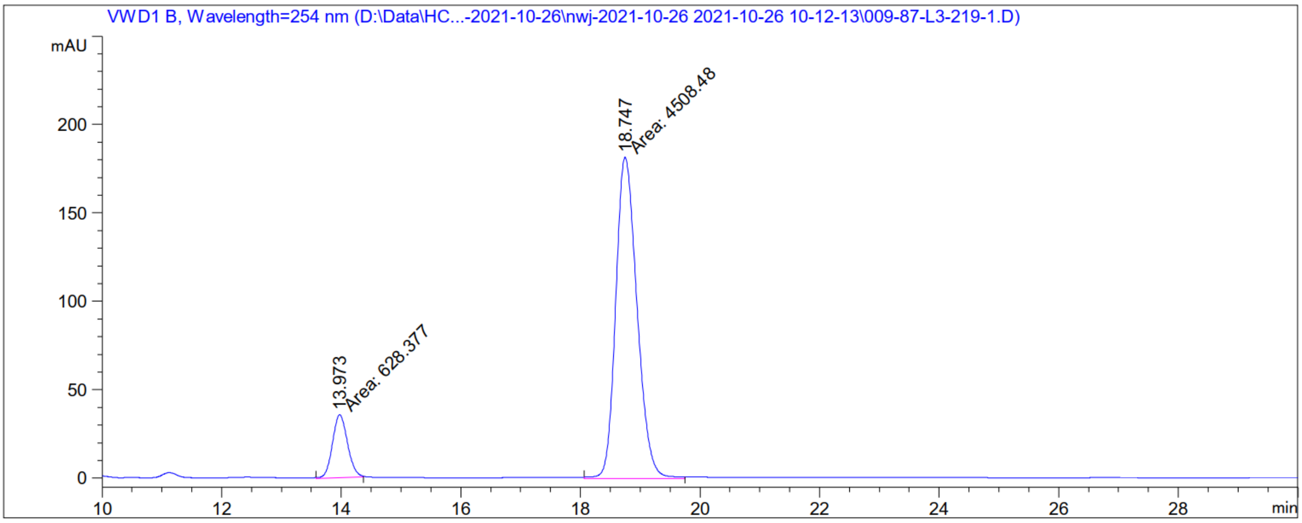

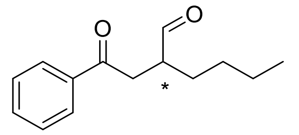

L3-5a-2

Signal 1: VWD1 B, Wavelength=254 nm

Peak #	RetTime [min]	Type	Width [min]	Area [mAU*s]	Height [mAU]	Area %
1	13.966	MM	0.2975	560.56232	31.40457	12.9352
2	18.758	MM	0.4057	3773.05151	154.98940	87.0648
Totals :				4333.61383	186.39397	

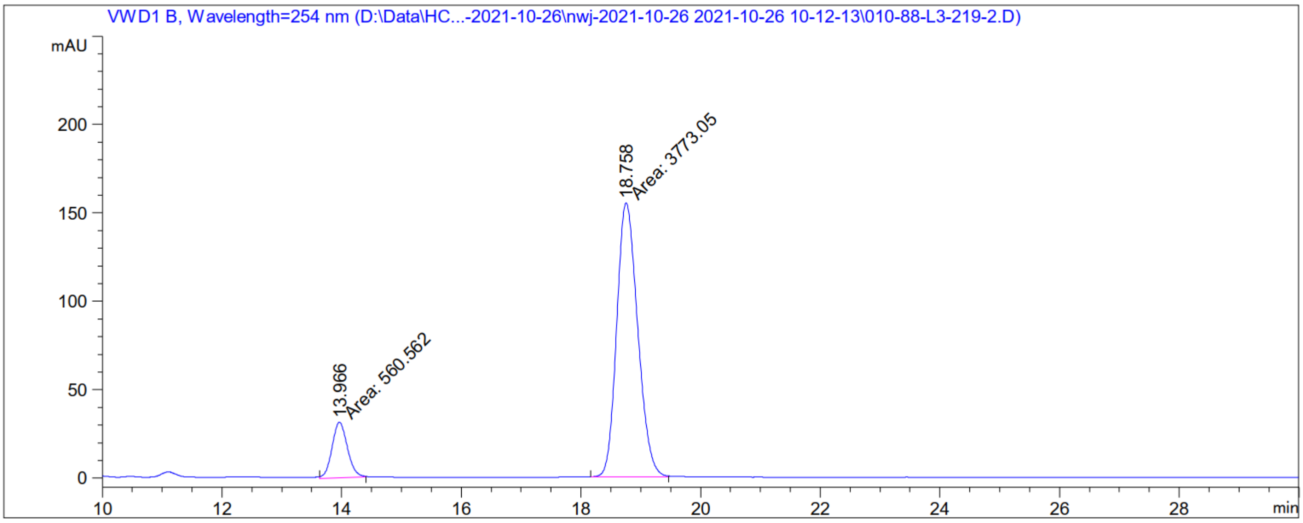

Signal 1: VWD1 B, Wavelength=254 nm

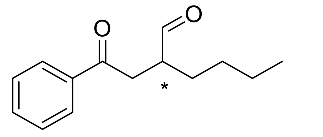

L3-5a-3

Peak #	RetTime [min]	Type	Width [min]	Area [mAU*s]	Height [mAU]	Area %
1	13.955	MM	0.2907	511.83881	29.34357	10.3444
2	18.732	MM	0.4119	4436.15576	179.47960	89.6556

Totals : 4947.99457 208.82317

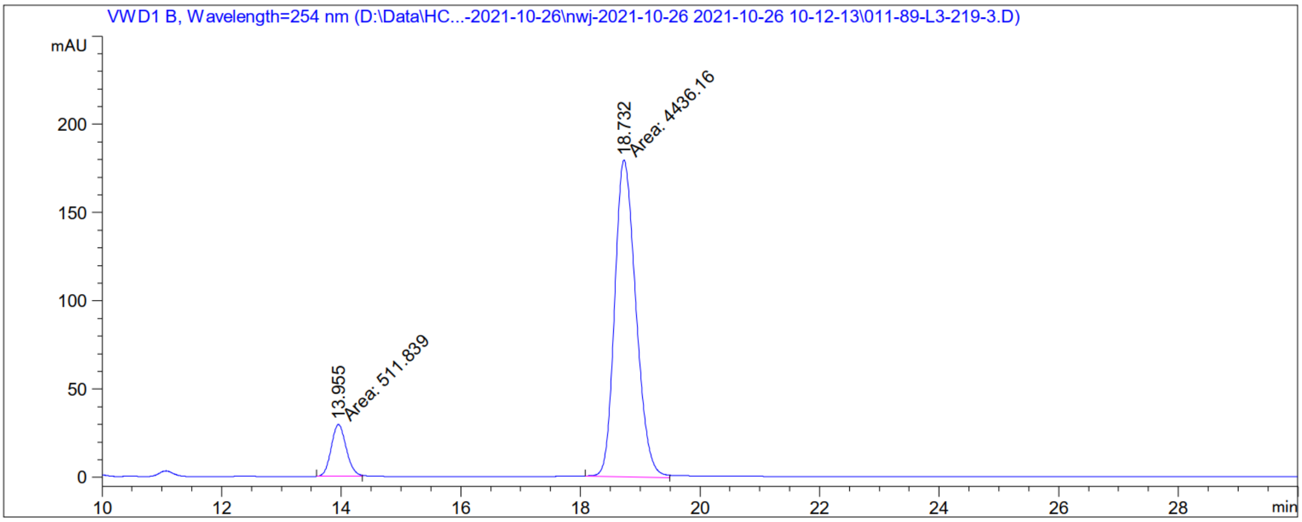

Signal 1: VWD1 A, Wavelength=220 nm

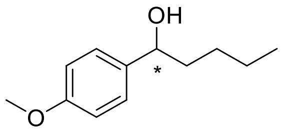

$NaBH_4$-7a

Peak #	RetTime [min]	Type	Width [min]	Area [mAU*s]	Height [mAU]	Area %
1	28.811	BB	0.5877	1.67151e4	443.47180	49.9487
2	32.741	BB	0.6741	1.67494e4	389.19589	50.0513

Totals : 3.34645e4 832.66769

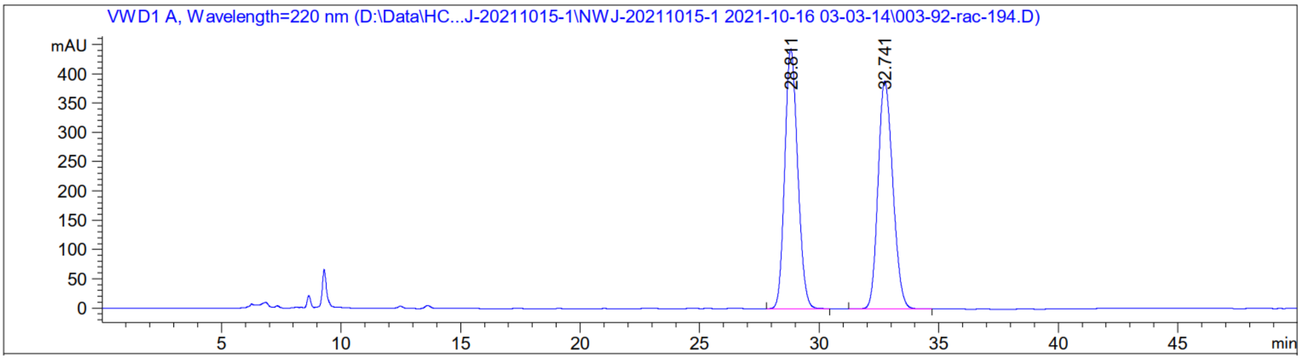

Signal 1: VWD1 A, Wavelength=220 nm

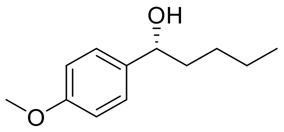

***R*-7a-1**

Peak #	RetTime [min]	Type	Width [min]	Area [mAU*s]	Height [mAU]	Area %
1	33.273	MM	0.7704	2.34462e4	507.23337	84.4112
2	37.880	BB	0.8306	4329.96875	81.66711	15.5888

Totals : 2.77762e4 588.90047

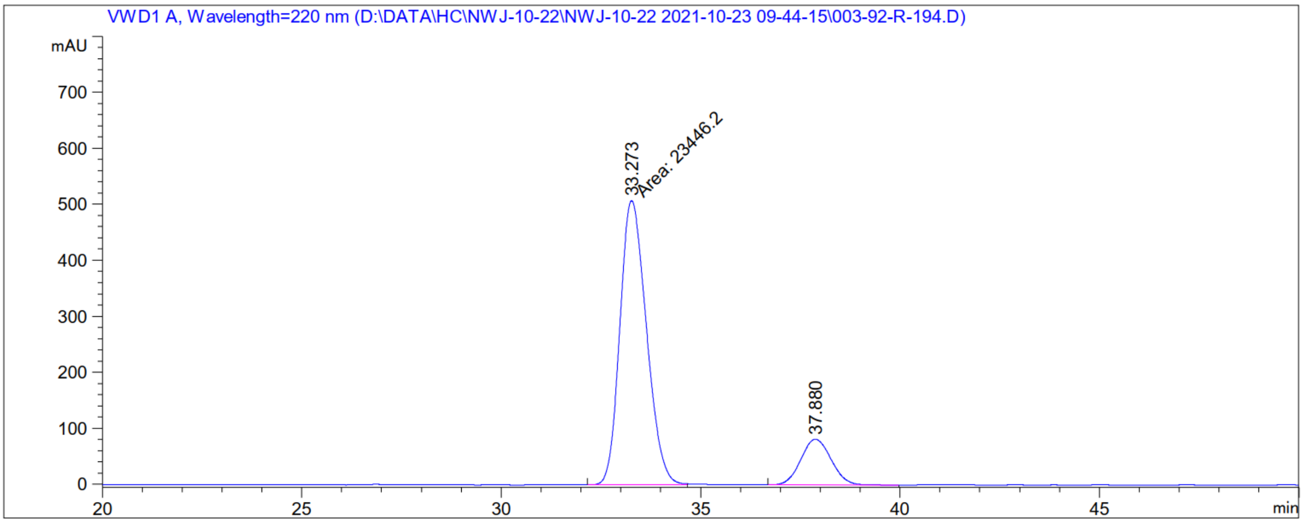

Signal 1: VWD1 A, Wavelength=220 nm

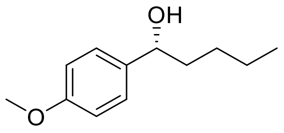

***R*-7a-2**

Peak #	RetTime [min]	Type	Width [min]	Area [mAU*s]	Height [mAU]	Area %
1	35.861	MM	0.7762	2.28358e4	490.31732	86.3715
2	40.314	MM	0.8504	3603.25879	70.62299	13.6285

Totals : 2.64391e4 560.94032

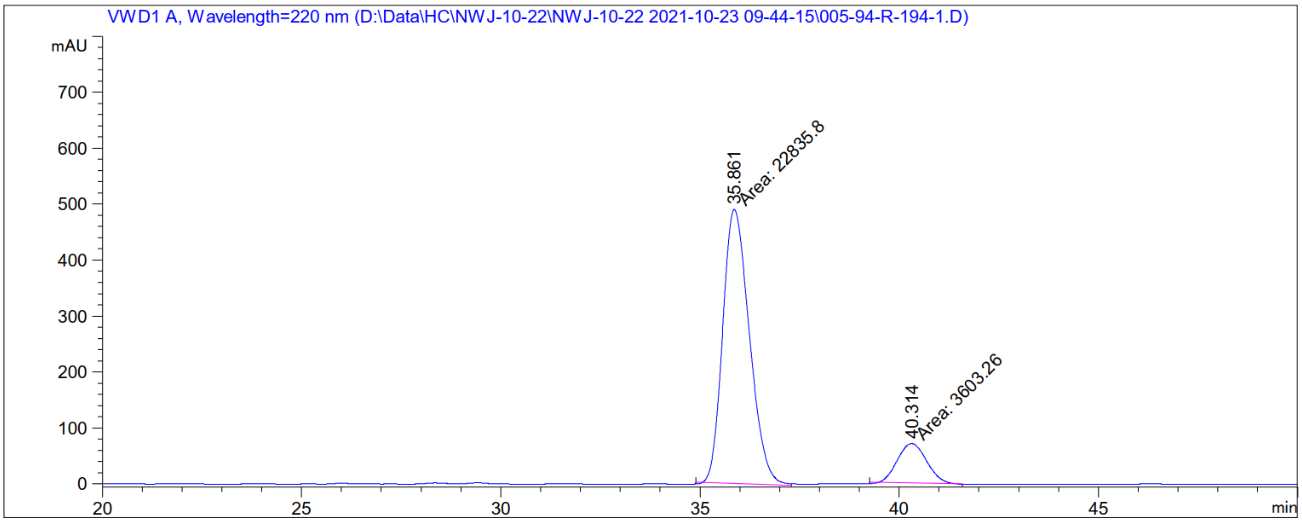

Signal 1: VWD1 A, Wavelength=220 nm

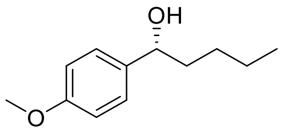

***R*-7a-3**

Peak #	RetTime [min]	Type	Width [min]	Area [mAU*s]	Height [mAU]	Area %
1	35.441	BB	0.7148	3.11654e4	677.34991	86.5711
2	39.734	BB	0.8092	4834.35742	93.50957	13.4289

Totals : 3.59997e4 770.85948

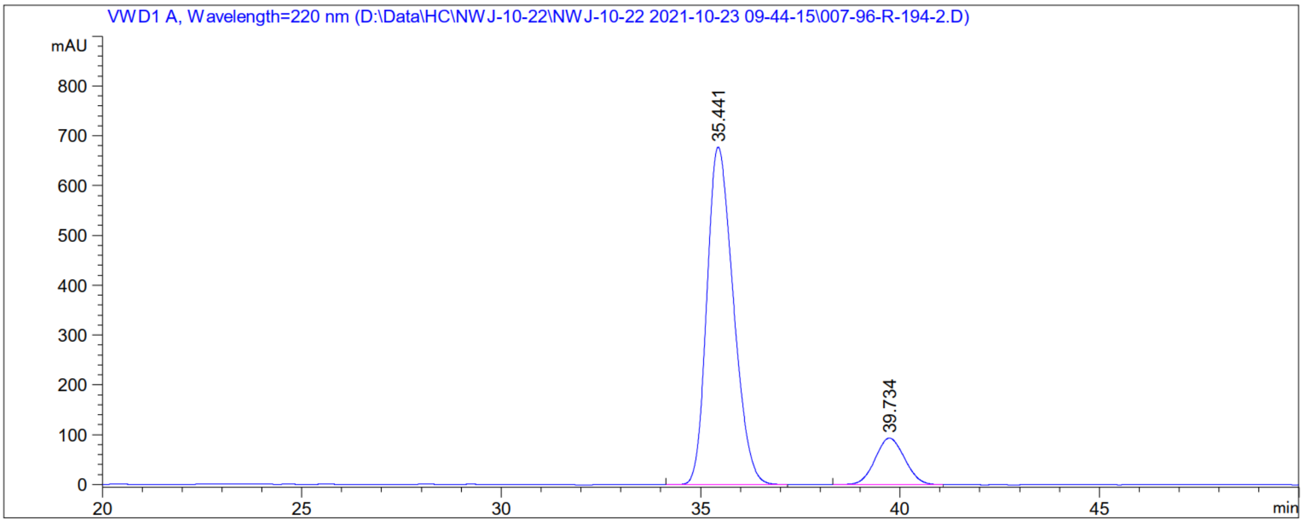

Signal 1: VWD1 A, Wavelength=220 nm

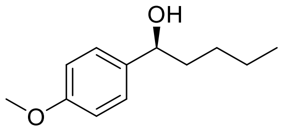

***S*-7a-1**

Peak #	RetTime [min]	Type	Width [min]	Area [mAU*s]	Height [mAU]	Area %
1	36.537	MM	0.7976	2679.13281	55.98618	12.2072
2	40.983	MM	0.8977	1.92680e4	357.71936	87.7928

Totals : 2.19472e4 413.70554

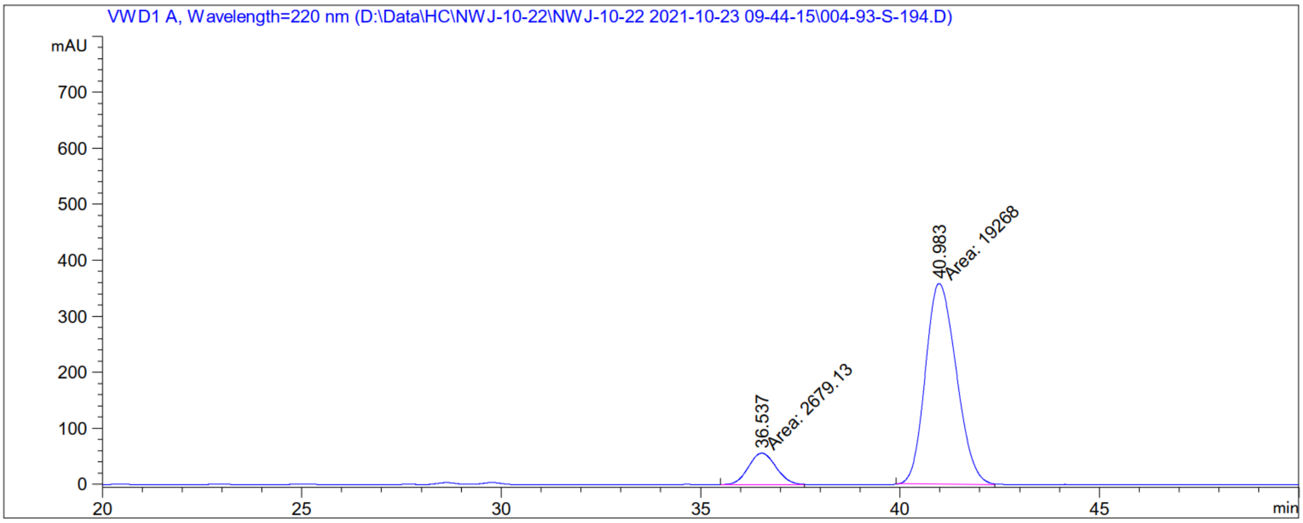

Signal 1: VWD1 A, Wavelength=220 nm

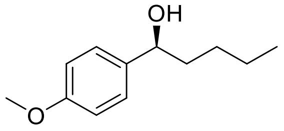

S-7a-2

Peak #	RetTime [min]	Type	Width [min]	Area [mAU*s]	Height [mAU]	Area %
1	36.024	MM	0.7911	1960.00366	41.29391	13.2587
2	40.166	VB R	0.8094	1.28228e4	247.92252	86.7413

Totals : 1.47828e4 289.21643

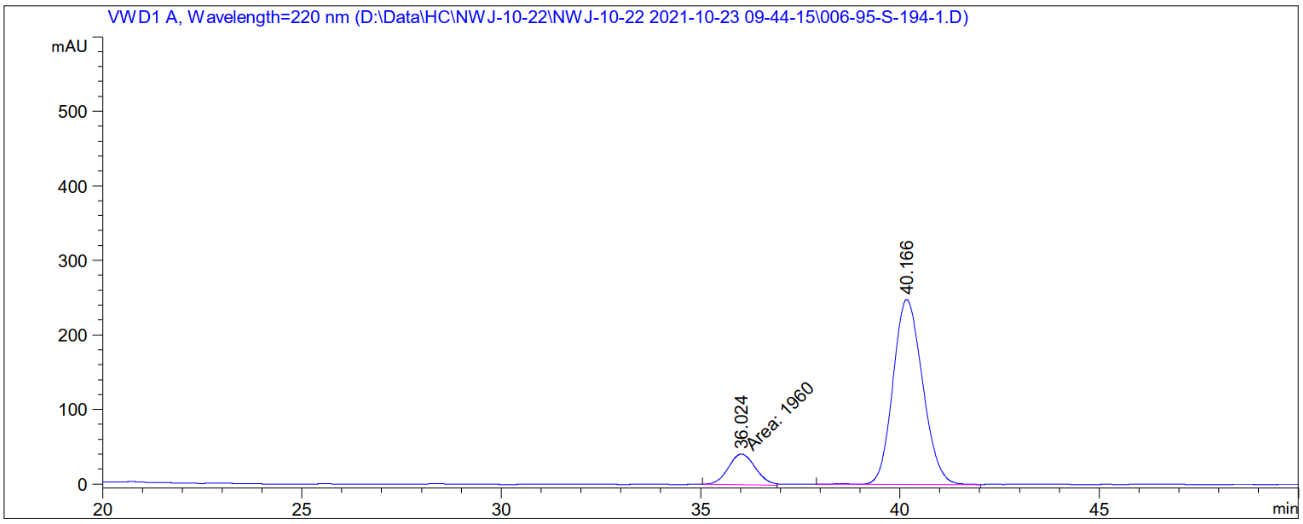

Signal 1: VWD1 A, Wavelength=220 nm

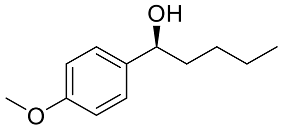

S-7a-3

Peak #	RetTime [min]	Type	Width [min]	Area [mAU*s]	Height [mAU]	Area %
1	34.040	BB	0.6699	4212.28809	98.30958	12.6875
2	37.772	BB	0.7892	2.89880e4	579.87543	87.3125

Totals : 3.32003e4 678.18501

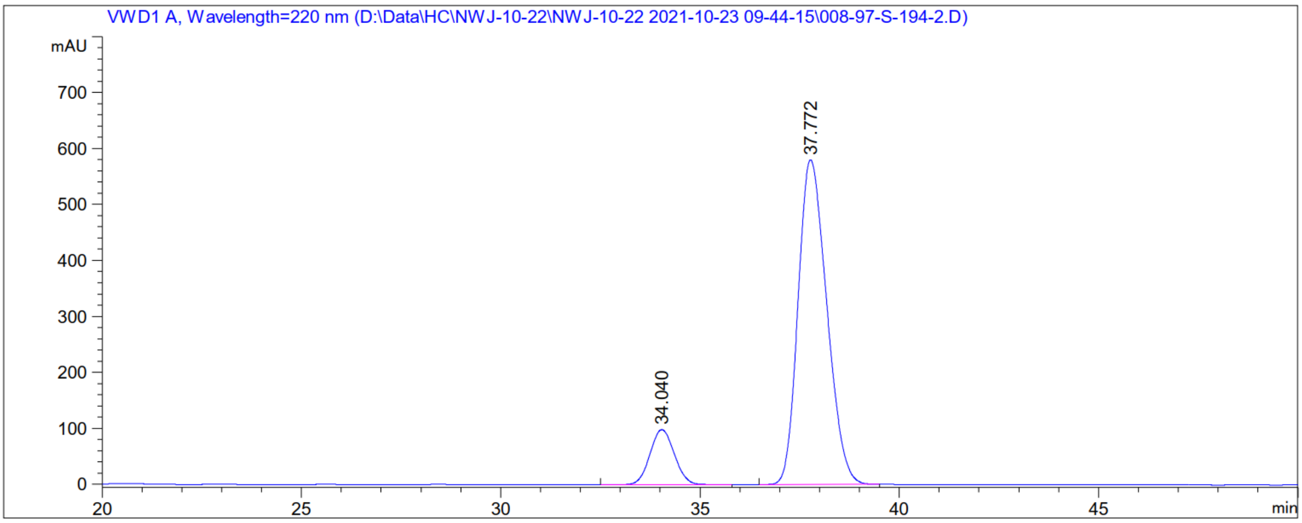

Signal 1: VWD1 A, Wavelength=220 nm

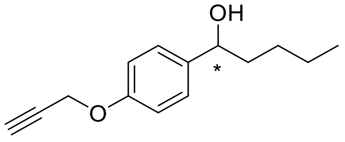

NaBH$_4$-7b

Peak #	RetTime [min]	Type	Width [min]	Area [mAU*s]	Height [mAU]	Area %
1	9.376	MM	0.2407	1.17214e4	811.59882	49.7599
2	10.891	MM	0.2791	1.18345e4	706.72467	50.2401

Totals : 2.35560e4 1518.32349

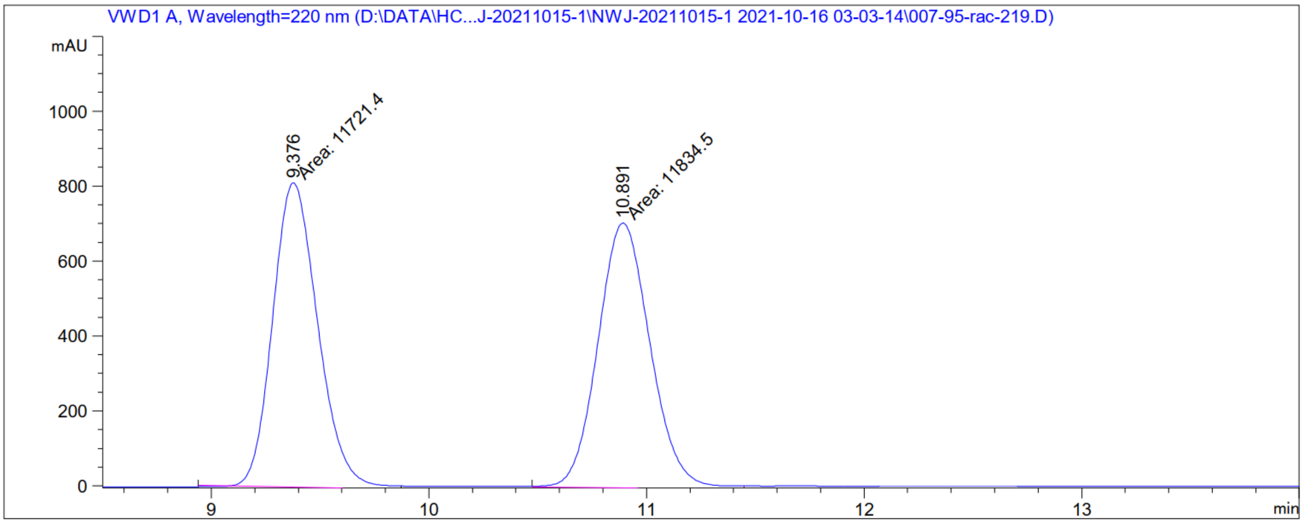

Signal 1: VWD1 A, Wavelength=220 nm

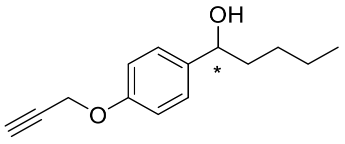

NaBH$_4$-7b-1

Peak #	RetTime [min]	Type	Width [min]	Area [mAU*s]	Height [mAU]	Area %
1	9.521	MM	0.2443	1.22141e4	833.39746	50.0278
2	11.024	MM	0.2753	1.22005e4	738.51471	49.9722

Totals : 2.44145e4 1571.91217

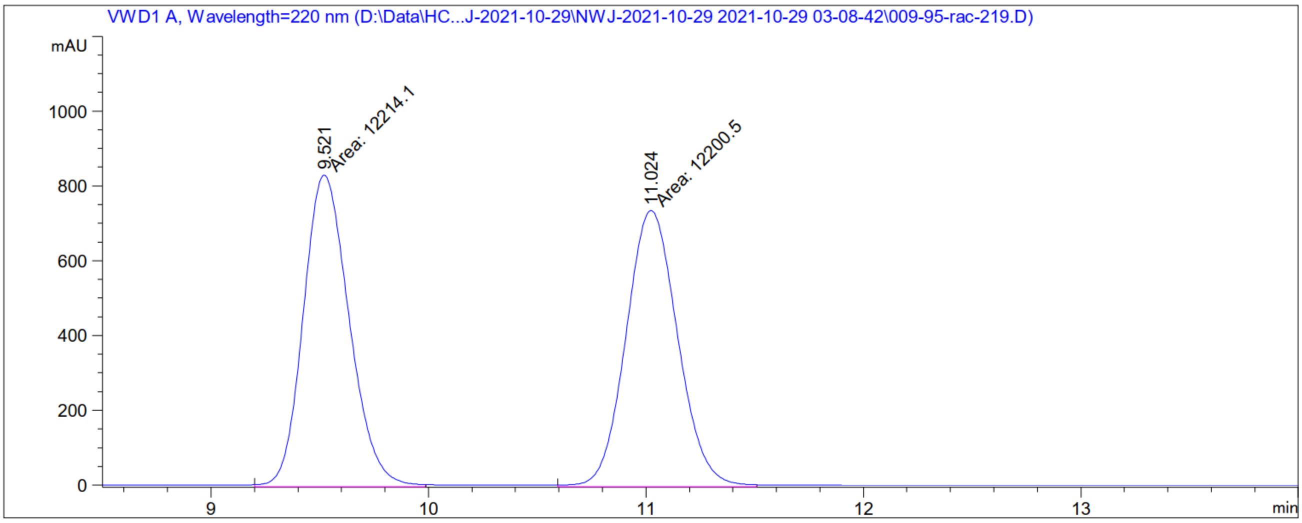

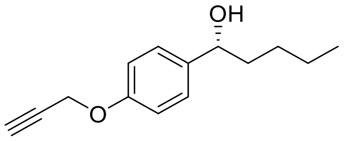

***R*-7b-1**

Signal 1: VWD1 A, Wavelength=220 nm

Peak #	RetTime [min]	Type	Width [min]	Area [mAU*s]	Height [mAU]	Area %
1	9.435	MM	0.2533	378.89575	24.92934	11.0835
2	10.922	MM	0.2738	3039.66064	185.02748	88.9165

Totals : 3418.55640 209.95682

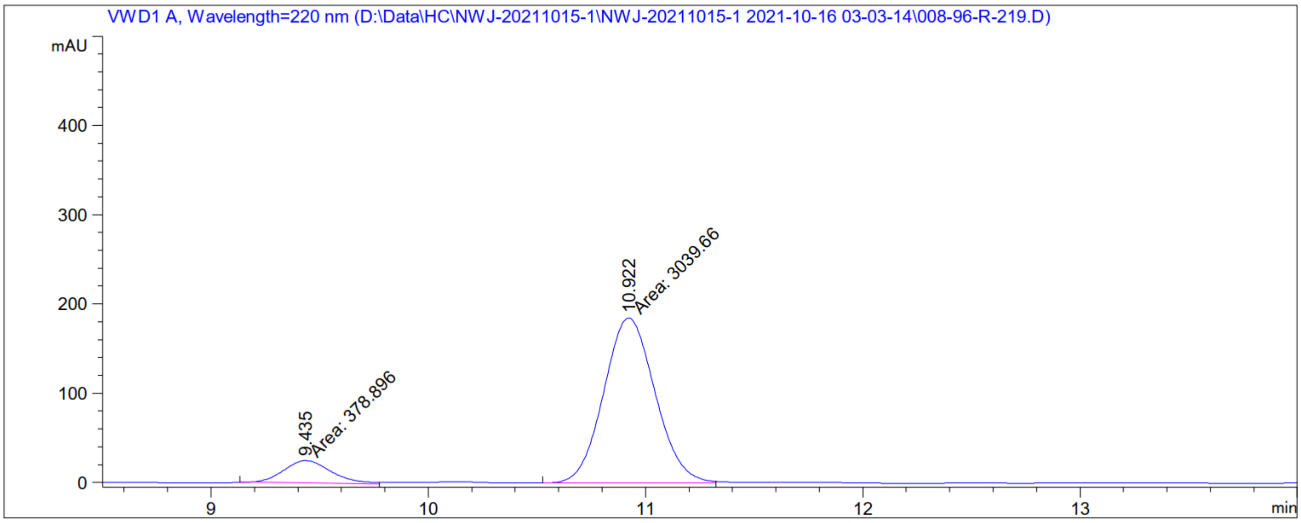

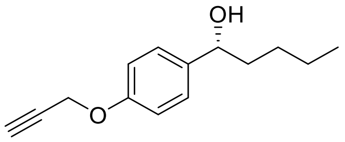

***R*-7b-2**

Signal 1: VWD1 A, Wavelength=220 nm

Peak #	RetTime [min]	Type	Width [min]	Area [mAU*s]	Height [mAU]	Area %
1	9.587	MM	0.2492	277.40857	18.55187	14.1760
2	11.074	MM	0.2725	1679.47900	102.73910	85.8240

Totals : 1956.88757 121.29097

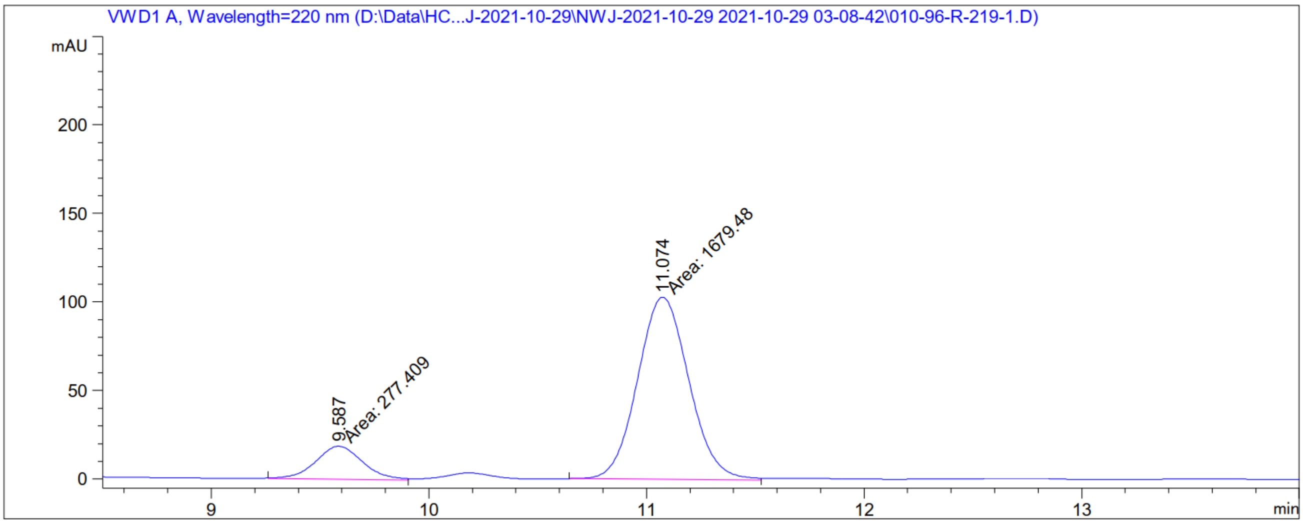

Signal 1: VWD1 A, Wavelength=220 nm

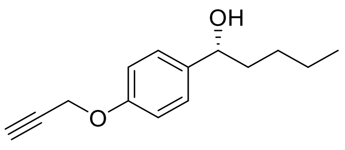

***R*-7b-3**

Peak #	RetTime [min]	Type	Width [min]	Area [mAU*s]	Height [mAU]	Area %
1	9.656	MM	0.2322	874.11658	62.74033	11.5866
2	11.156	MM	0.2684	6670.10791	414.24567	88.4134

Totals : 7544.22449 476.98599

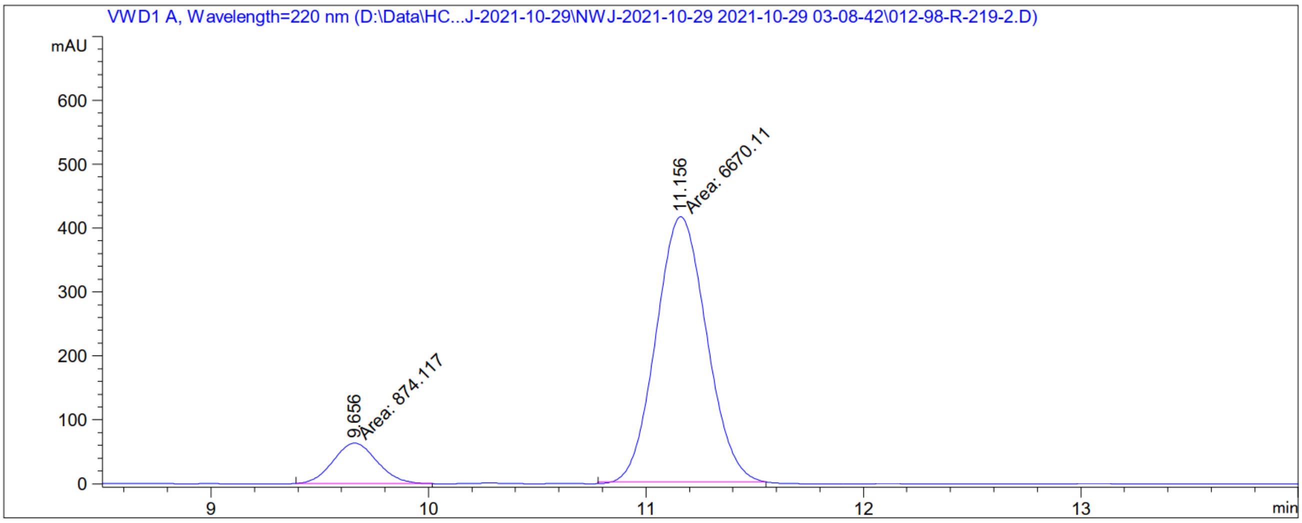

Signal 1: VWD1 A, Wavelength=220 nm

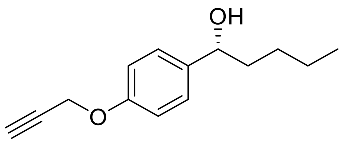

***S*-7b-1**

Peak #	RetTime [min]	Type	Width [min]	Area [mAU*s]	Height [mAU]	Area %
1	9.432	BB	0.2216	6997.41504	487.06616	91.2768
2	10.945	MM	0.2608	668.73108	42.73080	8.7232

Totals : 7666.14612 529.79696

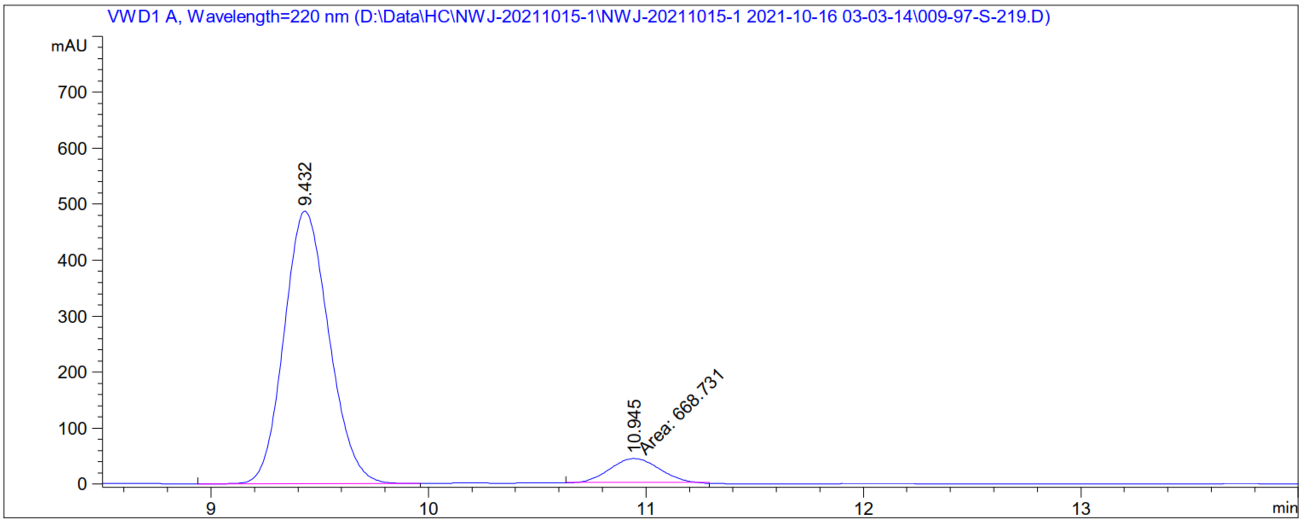

Signal 1: VWD1 A, Wavelength=220 nm

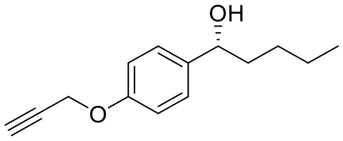

S-7b-2

Peak #	RetTime [min]	Type	Width [min]	Area [mAU*s]	Height [mAU]	Area %
1	9.246	MM	0.2379	1886.64685	132.19243	86.8505
2	10.734	MM	0.2936	285.64603	16.21618	13.1495

Totals : 2172.29288 148.40861

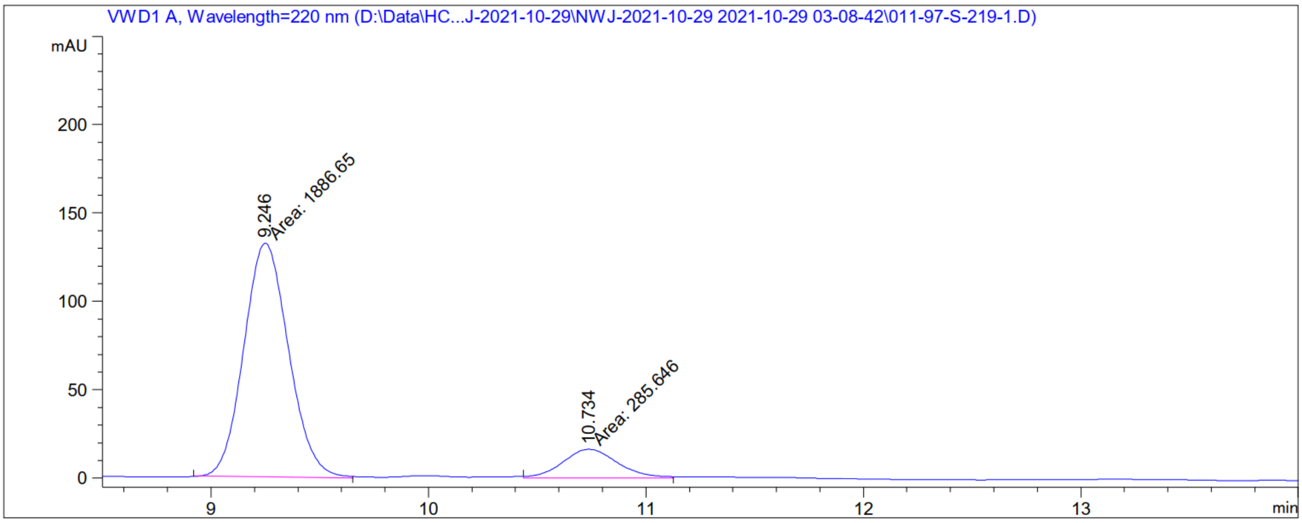

Signal 1: VWD1 A, Wavelength=220 nm

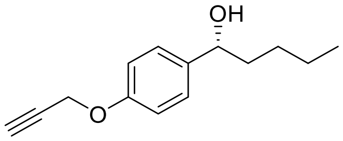

S-7b-3

Peak #	RetTime [min]	Type	Width [min]	Area [mAU*s]	Height [mAU]	Area %
1	9.683	MM	0.2353	5432.02637	384.77692	86.6683
2	11.218	MM	0.2824	835.58081	49.32256	13.3317

Totals : 6267.60718 434.09948

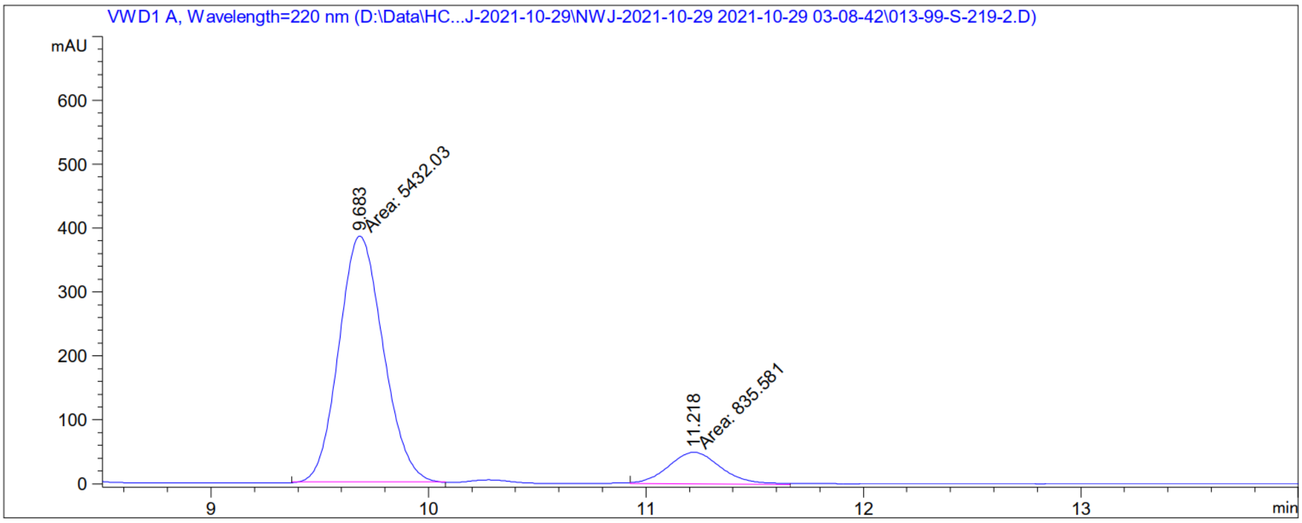

7. NMR spectra

^{1}H NMR (600 MHz, $CDCl_3$) δ 7.99 (d, *J* = 8.3 Hz, 2H), 7.74 (d, *J* = 7.6 Hz, 2H), 7.61–7.48 (m, 2H), 7.38 (t, *J* = 7.5 Hz, 2H), 7.31–7.26 (m, 2H), 7.00 (d, *J* = 8.3 Hz, 2H), 4.73 (d, *J* = 8.9 Hz, 1H), 4.47–4.36 (m, 2H), 4.35–4.24 (m, 2H), 4.15 (t, *J* = 6.0 Hz, 2H), 1.80–1.58 (m, 2H), 1.52–1.32 (m, 2H), 0.96 (dd, *J* = 6.6, 4.1 Hz, 6H).

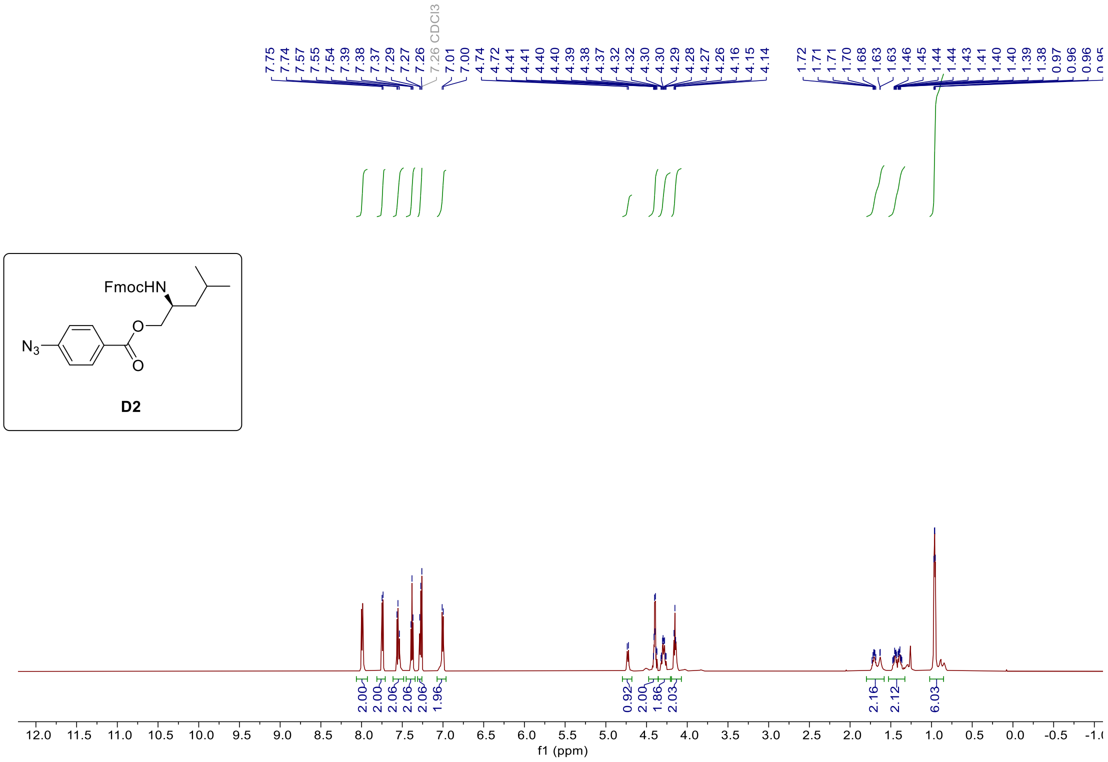

^{13}C NMR (151 MHz, $CDCl_3$) δ 165.83, 156.15, 145.08, 143.95, 141.44, 131.64, 127.81, 127.17, 126.48, 125.11, 120.10, 118.99, 67.24, 66.71, 48.84, 47.43, 40.98, 24.87, 23.22, 22.24.

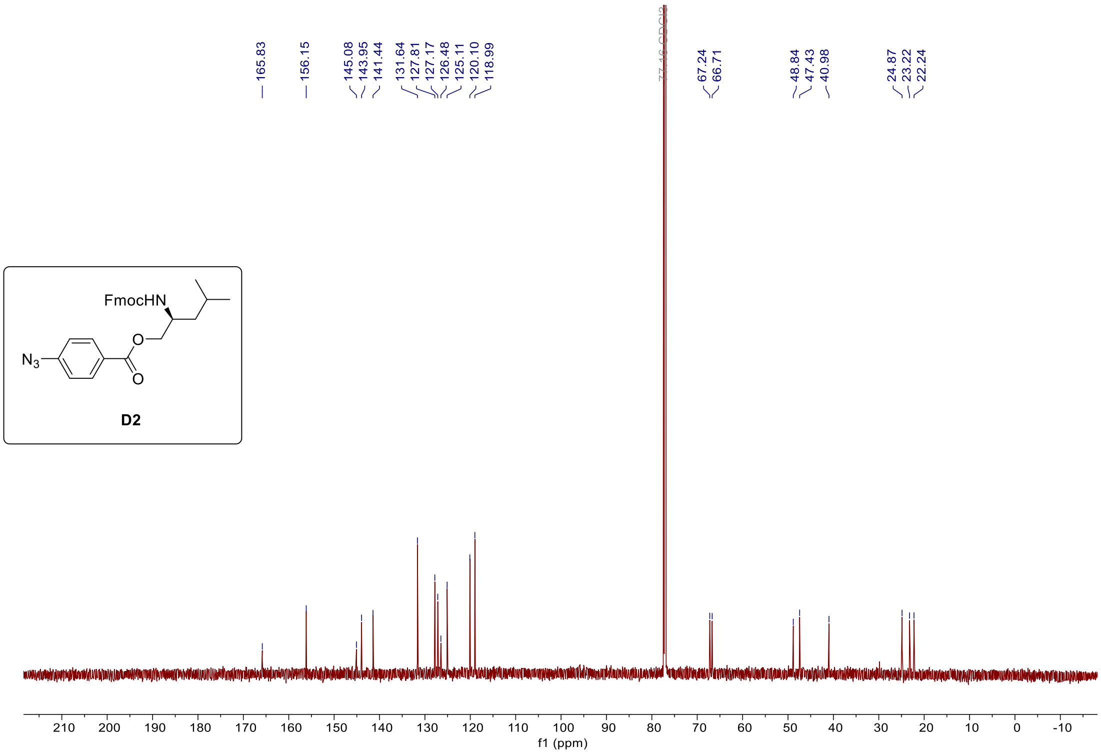

^{1}H NMR (600 MHz, $CDCl_3$) δ 8.01 (d, *J* = 8.3 Hz, 2H), 7.75 (dd, *J* = 7.6, 4.0 Hz, 2H), 7.52 (d, *J* = 7.5 Hz, 2H), 7.39 (t, *J* = 7.5 Hz, 2H), 7.35–7.16 (m, 7H), 7.04 (d, *J* = 8.3 Hz, 2H), 4.95 (d, *J* = 8.2 Hz, 1H), 4.42–4.26 (m, 4H), 4.17 (t, *J* = 6.9 Hz, 1H), 2.90–3.15 (m, 2H).

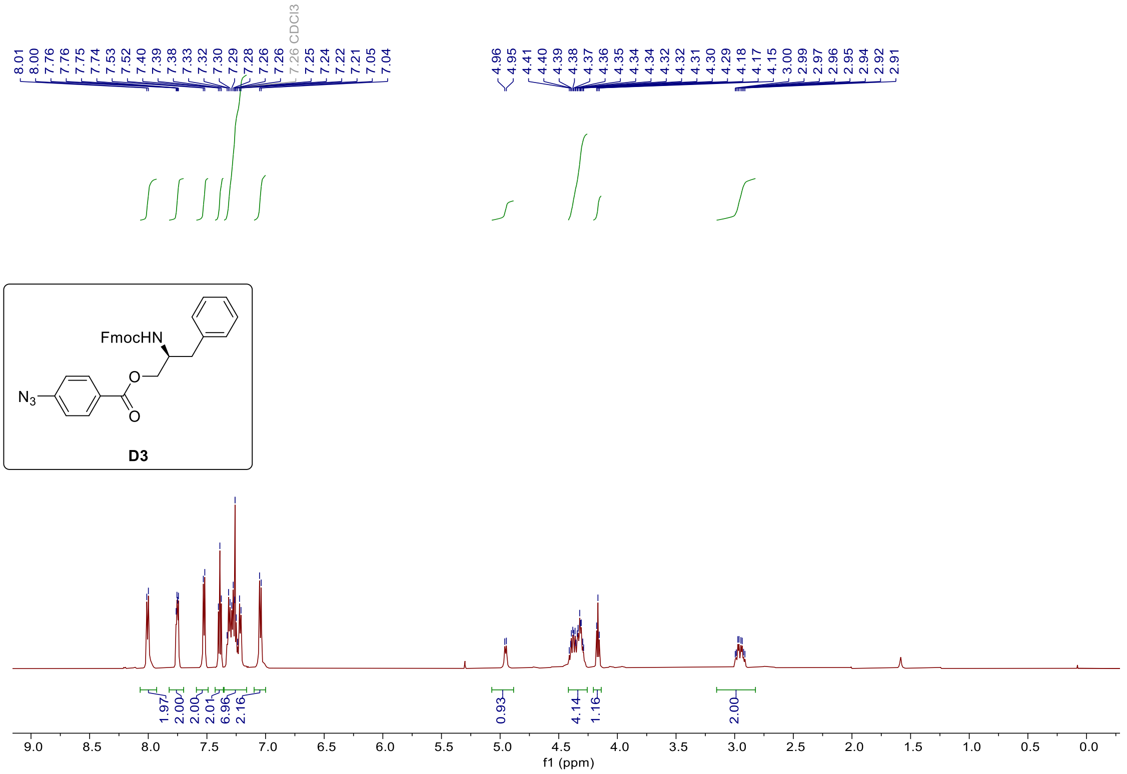

^{13}C NMR (151 MHz, $CDCl_3$) δ 165.76, 155.91, 145.21, 143.92, 141.40, 136.83, 131.65, 129.41, 128.88, 127.83, 127.15, 127.04, 126.27, 125.14, 120.11, 119.04, 66.85, 65.58, 51.53, 47.30, 38.05.

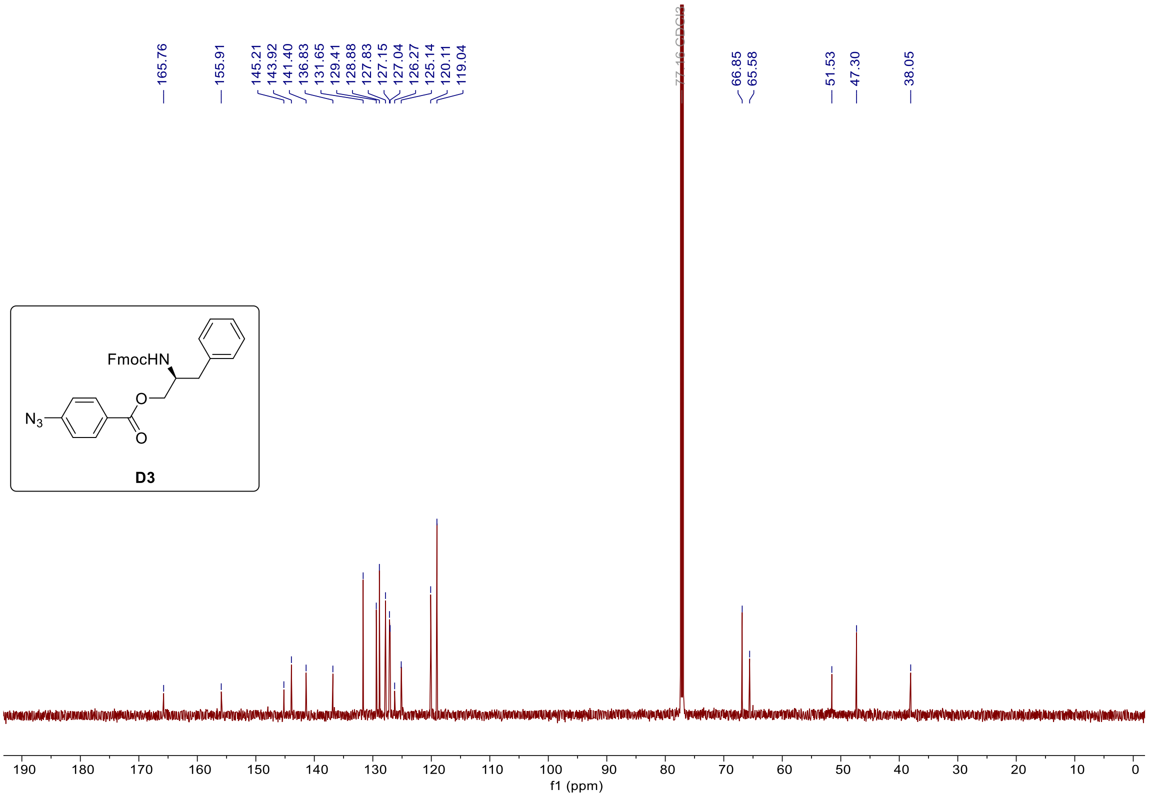

^{1}H NMR (600 MHz, $CDCl_3$) δ 8.04 (d, *J* = 8.9 Hz, 1H), 7.92 (d, *J* = 8.2 Hz, 1H), 7.76–7.67 (m, 2H), 7.54 (d, *J* = 8.6 Hz, 2H), 7.49–7.41 (m, 2H), 7.31–7.15 (m, 5H), 7.07 (dd, *J* = 8.2, 1.4 Hz, 1H), 6.77 (d, *J* = 8.6 Hz, 2H), 5.21 (s, 1H).

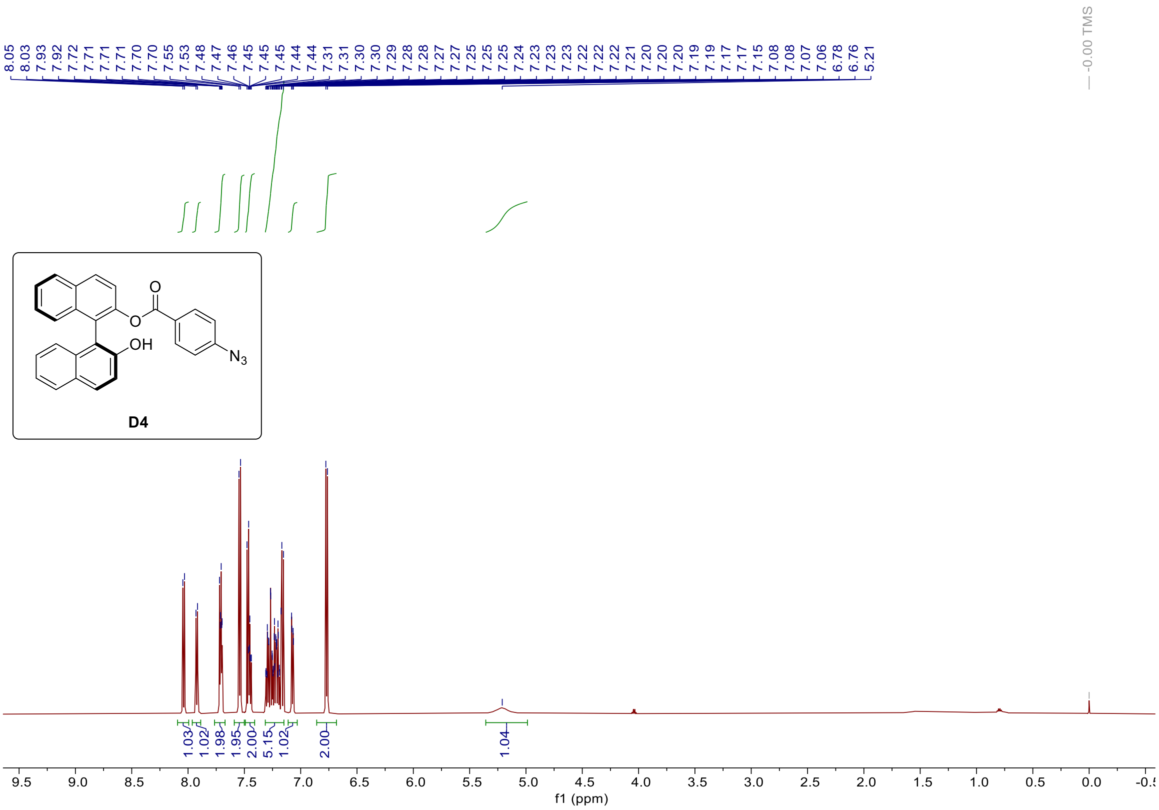

^{13}C NMR (151 MHz, $CDCl_3$) δ 165.13, 151.83, 148.34, 145.48, 133.61, 133.59, 132.42, 131.87, 130.99, 130.52, 129.08, 128.51, 128.15, 127.69, 126.84, 126.51, 125.89, 125.23, 124.68, 123.61, 123.19, 121.93, 118.87, 118.24, 113.97.

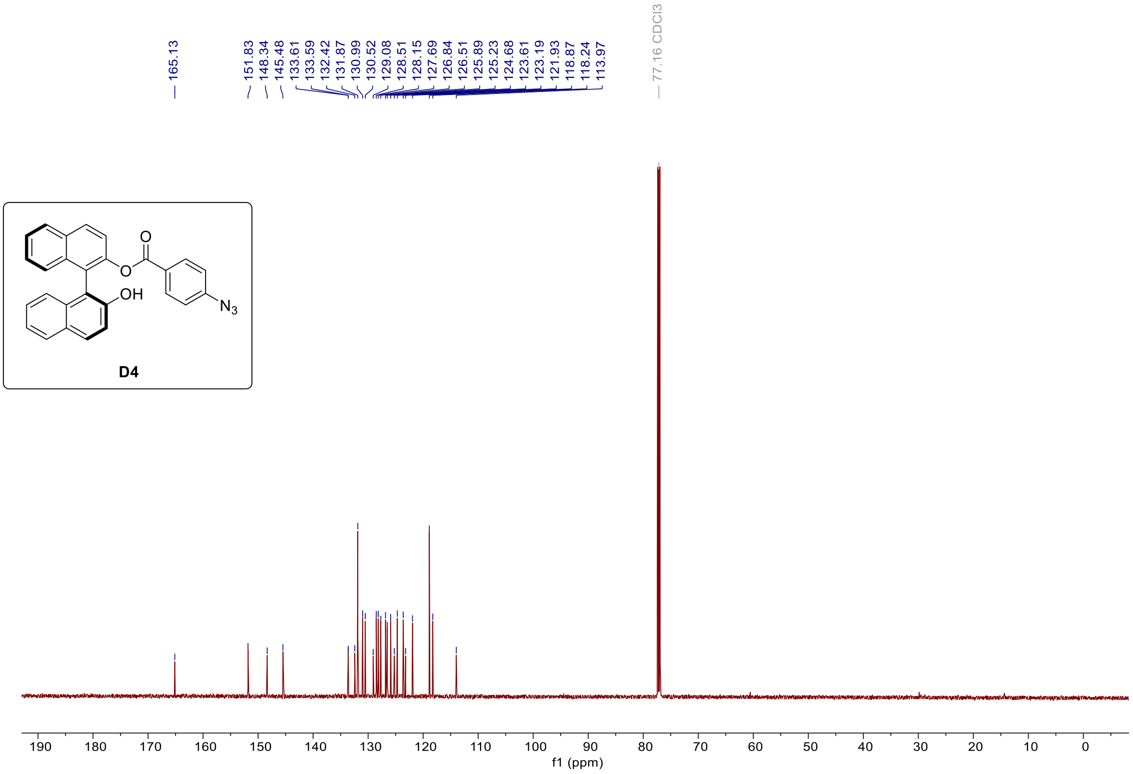

^{1}H NMR (600 MHz, $CDCl_3$) δ 9.77 (t, *J* = 1.7 Hz, 1H), 2.47 (dt, *J* = 7.3, 1.7 Hz, 2H), 2.22 (dt, *J* = 7.0, 2.7 Hz, 2H), 1.96 (t, *J* = 2.7 Hz, 1H), 1.80–1.72 (m, 2H), 1.61–1.54 (m, 2H).

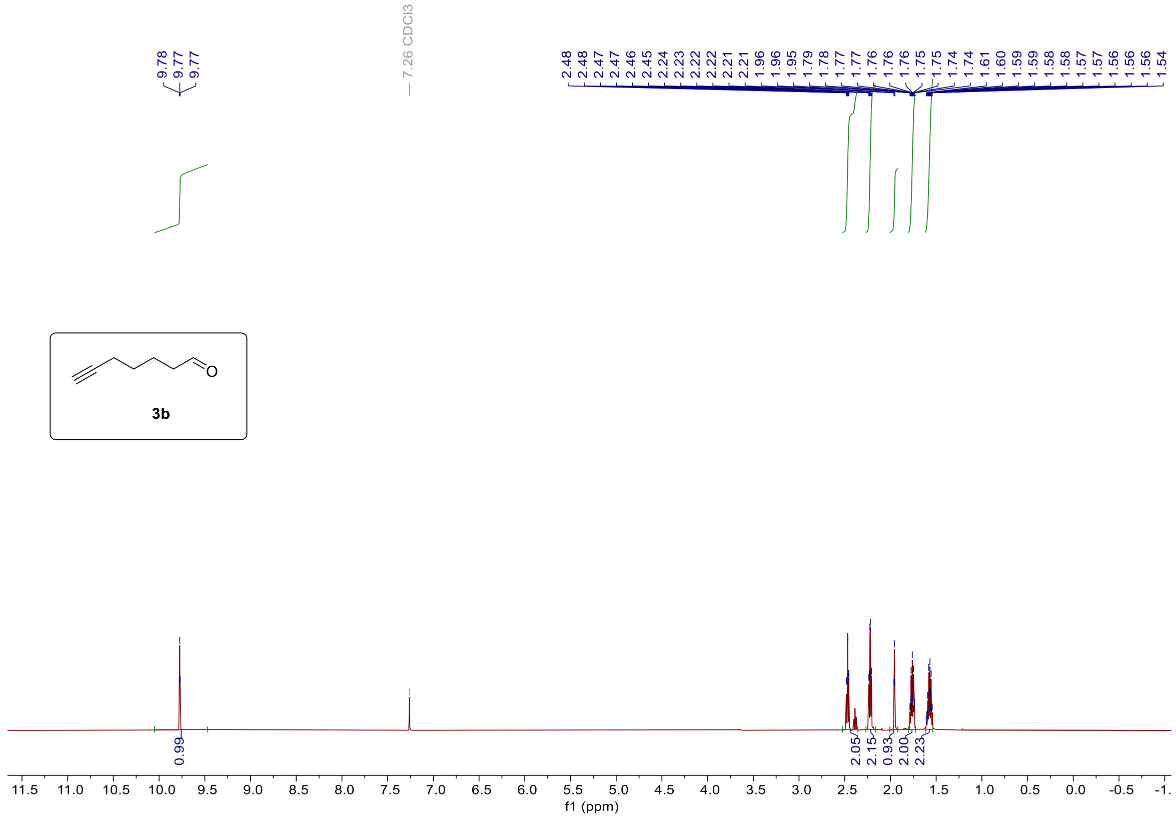

^{13}C NMR (151 MHz, $CDCl_3$) δ 202.37, 83.91, 68.89, 43.44, 27.90, 21.22, 18.33.

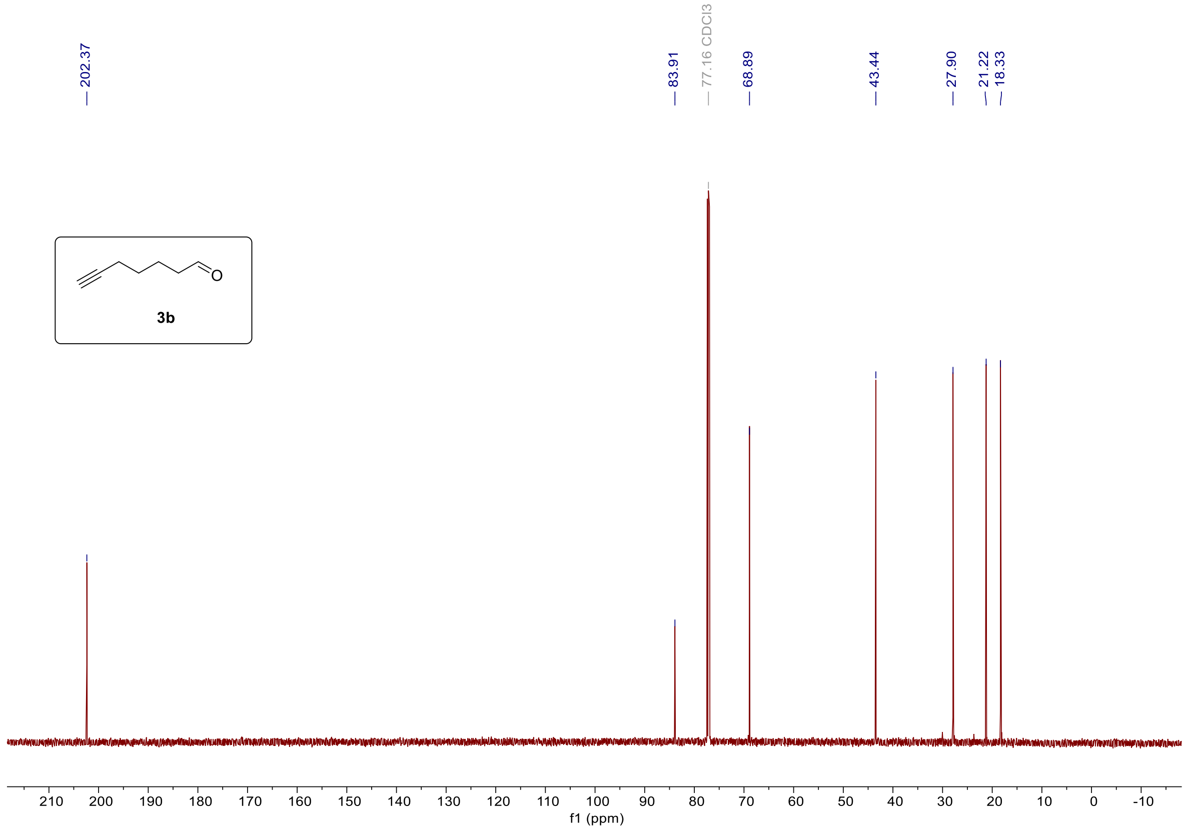

^{1}H NMR (600 MHz, $CDCl_3$) δ 9.73 (t, *J* = 1.8, 1H), 2.42 (dt, *J* = 7.2, 2.1 Hz, 2H), 2.17 (dt, *J* = 7.0, 2.6 Hz, 2H), 1.93 (t, *J* = 1.8, 1H), 1.67–1.58 (m, 2H), 1.56–1.48 (m, 2H), 1.46–1.37 (m, 2H), 1.37–1.30 (m, 2H).

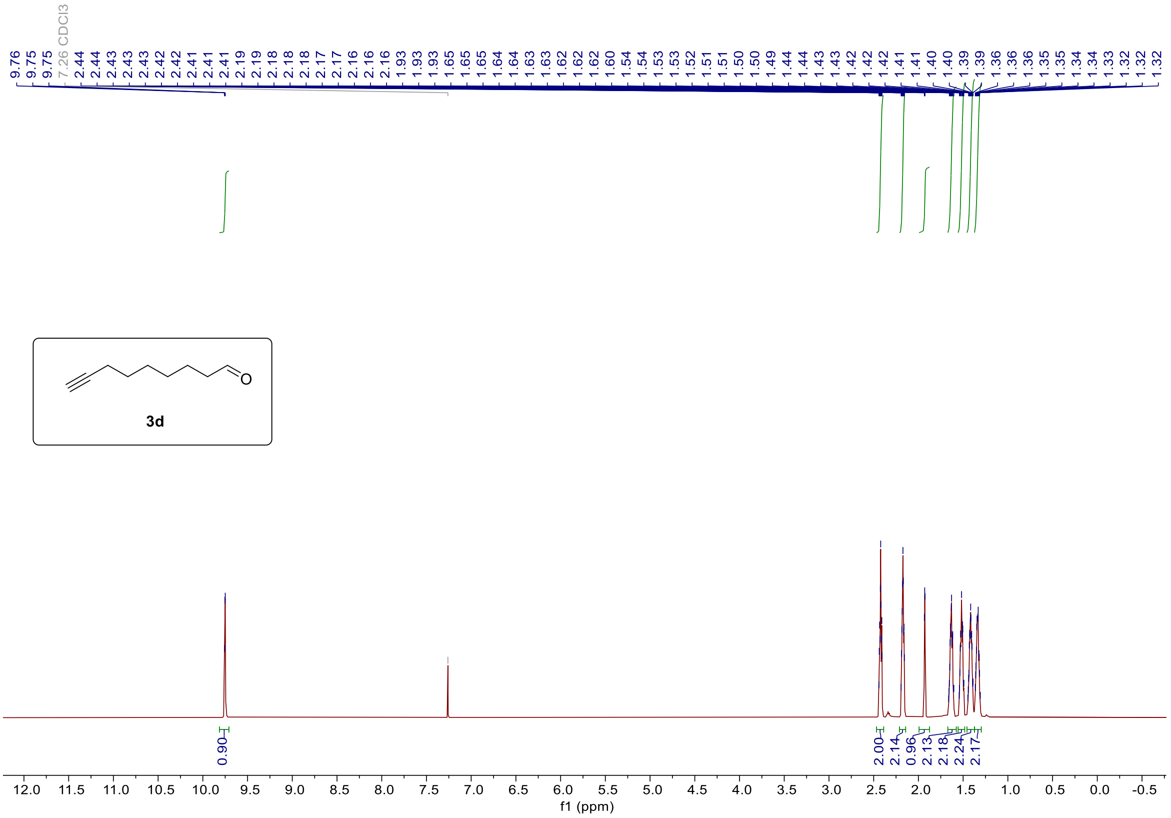

^{13}C NMR (151 MHz, $CDCl_3$) δ 202.86, 84.57, 68.40, 43.92, 28.72, 28.51, 28.29, 22.02, 18.42.

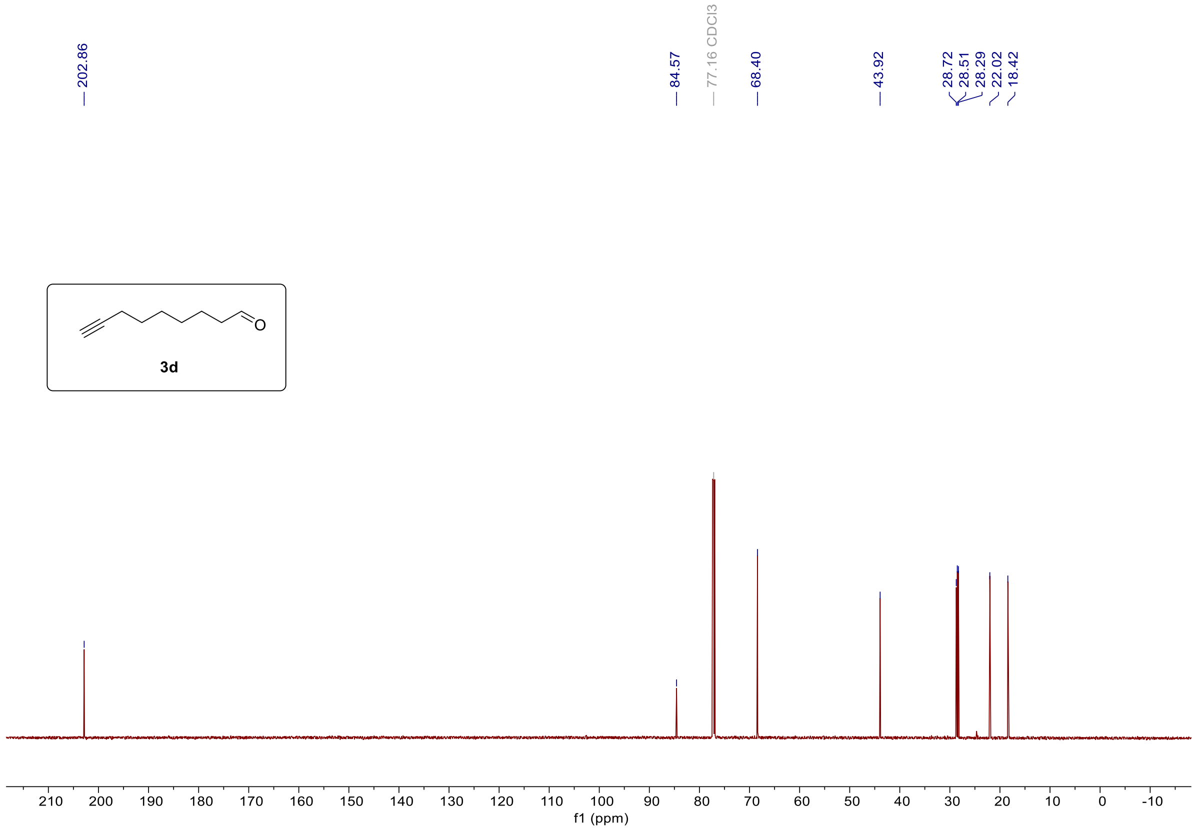

^{1}H NMR (600 MHz, $CDCl_3$) δ 7.94 (d, *J* = 8.7 Hz, 2H), 6.92 (d, *J* = 8.8 Hz, 2H), 3.86 (s, 3H), 3.06–2.81 (m, 2H), 1.75–1.66 (m, 2H), 1.36–1.43 (m, 2H), 0.94 (t, *J* = 7.4 Hz, 3H).

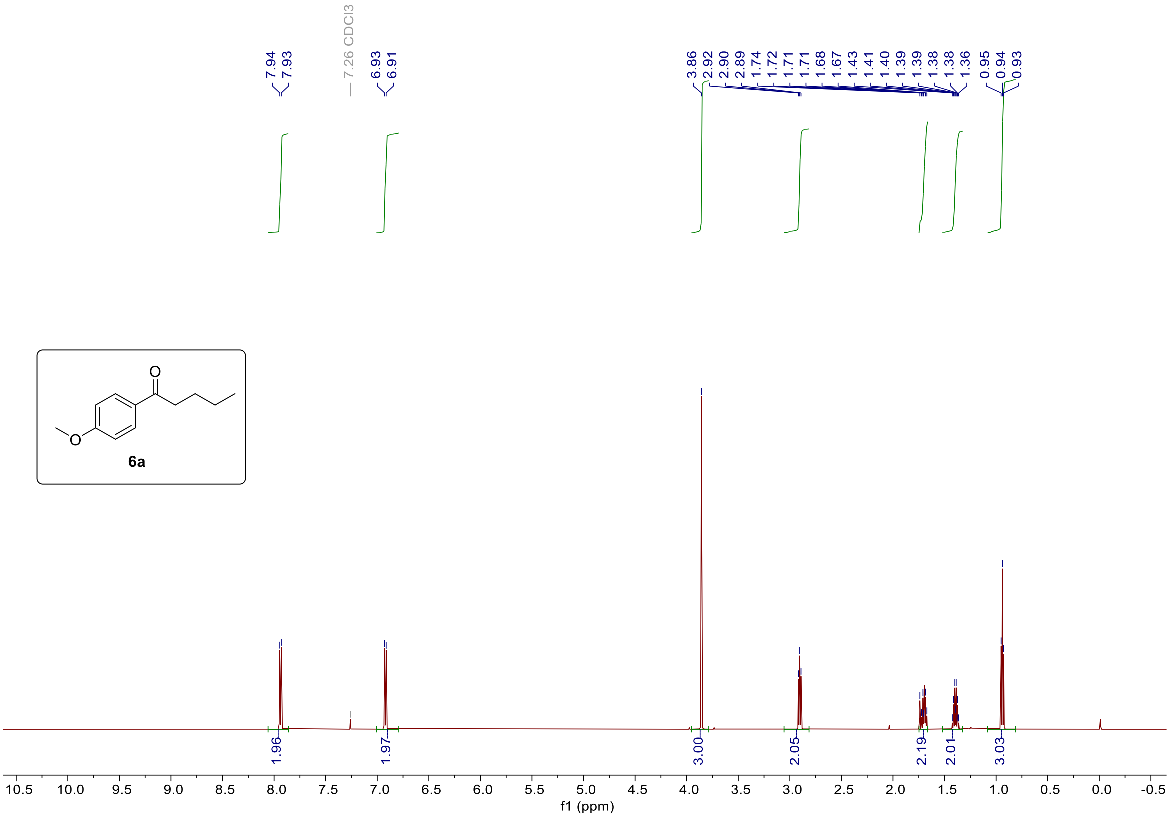

^{13}C NMR (151 MHz, $CDCl_3$) δ 199.40, 163.39, 130.43, 130.25, 113.75, 55.56, 38.13, 26.84, 22.65, 14.09.

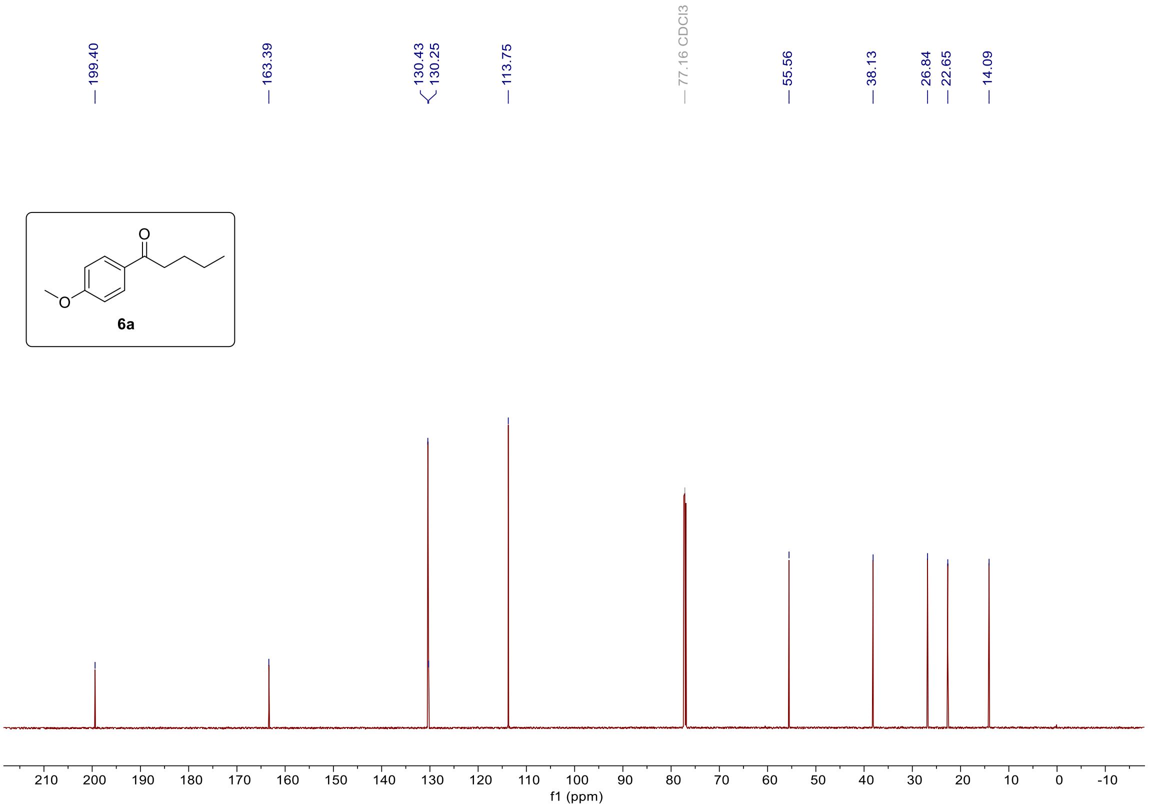

^{1}H NMR (600 MHz, $CDCl_3$) δ 7.96 (d, *J* = 8.8 Hz, 2H), 7.01 (d, *J* = 8.8 Hz, 2H), 4.75 (d, *J* = 2.4 Hz, 2H), 2.96–2.87 (m, 2H), 2.55 (t, *J* = 2.4 Hz, 1H), 1.74–1.67 (m, 2H), 1.46-1.34(m, 2H), 0.94 (t, *J* = 7.3 Hz, 3H).

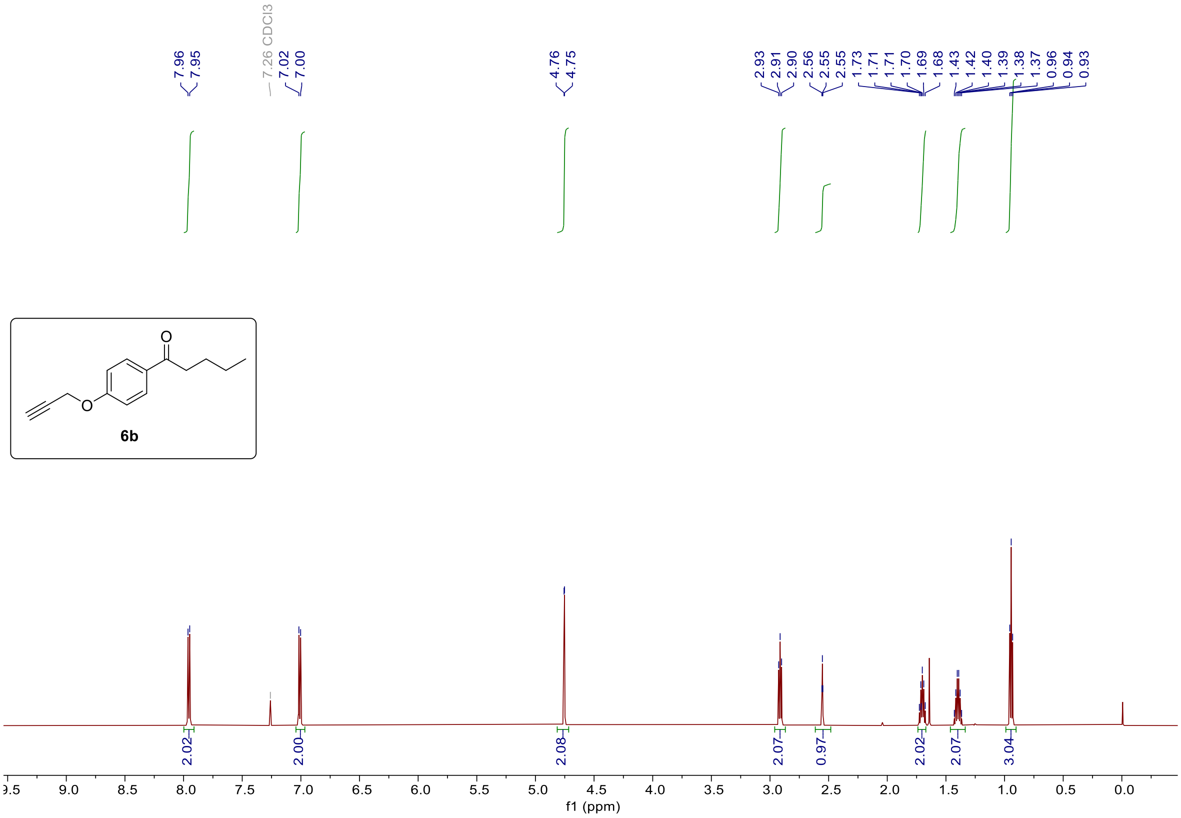

^{13}C NMR (151 MHz, $CDCl_3$) δ 199.37, 161.20, 131.00, 130.38, 114.65, 77.91, 76.26, 55.93, 38.20, 26.79, 22.66, 14.10.

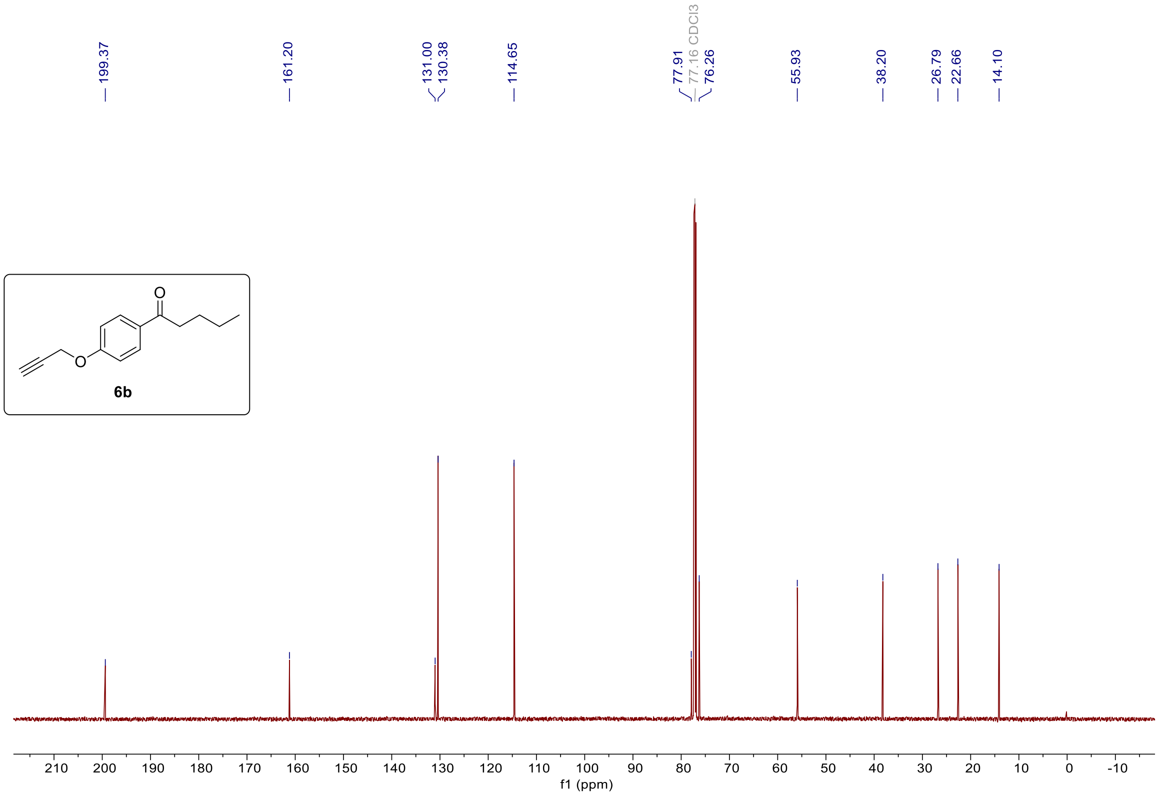

^{1}H NMR (600 MHz, $CDCl_3$) δ 7.34–7.17 (m, 2H), 6.98–6.73 (m, 2H), 4.58 (t, J = 6.8 Hz, 1H), 3.79 (s, 3H), 2.04 (s, 1H), 1.82–1.60 (m, 2H), 1.44–1.14 (m, 4H), 0.87 (t, J = 7.1 Hz, 3H).

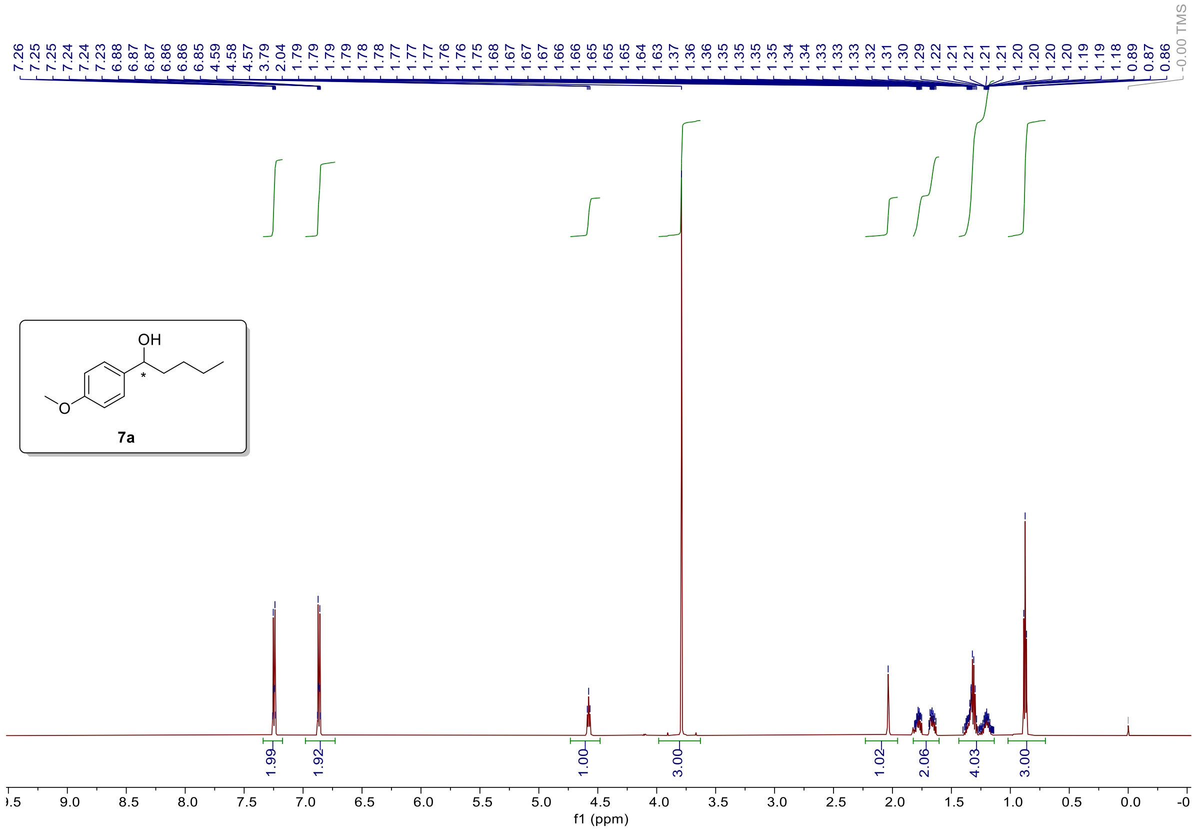

^{13}C NMR (151 MHz, $CDCl_3$) δ 159.01, 137.20, 127.25, 113.83, 74.34, 55.34, 38.78, 28.15, 22.71, 14.14.

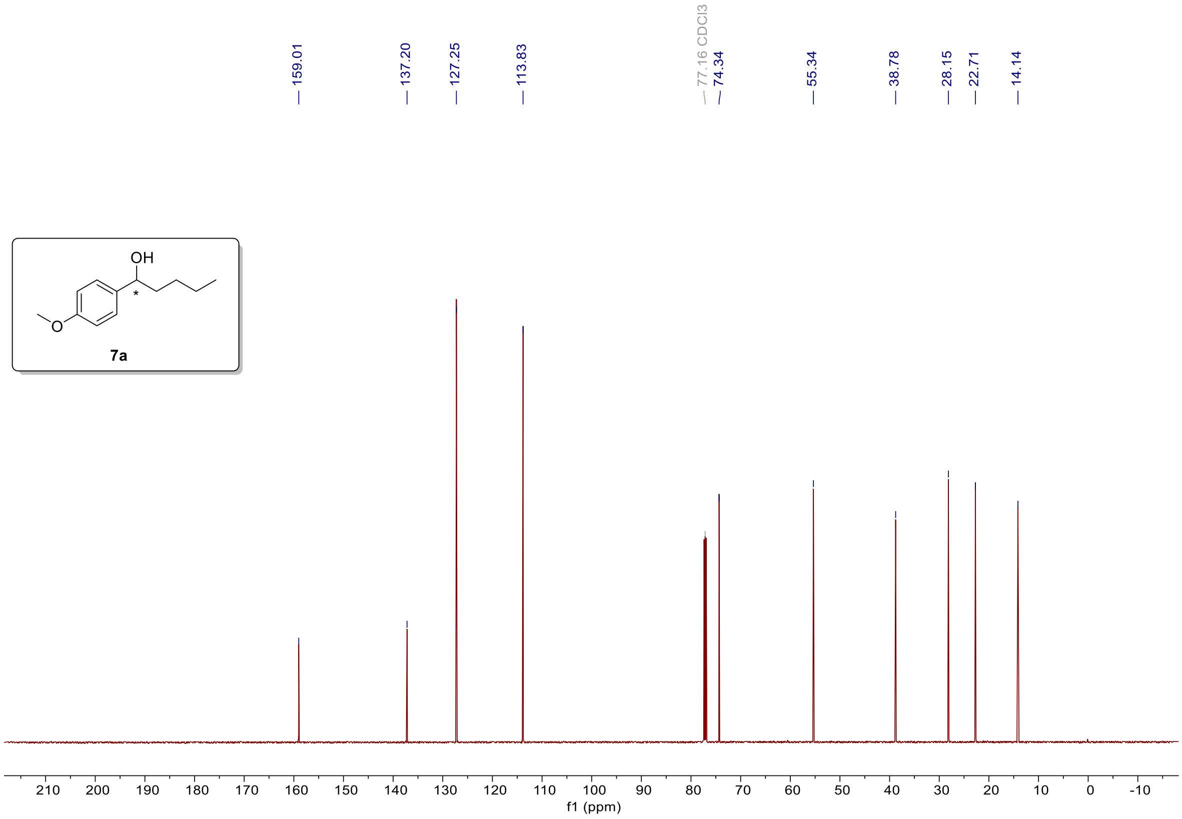

^{1}H NMR (600 MHz, $CDCl_3$) δ 7.32–7.26 (m, 2H), 6.99–6.92 (m, 2H), 4.69 (d, J = 2.4 Hz, 2H), 4.62 (t, J = 13.5 Hz, 1H), 2.52 (t, J = 2.4 Hz, 1H), 1.87–1.73 (m, 2H), 1.72–1.64 (m, 1H), 1.41–1.19 (m, 4H), 0.88 (t, J = 7.2 Hz, 3H).

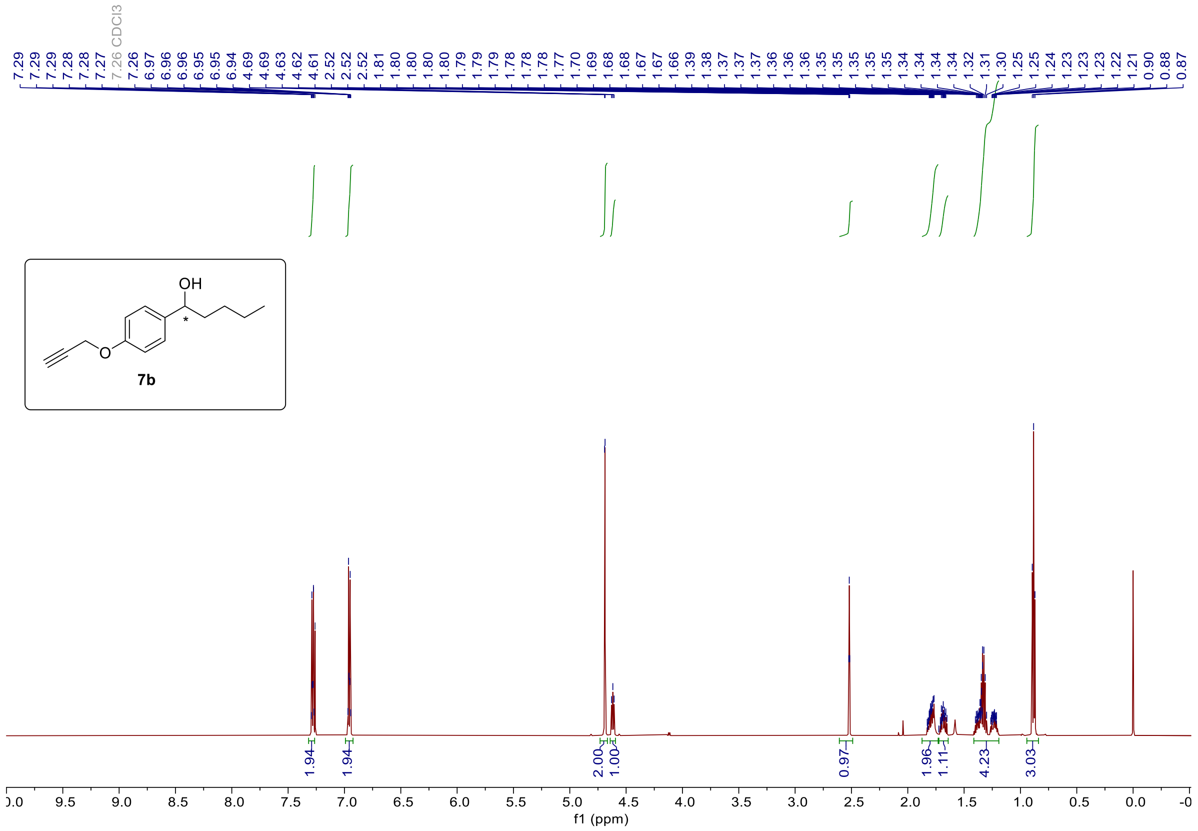

^{13}C NMR (151 MHz, $CDCl_3$) δ 157.09, 138.20, 127.30, 114.93, 78.73, 75.64, 74.39, 55.98, 38.85, 28.17, 22.74, 14.16.

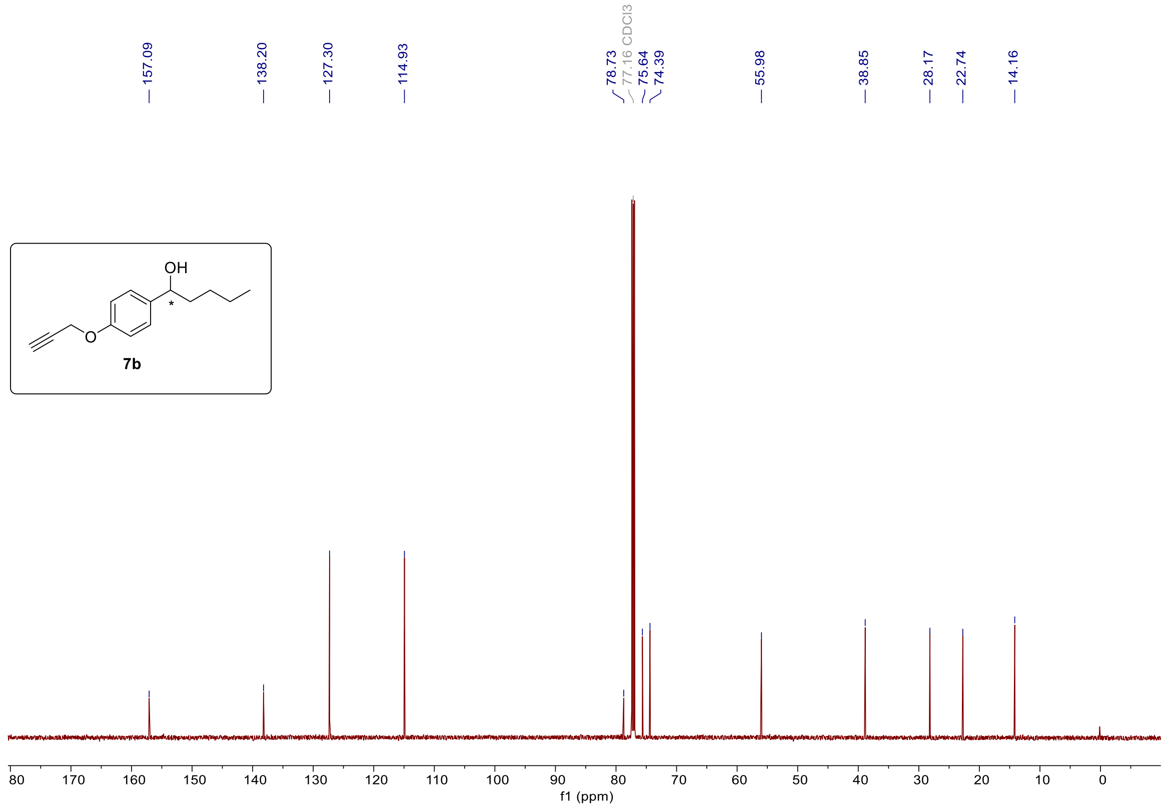

^{1}H NMR (600 MHz, CD_3OD) δ 4.76 (s, 1H), 4.27 (q, *J* = 7.0 Hz, 1H), 3.06 (s, 3H), 1.56 (d, *J* = 7.2 Hz, 3H), 1.16 (s, 9H).

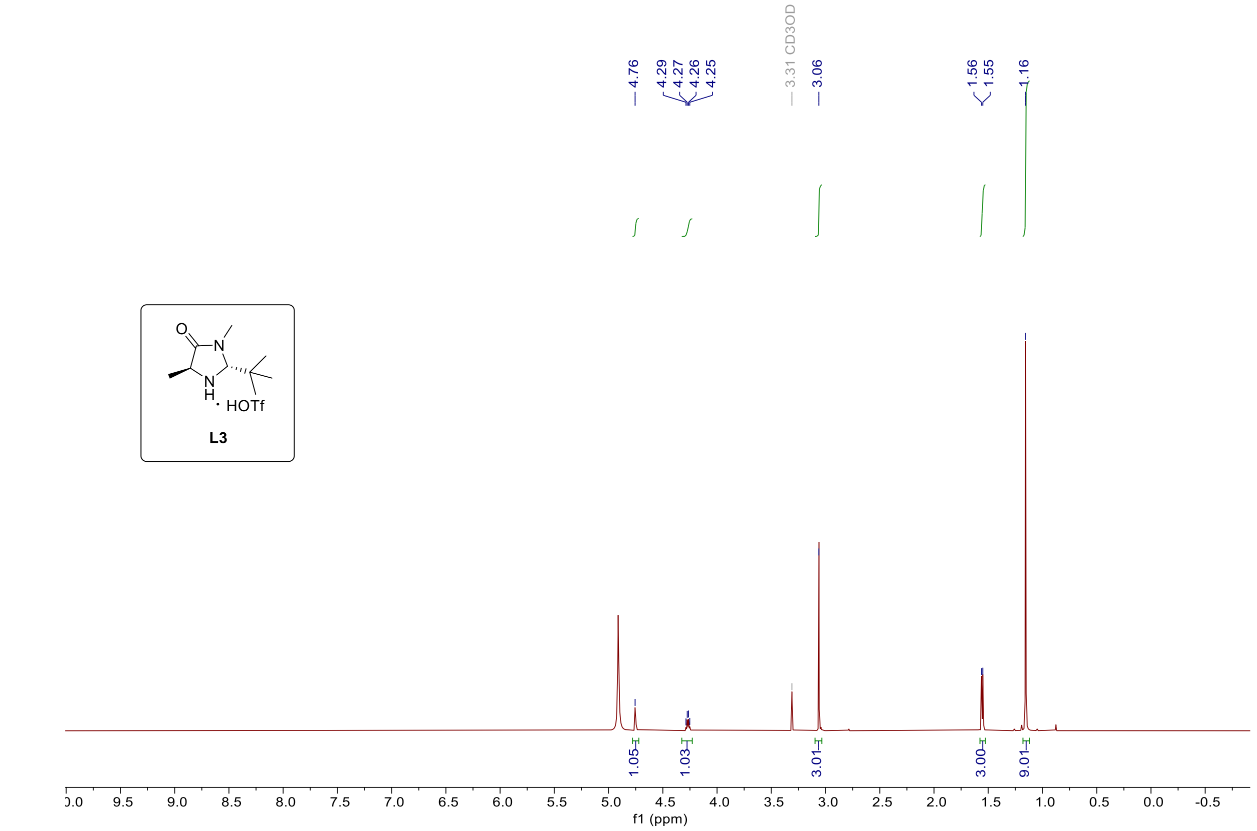

^{13}C NMR (151 MHz, CD_3OD) δ 170.98, 81.76, 54.74, 37.53, 32.36, 25.26, 14.66.

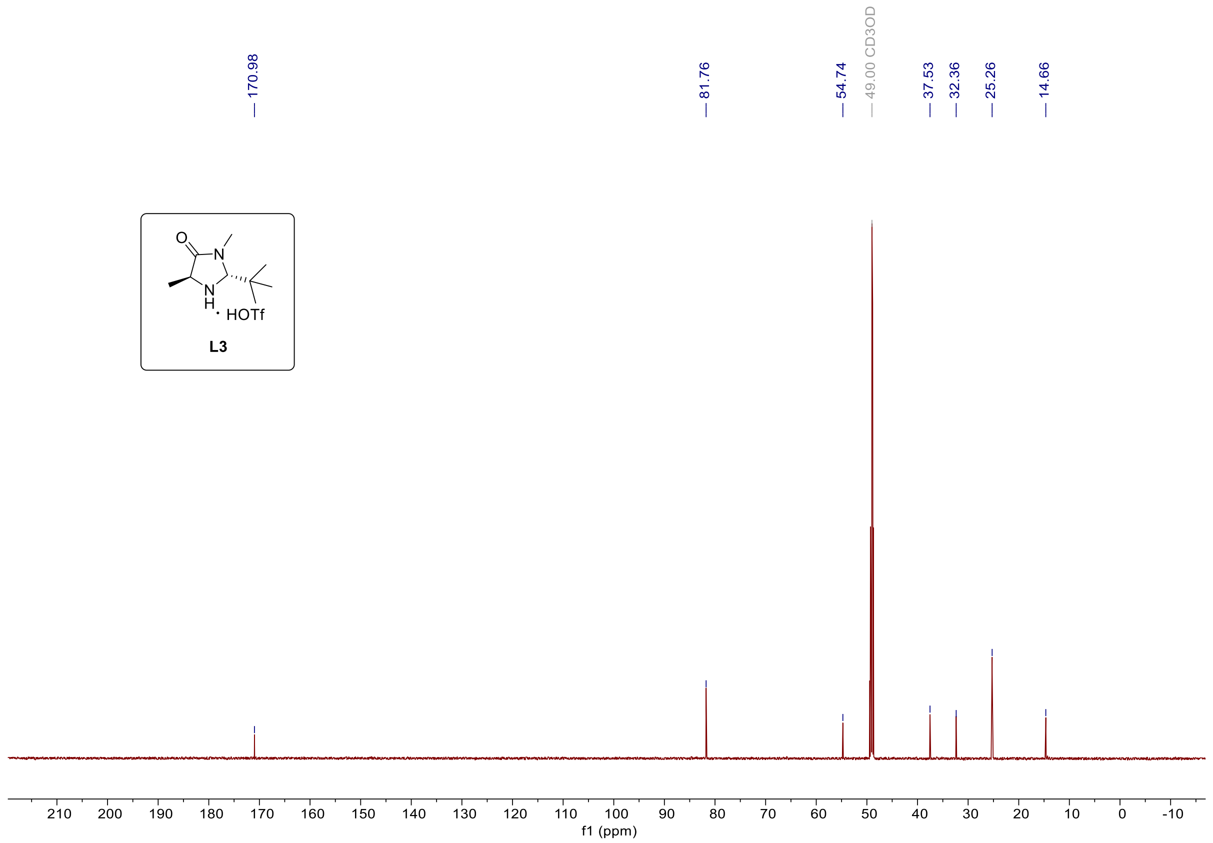

^1H NMR (600 MHz, $CDCl_3$) δ 7.74–7.54 (m, 4H), 7.38–7.28 (m, 6H), 7.24–7.15 (m, 5H), 4.18 (dd, J = 10.8, 2.6 Hz, 1H), 2.65 (dd, J = 13.9, 2.5 Hz, 1H), 2.46 (dd, J = 13.9, 10.8 Hz, 1H).

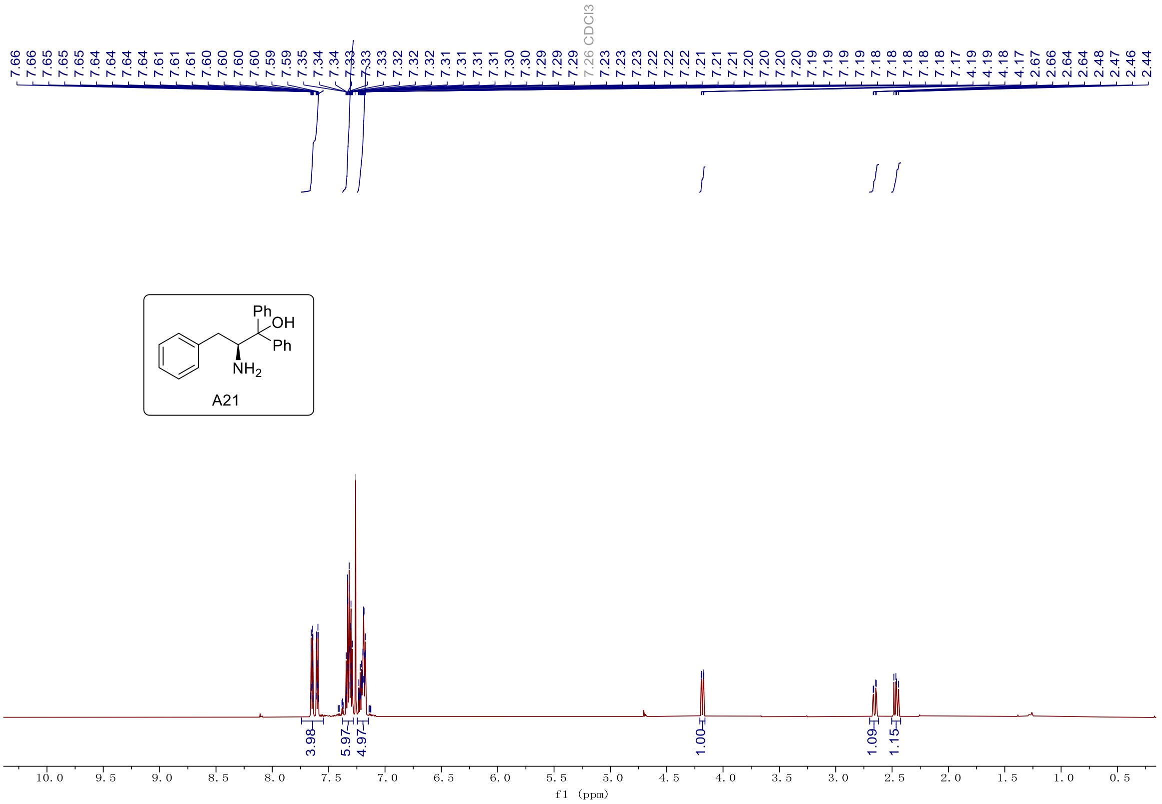

^{13}C NMR (151 MHz, $CDCl_3$) δ 146.89, 144.42, 139.71, 129.26, 128.85, 128.68, 128.42, 126.96, 126.72, 126.65, 125.94, 125.54, 78.66, 58.42, 36.85.

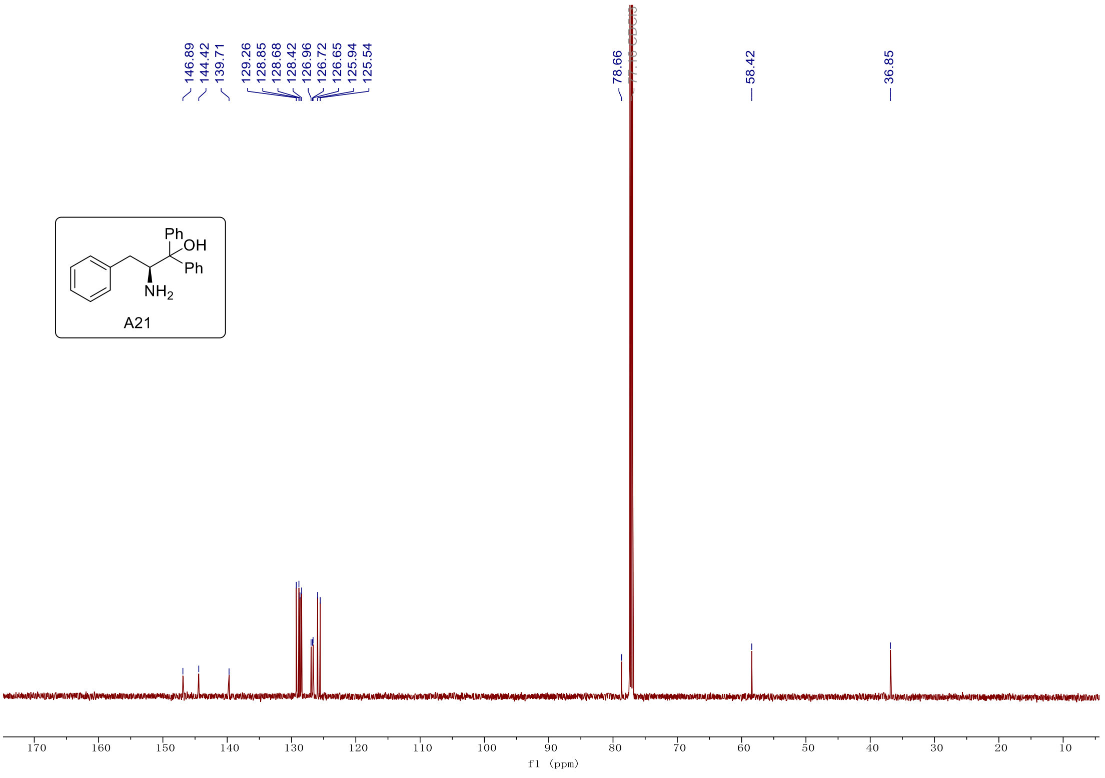

^{1}H NMR (600 MHz, $CDCl_3$) δ 7.55–7.40 (m, 4H), 7.32–7.23 (m, 4H), 7.21–7.11 (m, 2H), 3.85 (dd, J = 5.7, 3.3 Hz, 1H), 3.57 (qd, J = 11.2, 4.4 Hz, 2H).

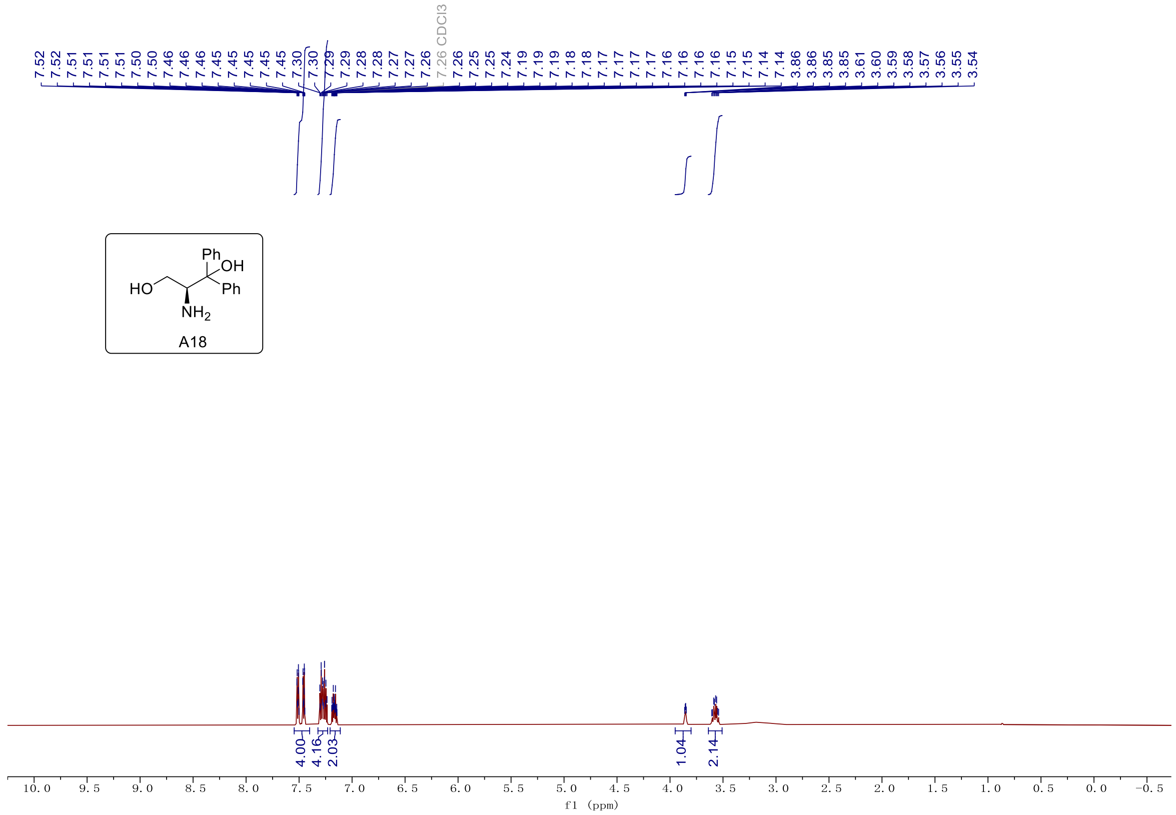

^{13}C NMR (151 MHz, $CDCl_3$) δ 145.56, 144.78, 128.63, 128.37, 126.99, 126.81, 125.53, 125.20, 79.71, 63.12, 56.97.

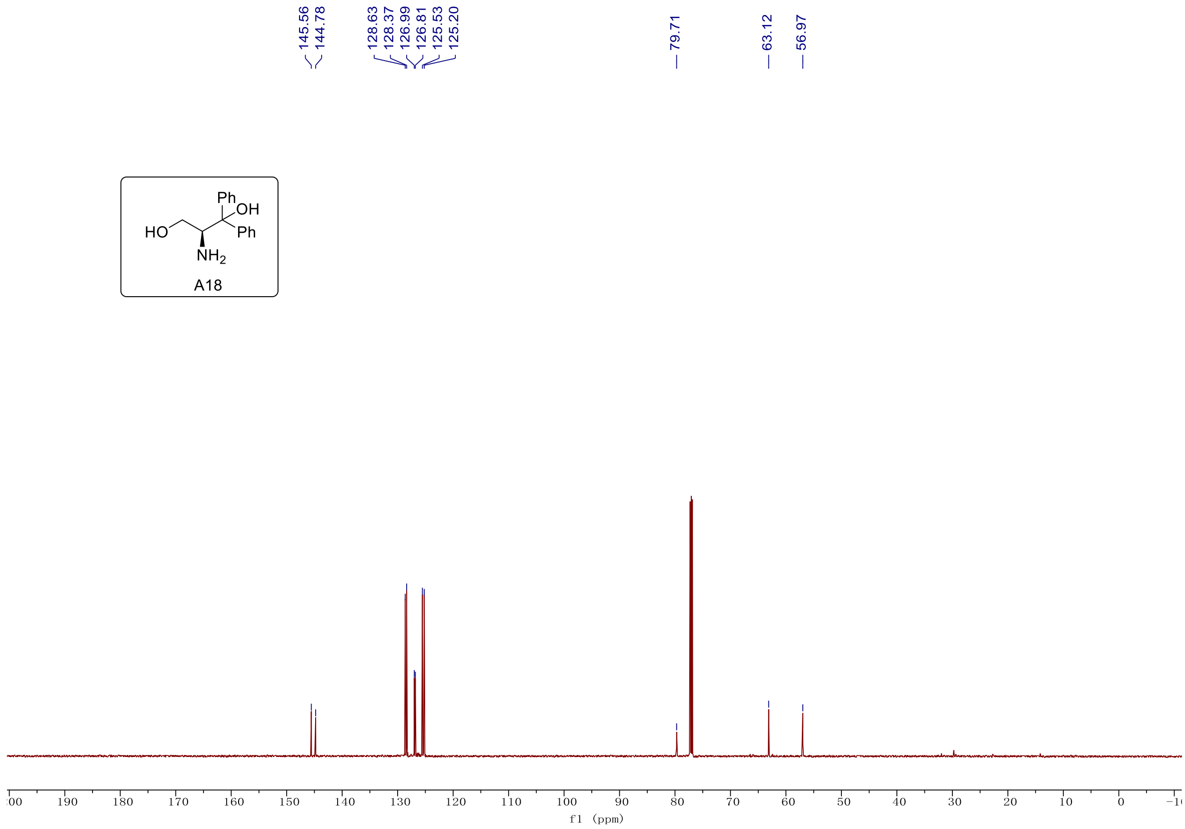

^{1}H NMR (600 MHz, $CDCl_3$) δ 7.59–7.46 (m, 4H), 7.34–7.28 (m, 4H), 7.22–7.15 (m, 2H), 3.96–3.88 (m, 1H), 3.73–

3.67 (m, 1H), 1.22 (d, J = 6.4 Hz, 3H).

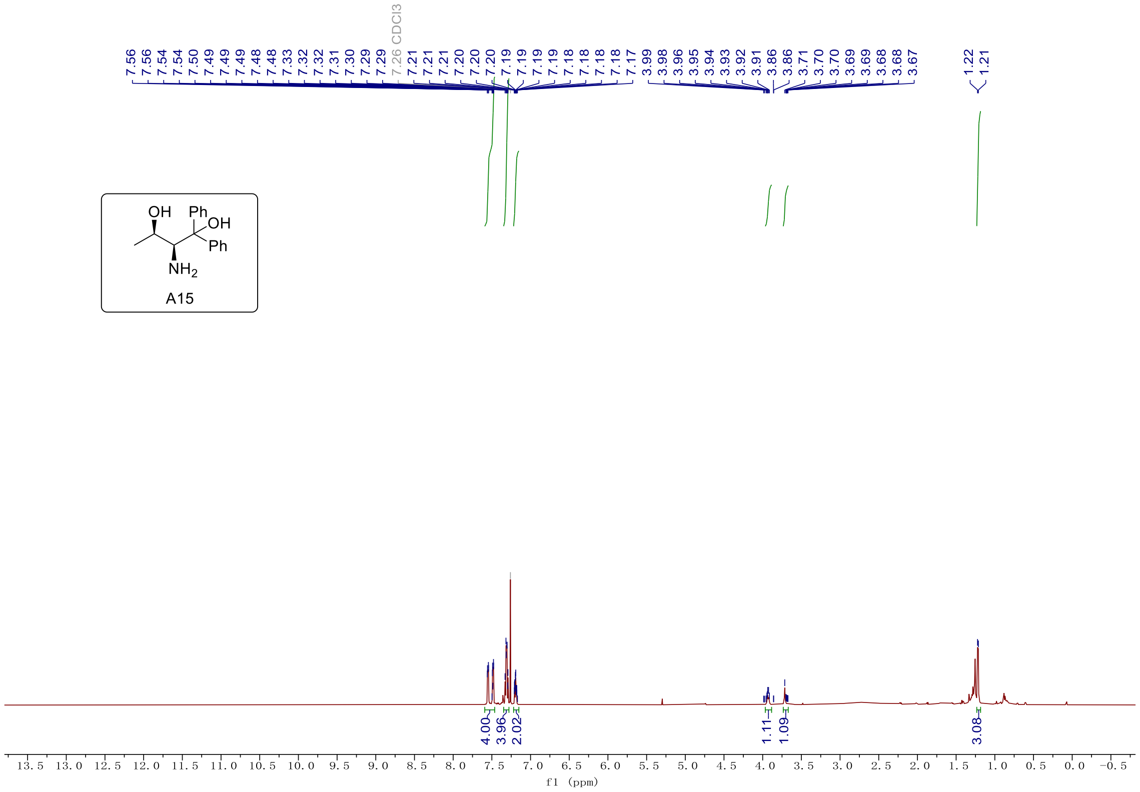

^{13}C NMR (151 MHz, $CDCl_3$) δ 145.38, 144.41, 128.77, 128.65, 127.16, 126.95, 125.64, 125.21, 81.51, 67.06, 59.09, 21.51.

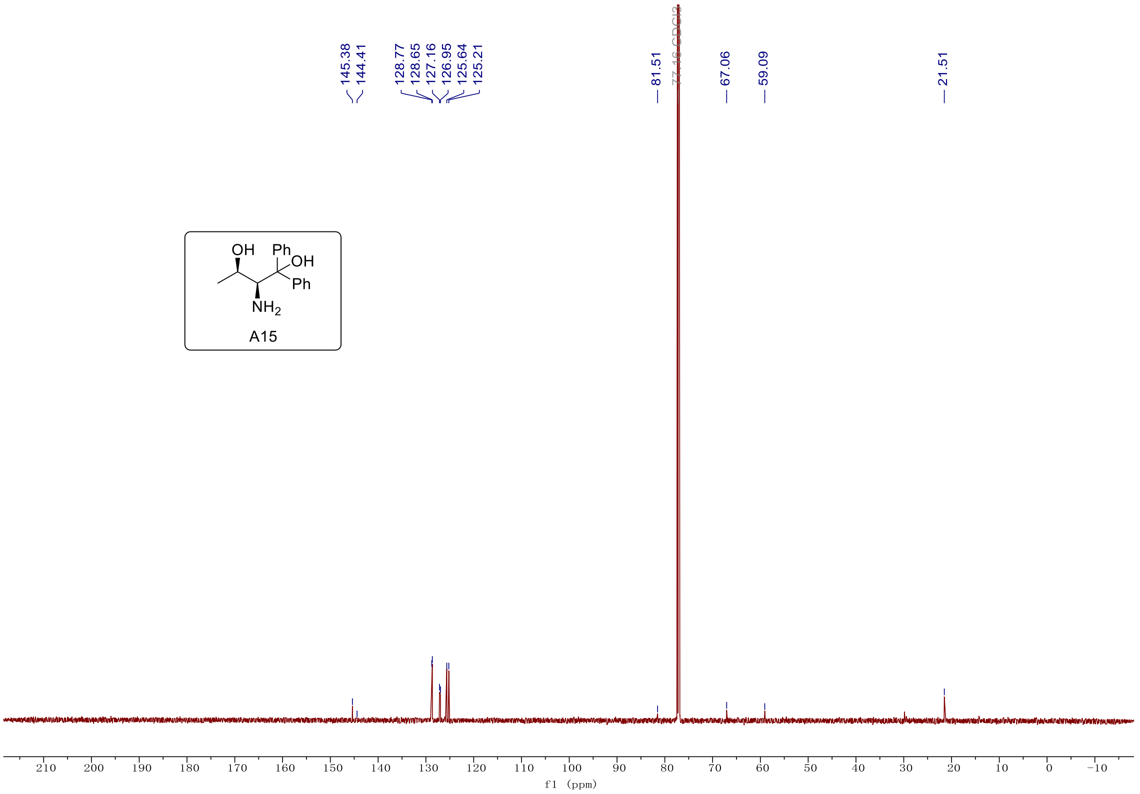

^{1}H NMR (600 MHz, $CDCl_3$) δ 7.42– 7.10 (m, 10H), 5.97 (d, *J* = 8.5 Hz, 1H), 5.38–5.24 (m, 1H), 4.90 (d, *J* = 6.6 Hz, 1H), 4.38 (d, *J* = 4.0 Hz, 2H), 2.28 (d, *J* = 11.8 Hz, 2H), 2.06–1.77 (m, 5H), 1.60–1.21 (m, 12H), 1.20–0.96 (m, 12H), 0.96–0.82 (m, 11H), 0.68 (s, 3H).

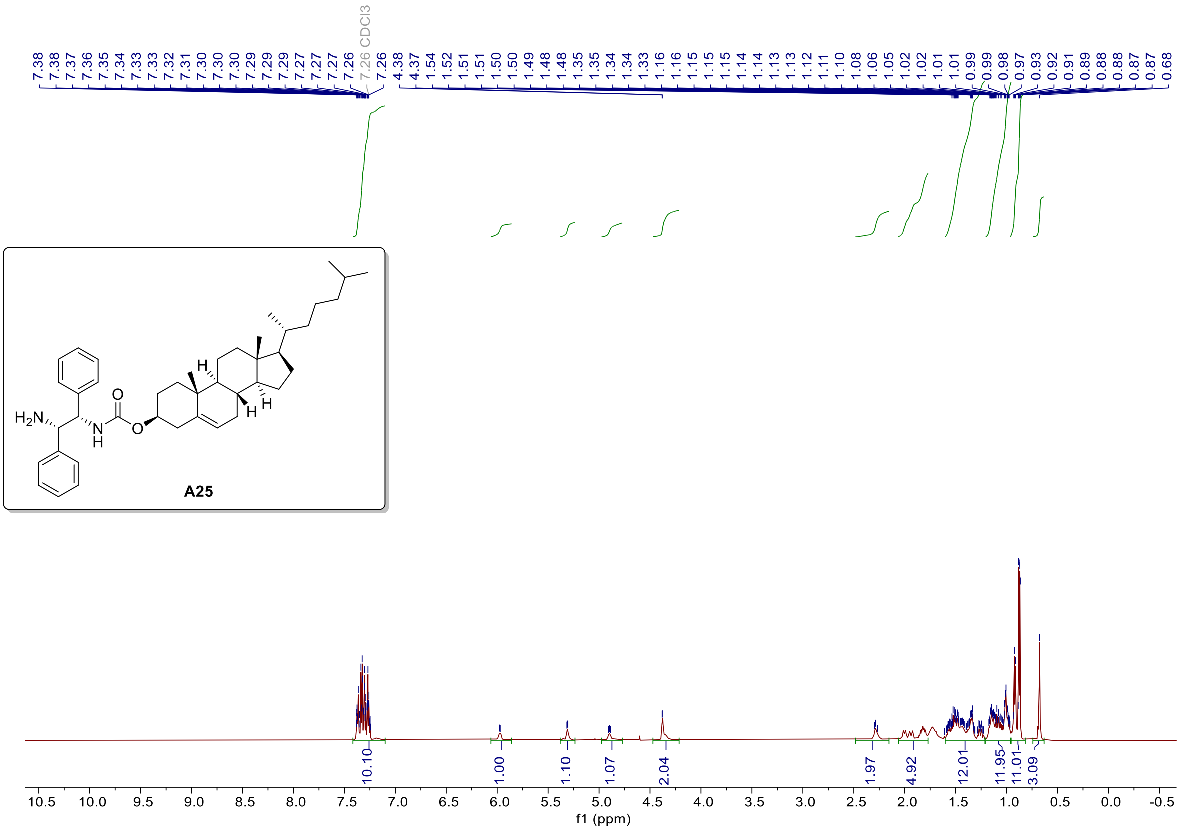

^{13}C NMR (151 MHz, $CDCl_3$) δ 156.01, 142.07, 139.99, 129.03, 128.66, 128.50, 127.65, 127.42, 126.94, 126.65, 126.59, 122.46, 74.46, 60.05, 56.80, 56.24, 50.07, 42.42, 39.85, 39.64, 38.54, 37.04, 36.64, 36.31, 35.93, 32.00, 31.97, 28.36, 28.19, 28.14, 24.40, 23.96, 22.96, 22.70, 21.14, 19.45, 18.84, 12.00, 11.98.

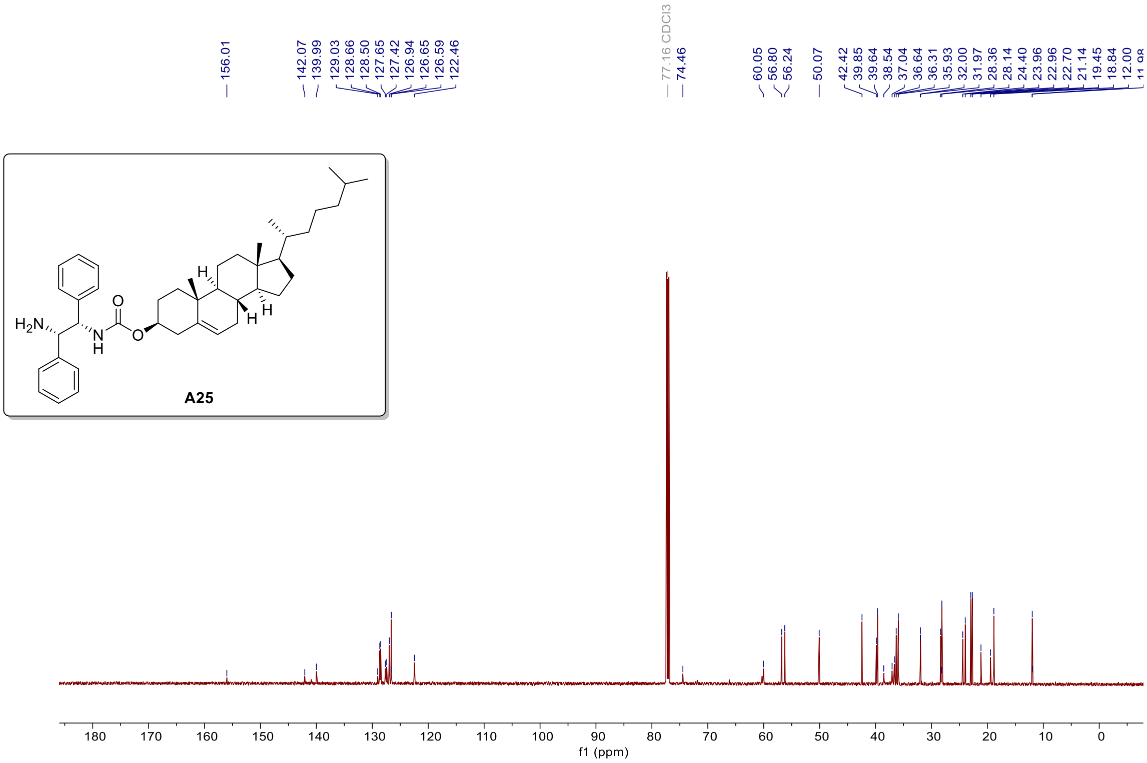

^{1}H NMR (600 MHz, $CDCl_3$) δ 7.46–7.00 (m, 10H), 5.16 (dd, *J* = 8.1, 3.9 Hz, 1H), 4.45 (d, *J* = 3.9 Hz, 1H), 2.14–2.09 (m, 1H), 1.87–1.56 (m, 5H), 1.40–1.12 (m, 5H).

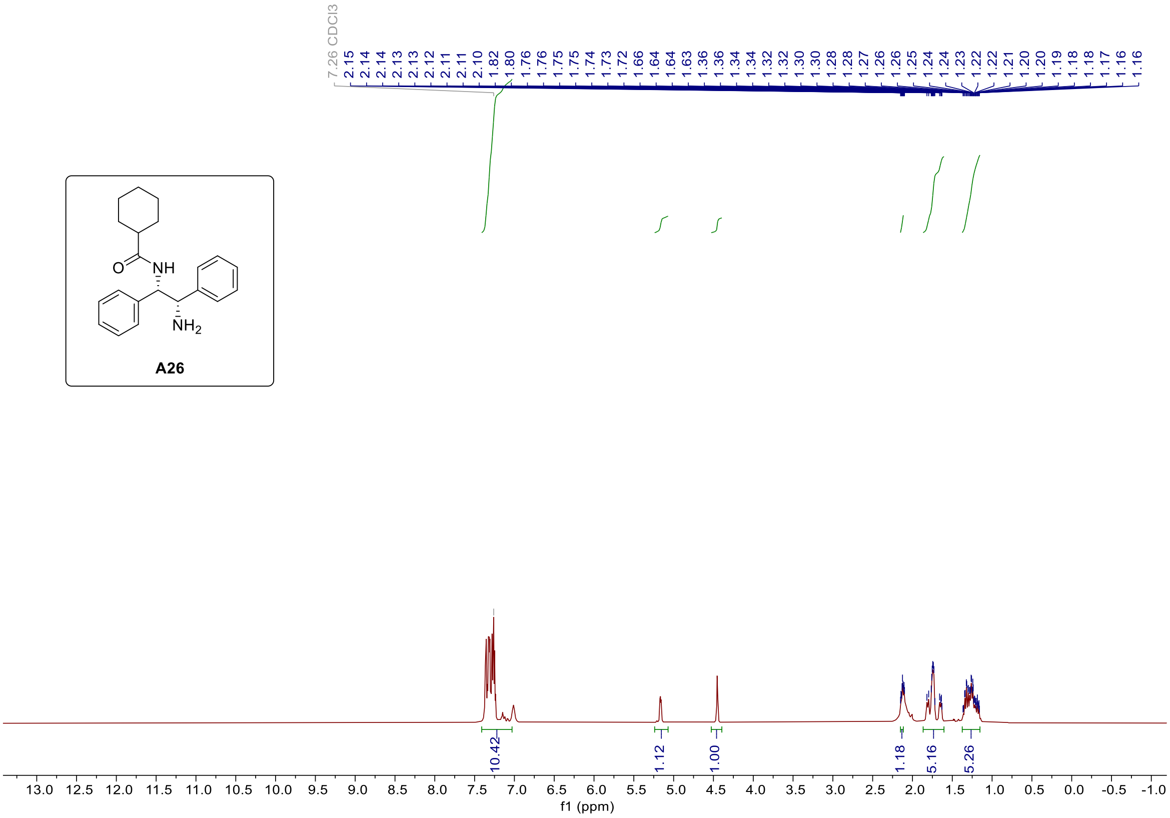

^{13}C NMR (151 MHz, $CDCl_3$) δ 175.87, 140.60, 128.75, 128.55, 127.77, 127.48, 126.76, 126.52, 59.48, 57.98, 45.58, 29.75, 29.74, 25.89, 25.86, 25.84.

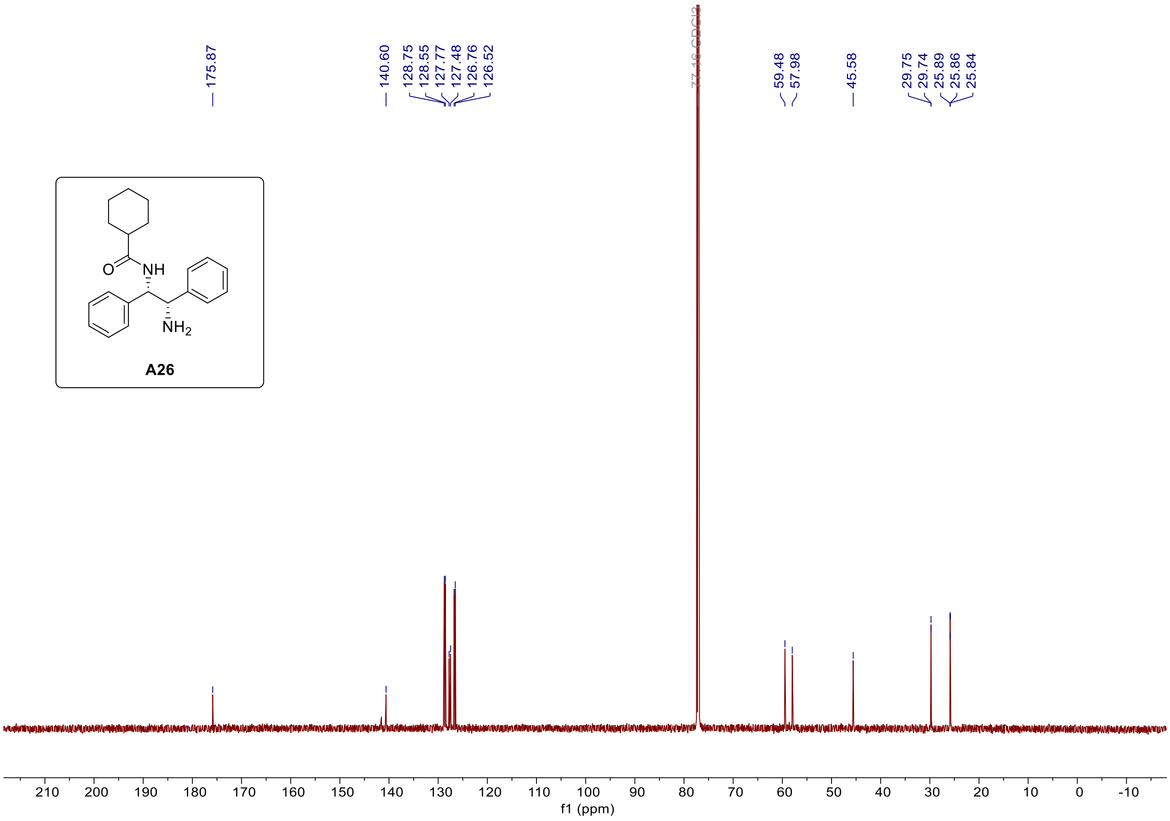

^{1}H NMR (600 MHz, $CDCl_3$) δ 7.73–7.60 (m, 3H), 7.43–7.36 (m, 1H), 7.35–7.30 (m, 4H), 7.27–7.21 (m, 5H), 7.19–7.13 (m, 2H), 5.23 (dd, J = 7.8, 3.6 Hz, 1H), 4.42 (d, J = 3.6 Hz, 1H).

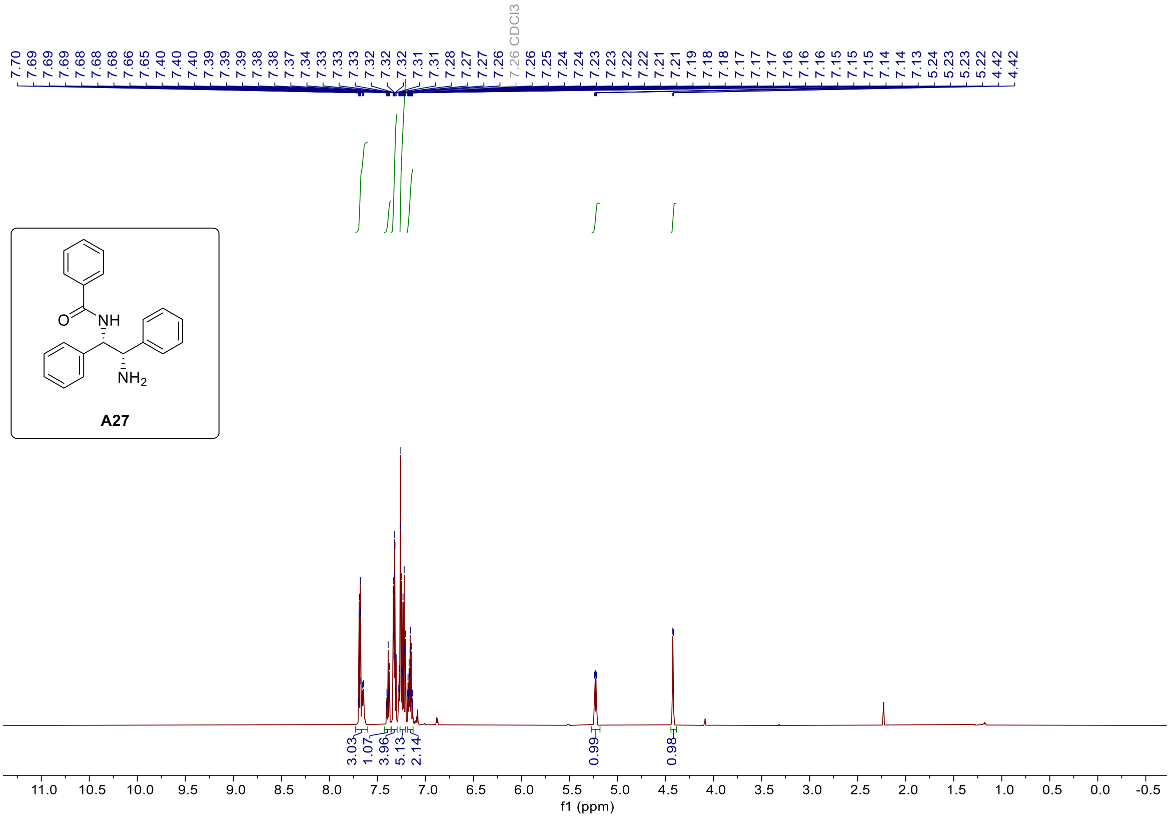

^{13}C NMR (151 MHz, $CDCl_3$) δ 167.00, 141.96, 140.56, 134.60, 131.49, 128.78, 128.63, 128.60, 127.76, 127.52, 127.11, 126.61, 126.49, 59.63, 59.03.

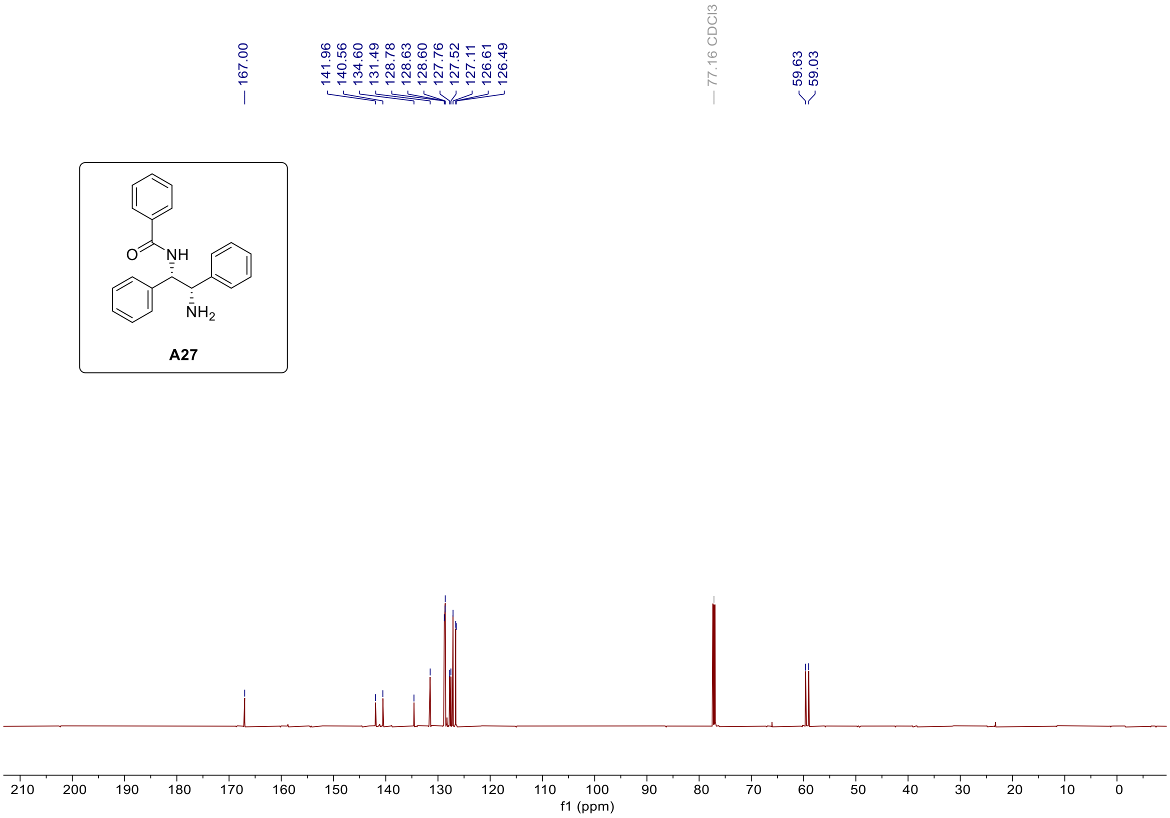

^{1}H NMR (600 MHz, $CDCl_3$) δ 7.44 (d, *J* = 7.7 Hz, 2H), 7.33 (t, *J* = 7.4 Hz, 1H), 7.20–7.04 (m, 12H), 4.48 (d, *J* = 6.1 Hz, 1H), 4.24 (d, *J* = 6.1 Hz, 1H).

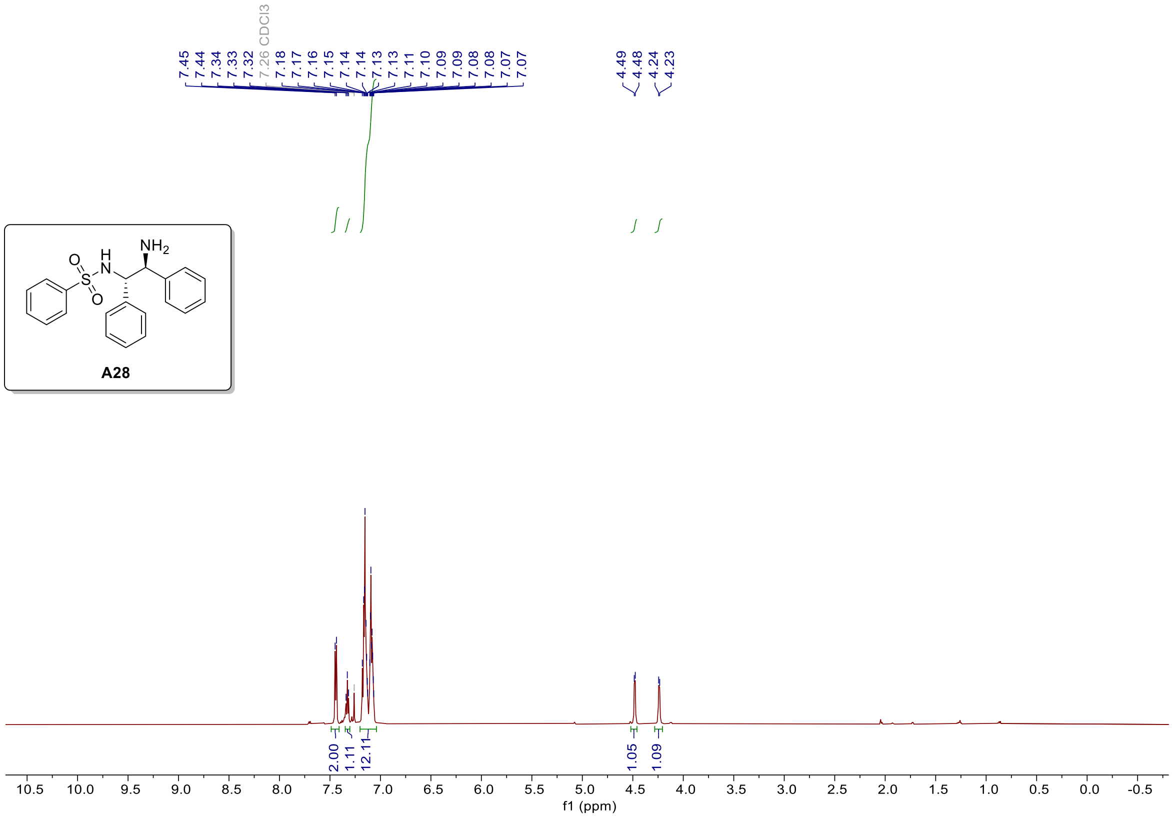

^{13}C NMR (151 MHz, $CDCl_3$) δ 140.73, 140.31, 138.75, 131.96, 128.60, 128.34, 127.86, 127.55, 127.22, 126.95, 126.86, 63.34, 60.48.

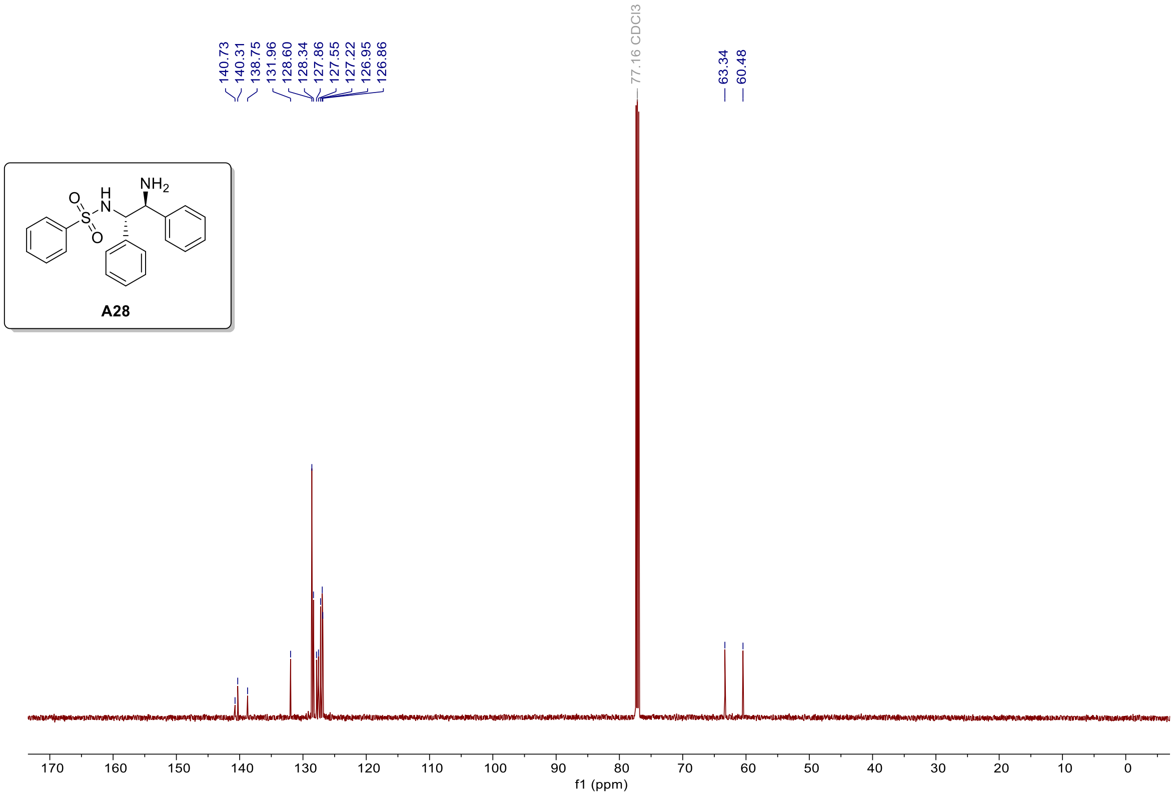

^{1}H NMR (600 MHz, $CDCl_3$) δ 7.43–7.08 (m, 10H), 4.59 (d, *J* = 6.5 Hz, 1H), 4.37 (d, *J* = 6.5 Hz, 1H), 2.27 (s, 3H).

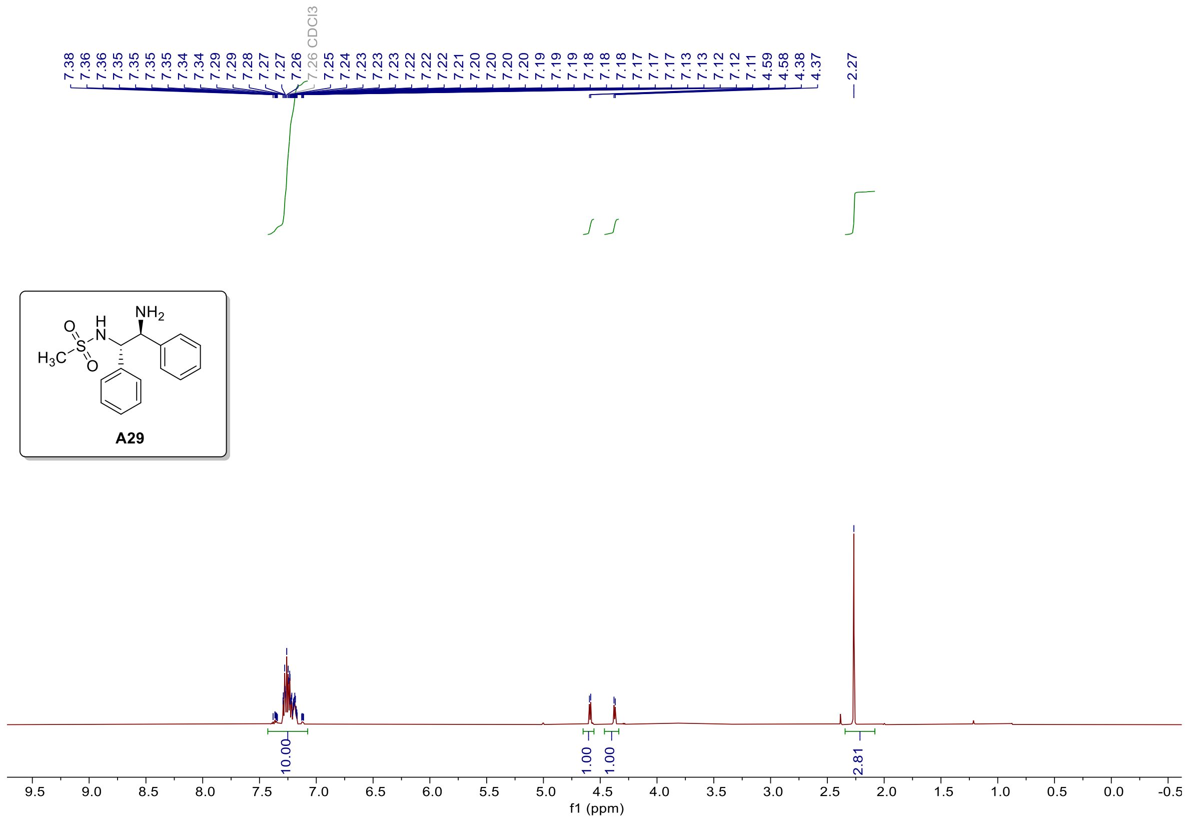

^{13}C NMR (151 MHz, $CDCl_3$) δ 140.38, 139.20, 128.84, 128.80, 128.22, 128.08, 127.31, 127.20, 63.36, 60.09, 41.06.

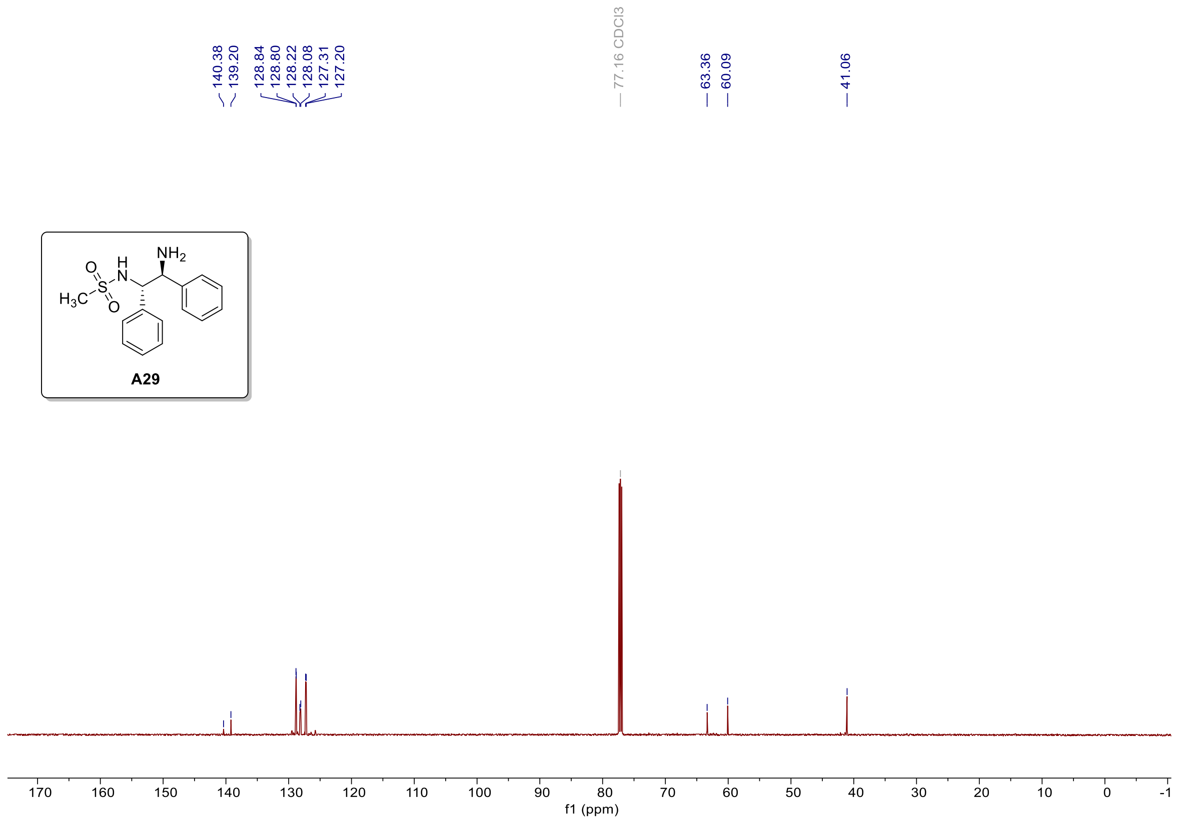

^{1}H NMR (600 MHz, $CDCl_3$) δ 7.34–7.30 (m, 2H), 7.17–7.10 (m, 8H), 6.96 (dd, *J* = 8.3, 2.3 Hz, 2H), 4.42 (t, *J* = 6.3 Hz, 1H), 4.19 (t, *J* = 8.4 Hz, 1H), 2.31 (s, 3H).

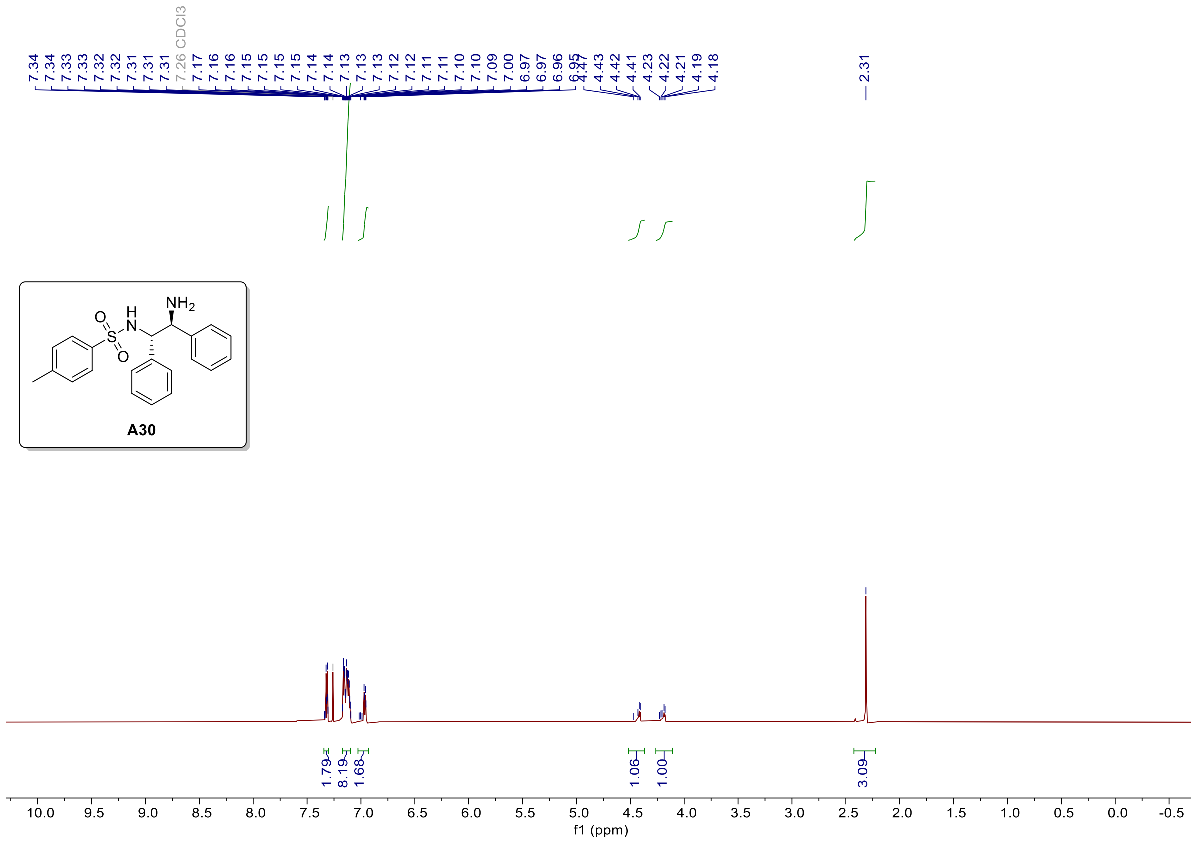

^{13}C NMR (151 MHz, $CDCl_3$) δ 142.60, 141.34, 139.27, 137.27, 129.22, 128.52, 128.33, 127.58, 127.45, 127.16, 126.96, 126.72, 63.33, 60.58, 21.52.

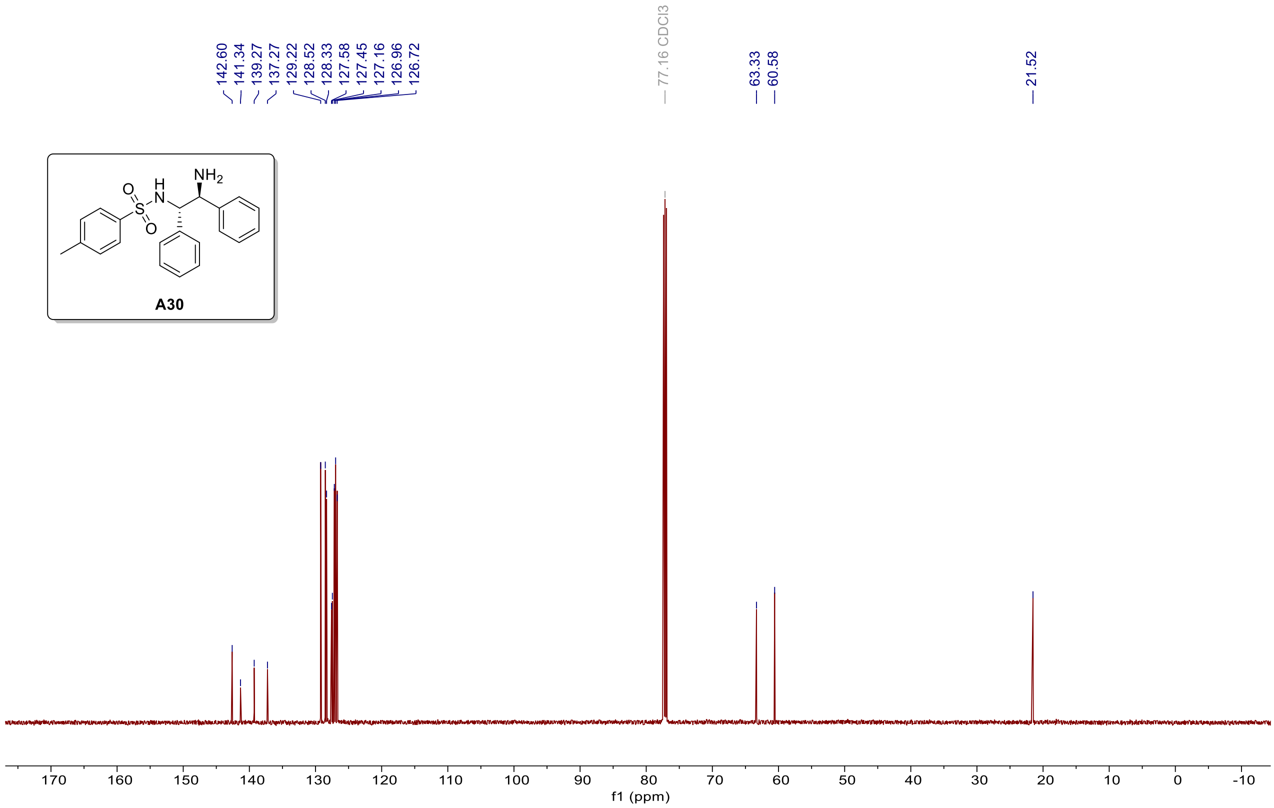

^{1}H NMR (600 MHz, $CDCl_3$) δ 7.39–7.30 (m, 2H), 7.21–7.03 (m, 11H), 4.41 (d, *J* = 5.6 Hz, 1H), 4.16 (d, *J* = 5.6 Hz, 1H), 1.27 (s, 9H).

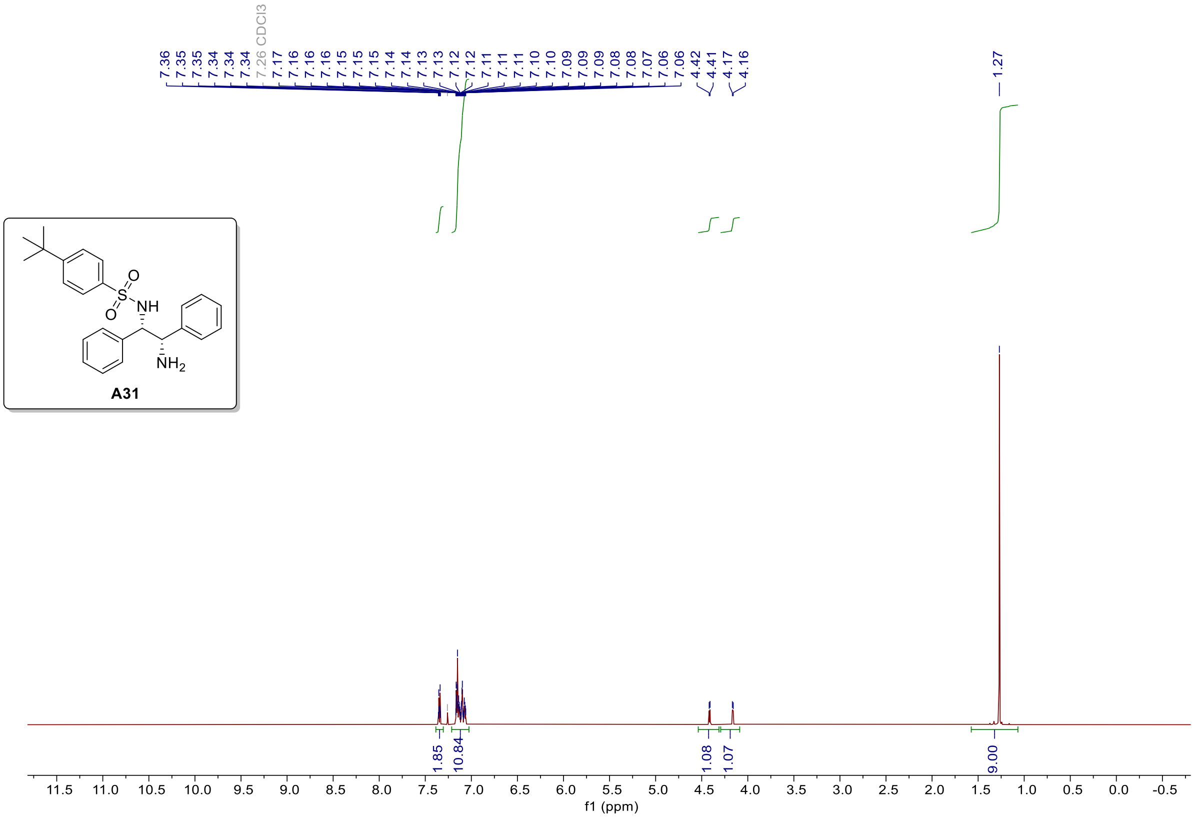

^{13}C NMR (151 MHz, $CDCl_3$) δ 155.49, 141.42, 139.12, 137.12, 128.49, 128.29, 127.68, 127.42, 127.14, 126.82, 126.75, 125.53, 63.37, 60.58, 35.00, 31.17.

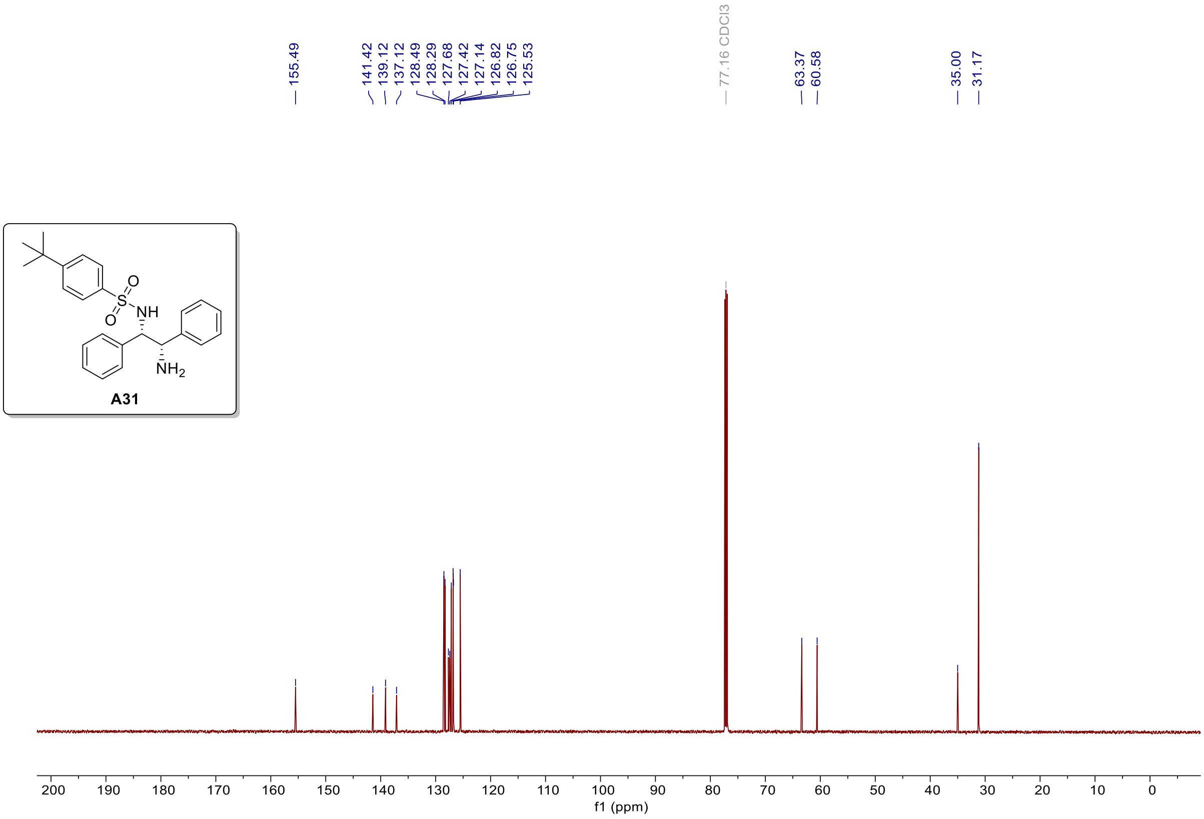

^{1}H NMR (600 MHz, $CDCl_3$) δ 7.20–7.12 (m, 3H), 7.11–7.03 (m, 2H), 7.02–6.88 (m, 5H), 6.84–6.78 (m, 2H), 4.59–4.50 (m, 1H), 4.08–3.88 (m, 3H), 2.87–2.77 (m, 1H), 1.22–1.07 (m, 18H).

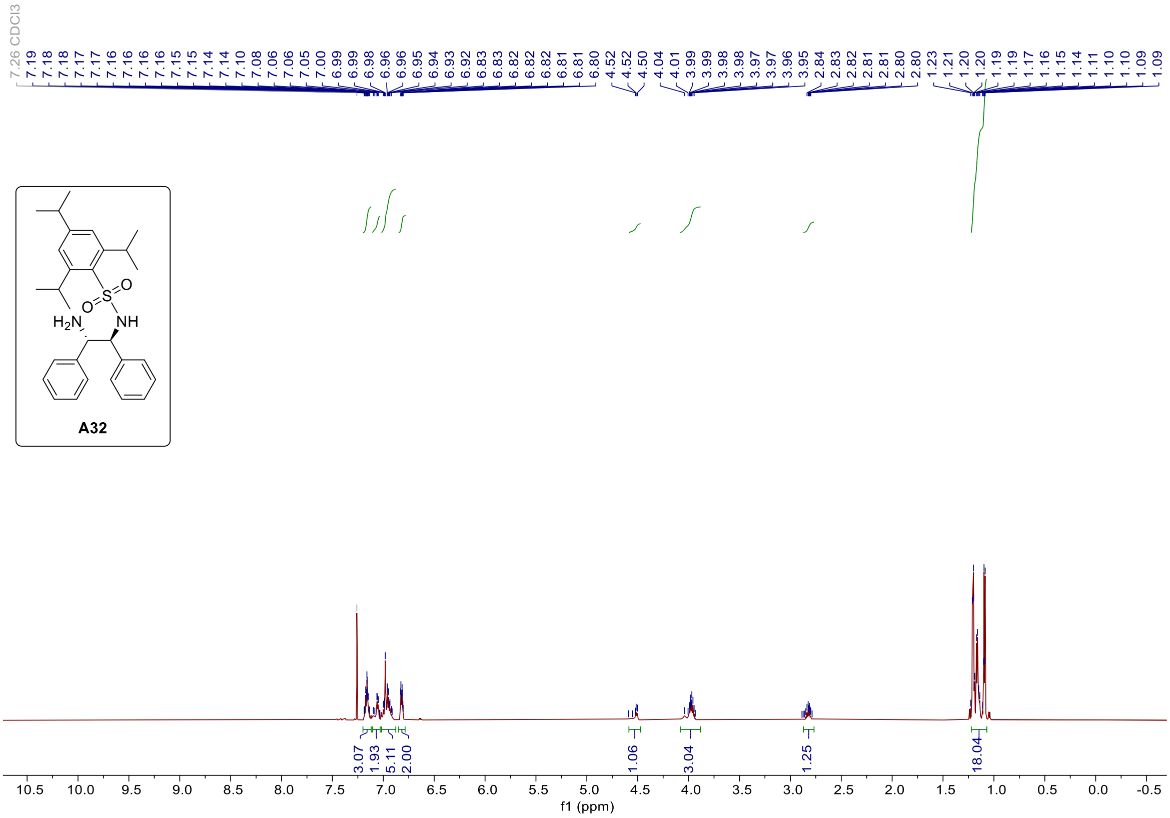

^{13}C NMR (151 MHz, $CDCl_3$) δ 152.45, 149.80, 141.98, 138.75, 134.09, 128.51, 128.51, 127.97, 127.97, 127.66, 127.48, 127.48, 127.48, 127.42, 126.99, 123.41, 63.67, 61.30, 34.27, 29.93, 25.04, 24.90, 23.82, 23.79.

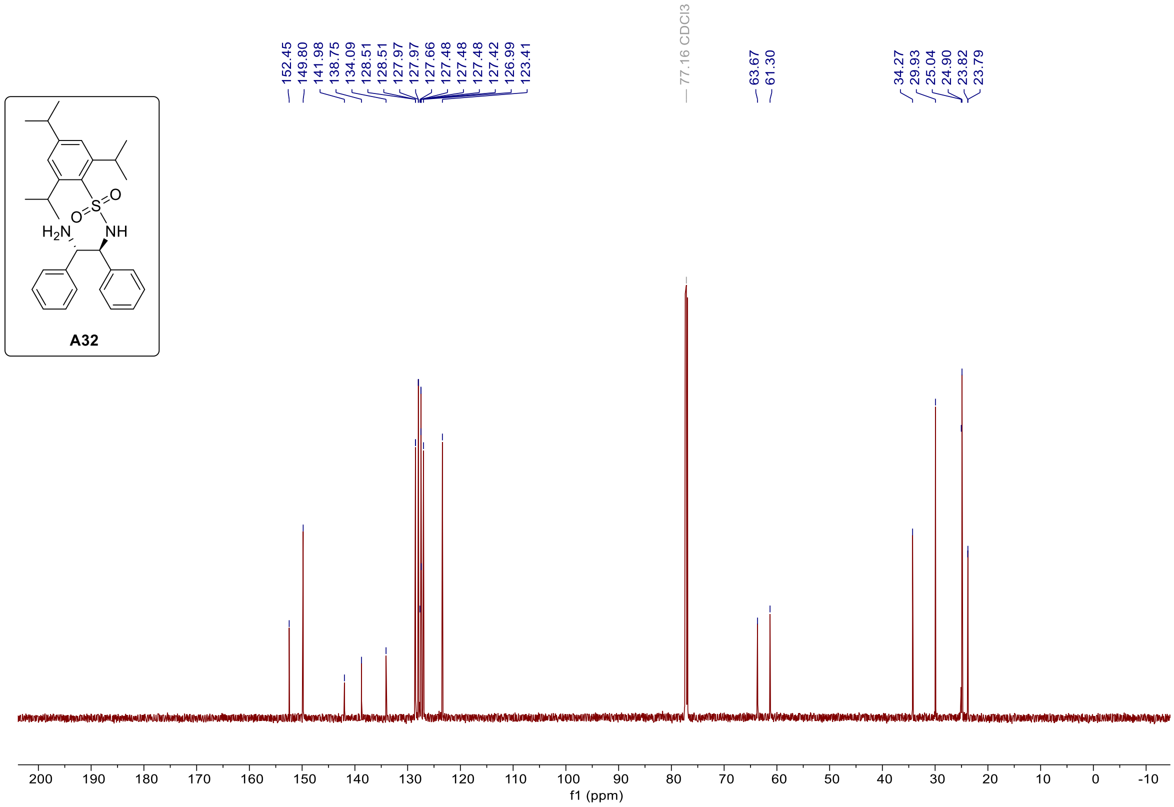

^{1}H NMR (600 MHz, $CDCl_3$) δ 7.87 (s, 2H), 7.78 (s, 1H), 7.16–7.06 (m, 10H), 4.55 (d, J = 5.5 Hz, 1H), 4.23 (d, J = 5.6 Hz, 1H).

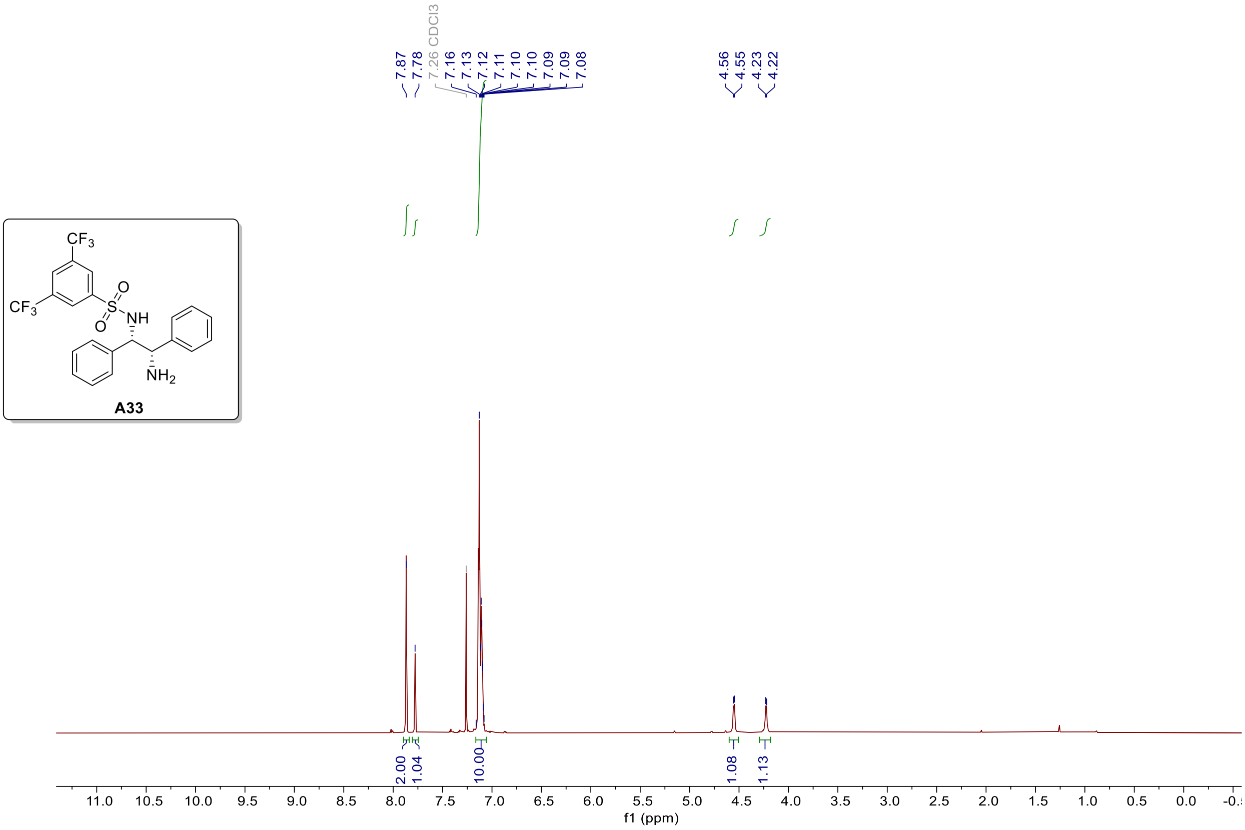

^{13}C NMR (151 MHz, $CDCl_3$) δ 143.28, 138.00, 132.63, 132.40, 132.17, 131.95, 128.70, 128.64, 128.11, 128.07, 127.24, 127.22, 127.12, 126.43, 125.59, 123.40, 121.59, 119.78, 63.53, 60.12.

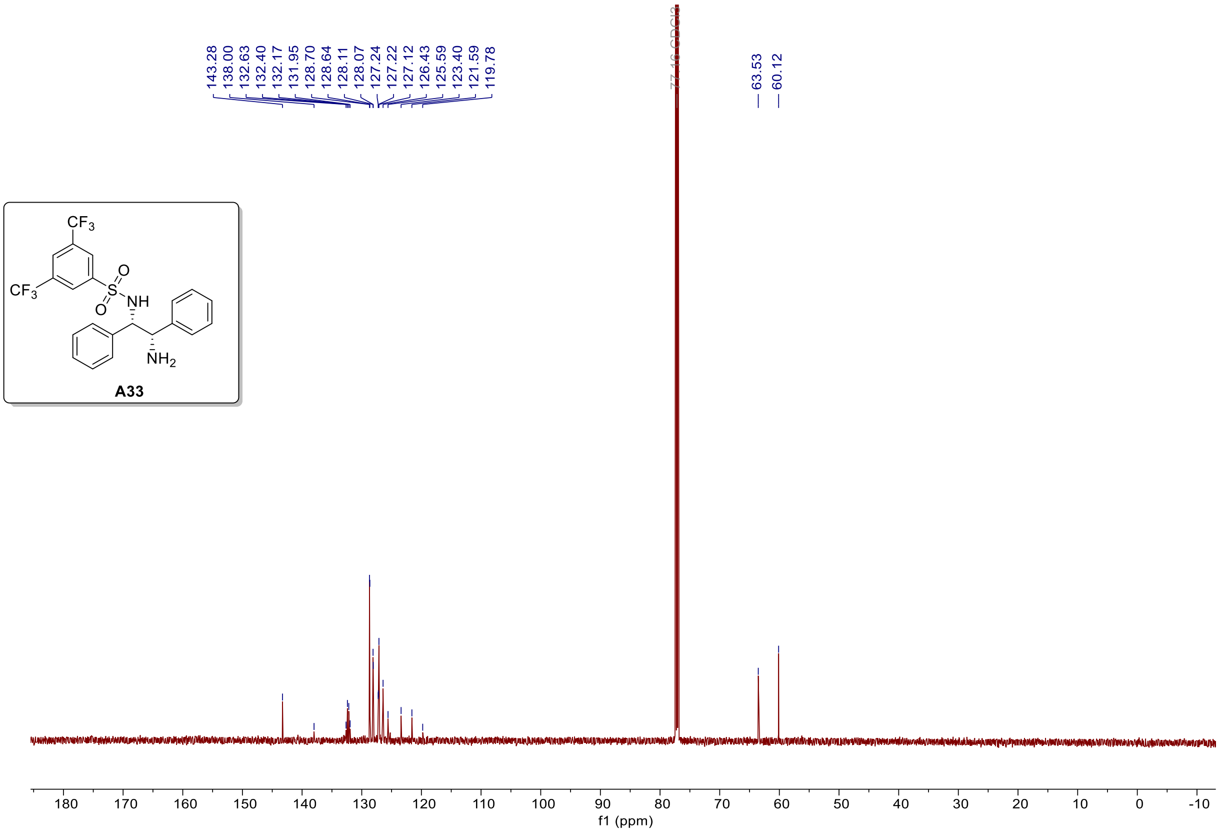

^{1}H NMR (600 MHz, $CDCl_3$) δ 7.97 (d, *J* = 1.9 Hz, 1H), 7.79 (d, *J* = 8.2 Hz, 1H), 7.71 (d, *J* = 8.7 Hz, 1H), 7.62 (d, *J* = 8.7 Hz, 1H), 7.60–7.55 (m, 1H), 7.54–7.48 (m, 1H), 7.44 (dd, *J* = 8.6, 1.9 Hz, 1H), 7.16–7.11 (m, 2H), 7.11–7.04 (m, 4H), 7.04–6.93 (m, 4H), 4.51 (d, *J* = 5.6 Hz, 1H), 4.20 (d, *J* = 5.6 Hz, 1H).

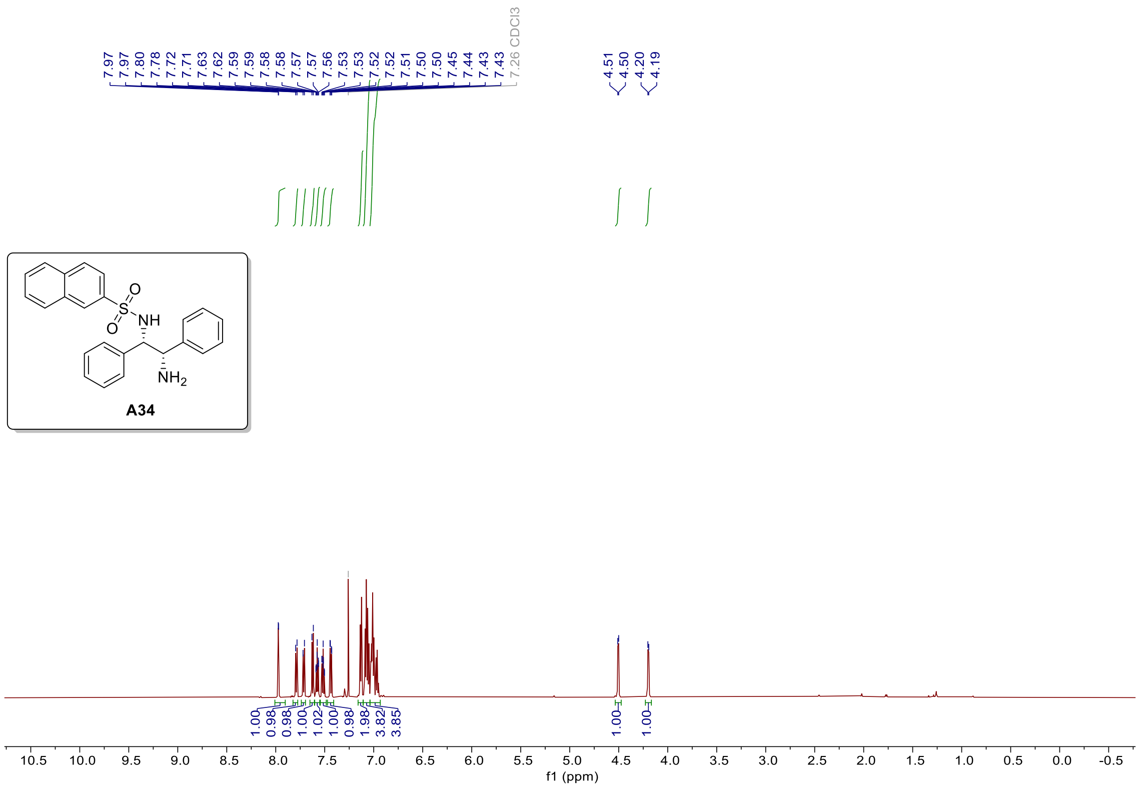

^{13}C NMR (151 MHz, $CDCl_3$) δ 140.98, 139.03, 137.09, 134.57, 132.02, 129.32, 128.96, 128.48, 128.44, 128.32, 128.29, 127.74, 127.65, 127.56, 127.12, 127.08, 126.59, 122.30, 63.40, 60.45.

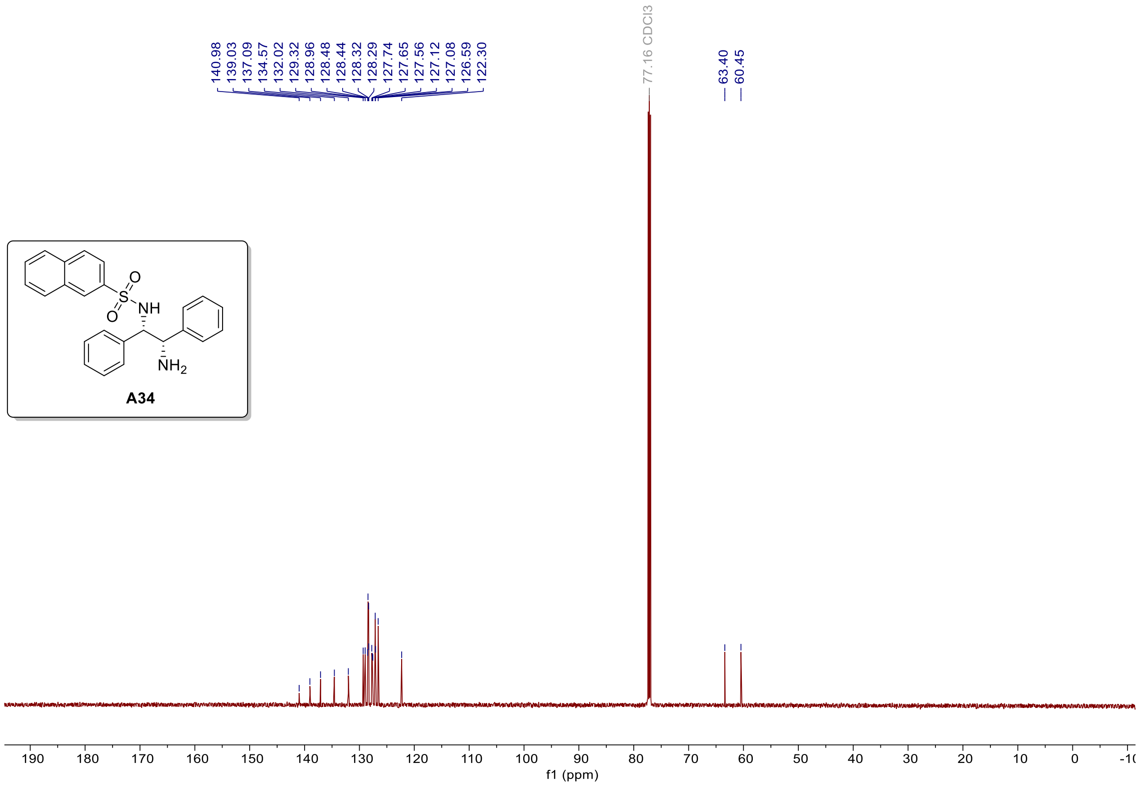

^{1}H NMR (600 MHz, $CDCl_3$) δ 9.83 (s, 1H), 8.12–7.85 (m, 4H), 7.70–7.55 (m, 1H), 7.47 (t, J = 7.7 Hz, 2H), 3.48 (dd, J = 17.8, 7.8 Hz, 1H), 3.26–3.04 (m, 1H), 3.06–2.99 (m, 1H), 1.83–1.77 (m, 1H), 1.54 (td, J = 15.0, 8.7, 6.8 Hz, 1H), 1.43–1.22 (m, 4H), 0.91 (t, J = 7.1 Hz, 2H).

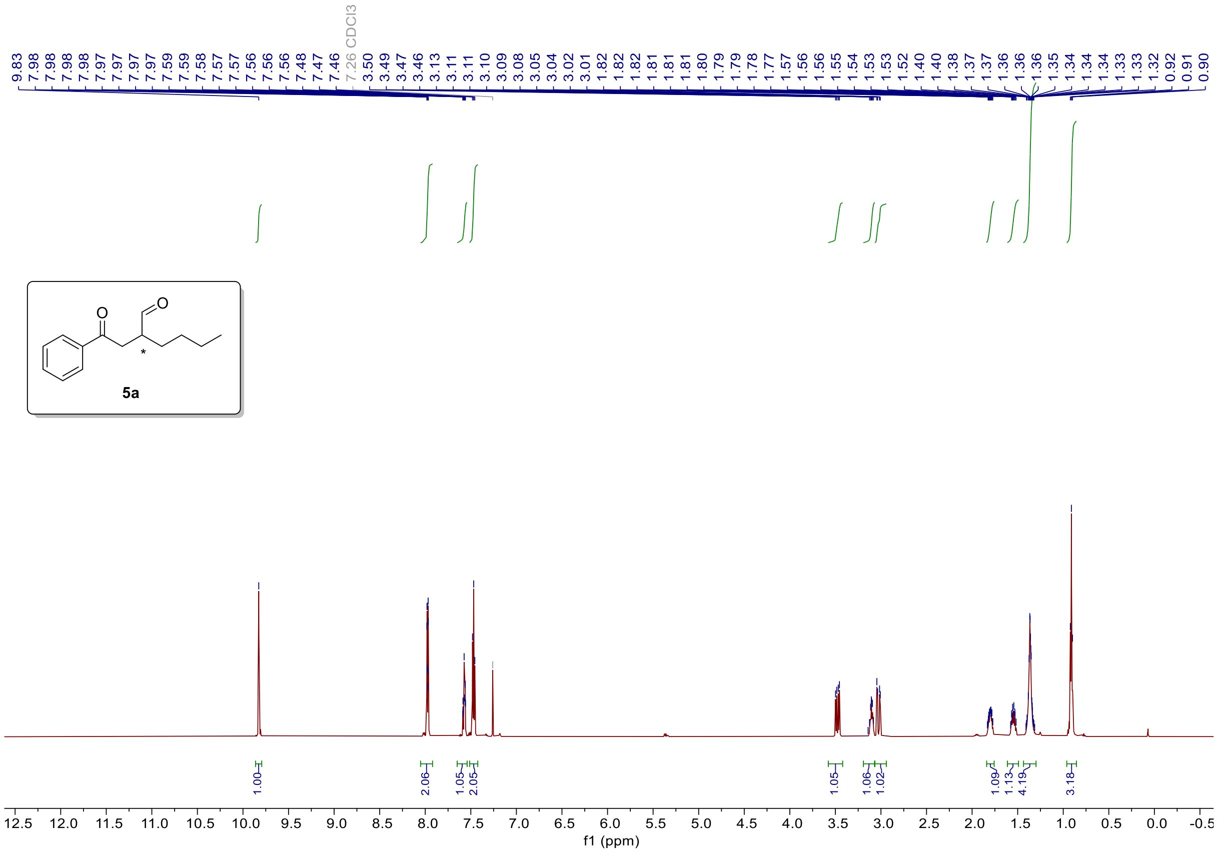

^{13}C NMR (151 MHz, $CDCl_3$) δ 203.81, 198.19, 136.72, 133.44, 128.78, 128.23, 46.85, 37.79, 29.37, 28.71, 22.90, 14.00.

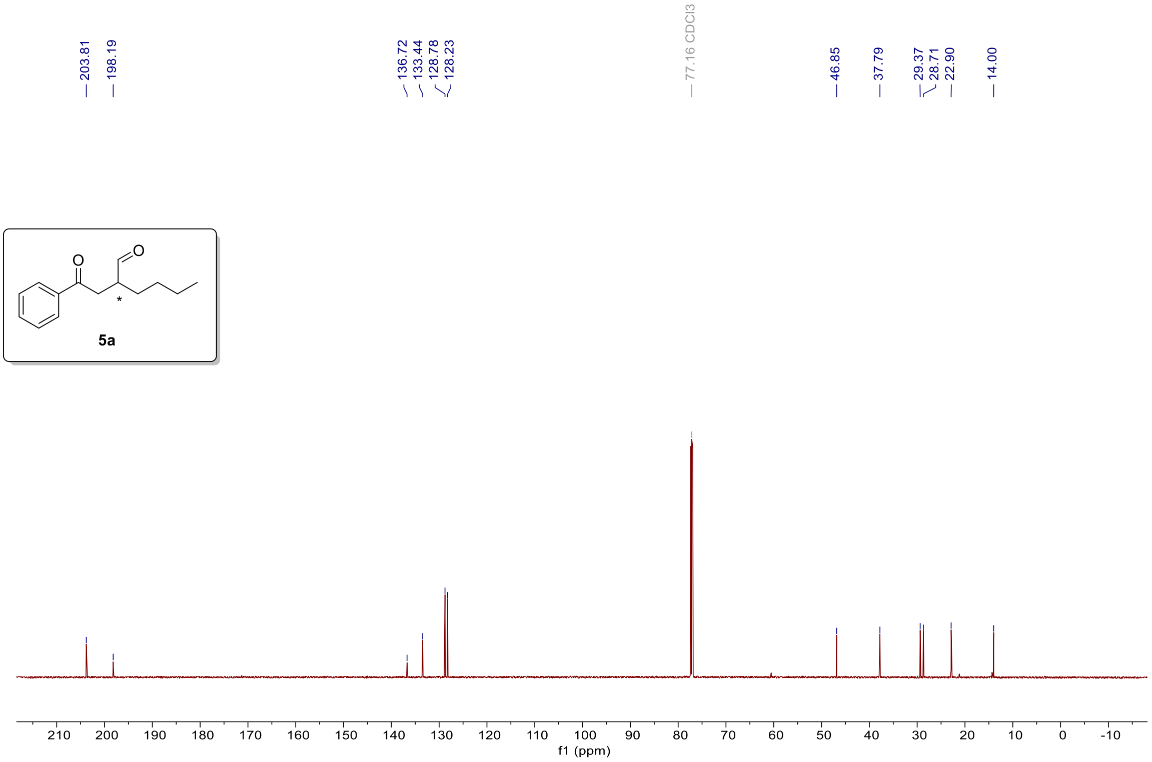

^{1}H NMR (600 MHz, $CDCl_3$) δ 9.83 (d, J = 1.0 Hz, 1H), 7.97 (dt, J = 8.5, 1.3 Hz, 2H), 7.66–7.54 (m, 1H), 7.51–7.38 (m, 2H), 3.49 (dd, J = 17.8, 7.7 Hz, 1H), 3.18–3.00 (m, 2H), 2.25 (td, J = 6.8, 2.6 Hz, 2H), 2.00–1.87 (m, 2H), 1.82–1.46 (m, 3H).

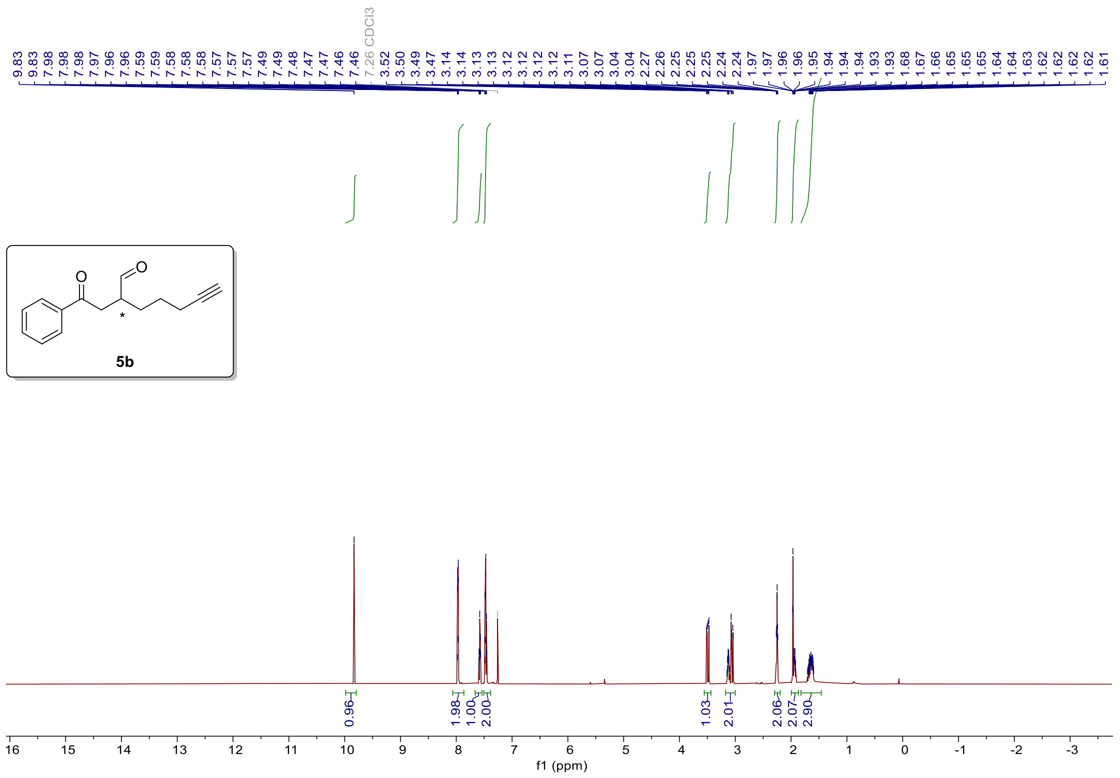

^{13}C NMR (151 MHz, $CDCl_3$) δ 203.28, 197.89, 136.58, 133.54, 128.81, 128.23, 83.61, 69.19, 46.39, 37.85, 27.98, 26.02, 18.59.

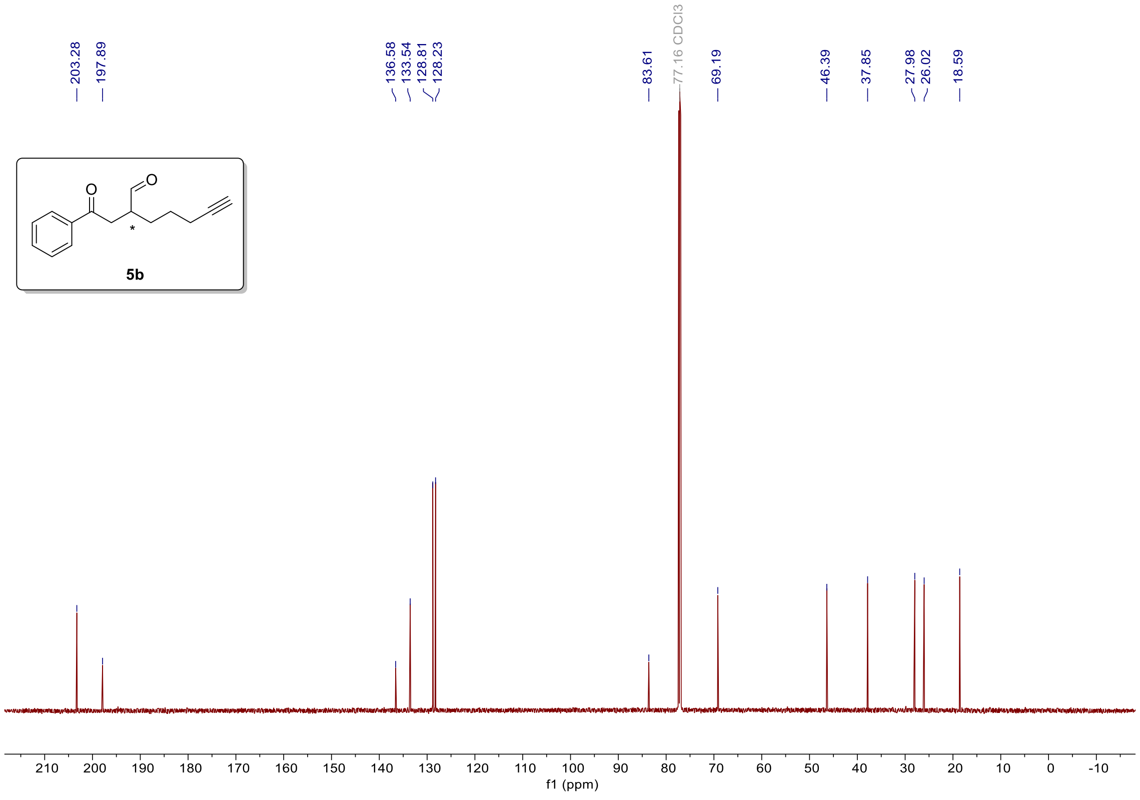

^{1}H NMR (600 MHz, $CDCl_3$) δ 9.82 (d, *J* = 1.0 Hz, 1H), 8.03–7.93 (m, 2H), 7.61–7.54 (m, 1H), 7.50–7.44 (m, 2H), 3.47 (dd, *J* = 17.7, 7.8 Hz, 1H), 3.18–3.08 (m, 1H), 3.03 (dd, *J* = 17.7, 4.8 Hz, 1H), 2.19 (td, *J* = 6.9, 2.7 Hz, 2H), 1.94 (t, *J* = 2.6 Hz, 1H), 1.86–1.77 (m, 1H), 1.60–1.50 (m, 3H), 1.48–1.37 (m, 4H).

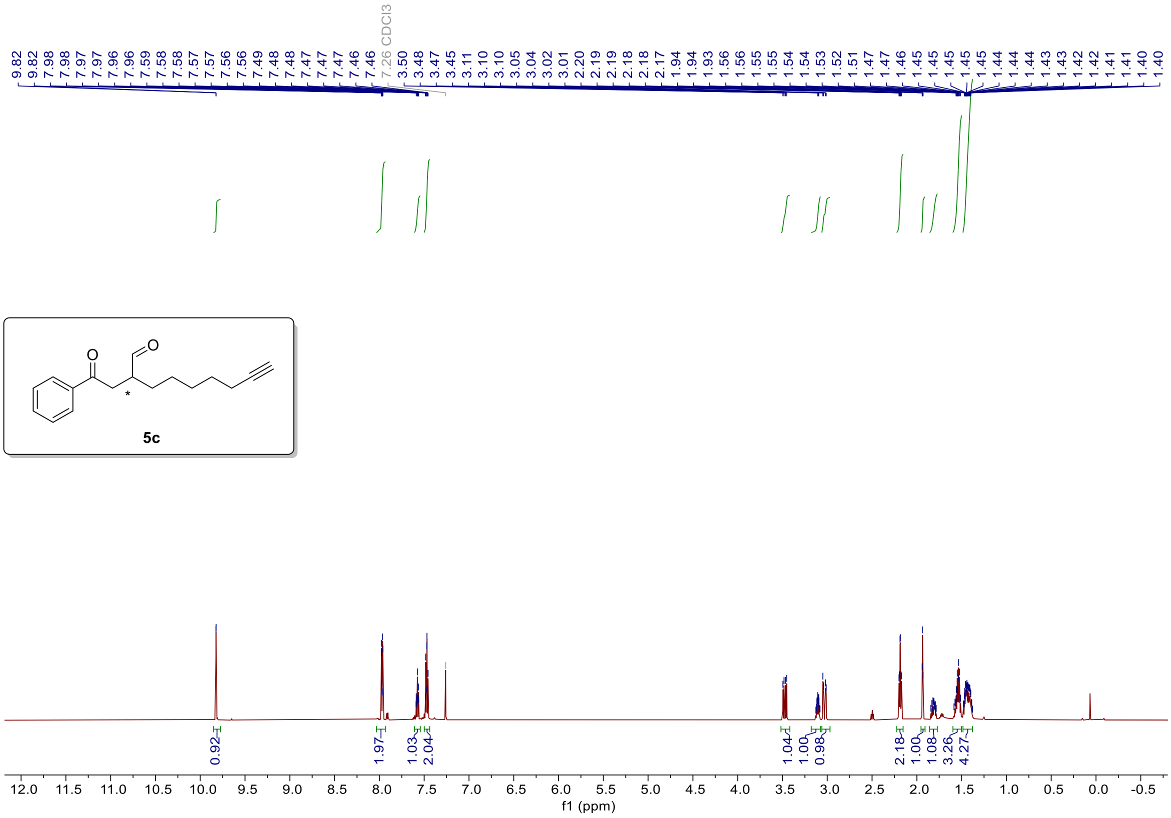

^{13}C NMR (151 MHz, $CDCl_3$) δ 203.64, 198.08, 136.66, 133.48, 128.80, 128.23, 84.48, 68.53, 46.80, 37.80, 28.87, 28.83, 28.26, 26.75, 18.43.

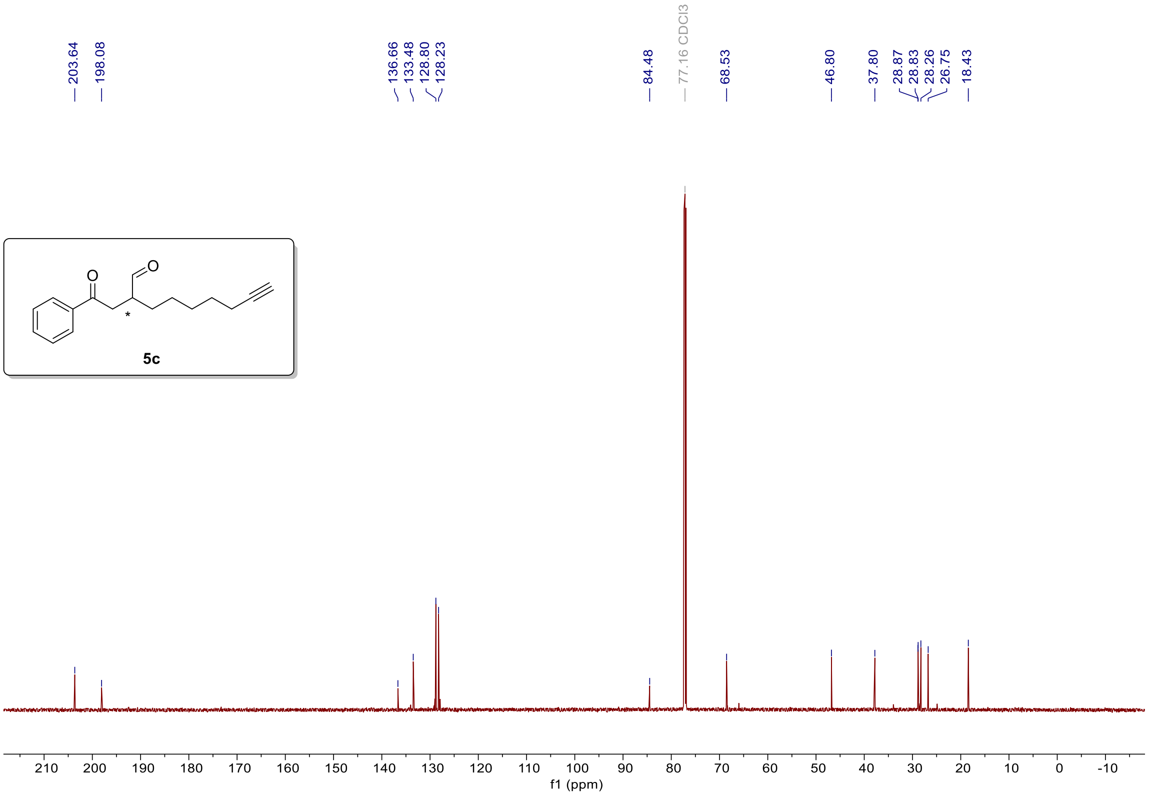

^{1}H NMR (600 MHz, $CDCl_3$) δ 9.83 (d, *J* = 1.1 Hz, 1H), 7.95 (d, *J* = 8.9 Hz, 2H), 6.94 (d, *J* = 8.9 Hz, 2H), 3.88 (s, 3H), 3.44 (dd, *J* = 17.6, 7.6 Hz, 1H), 3.15–3.05 (m, 1H), 3.02 (dd, *J* = 17.6, 4.9 Hz, 1H), 2.25 (td, *J* = 6.7, 2.6 Hz, 2H), 1.96 (t, *J* = 2.7 Hz, 1H), 1.71–1.58 (m, 4H).

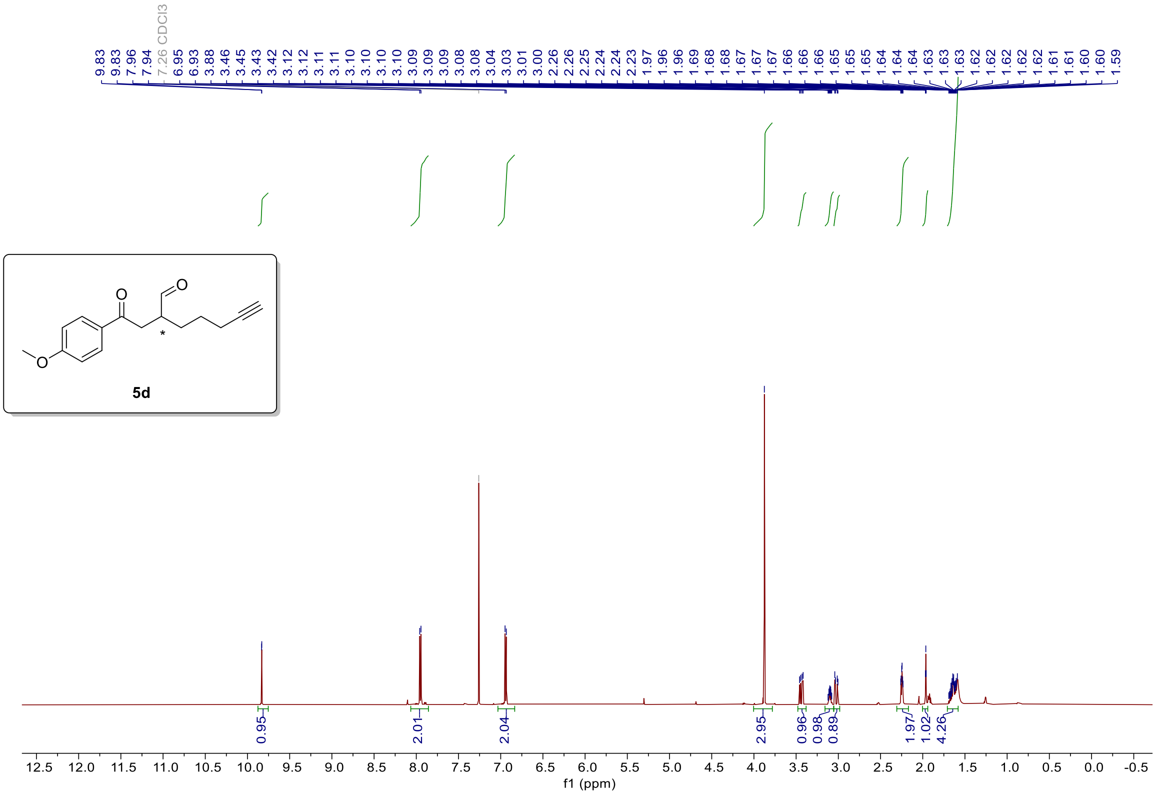

^{13}C NMR (151 MHz, $CDCl_3$) δ 203.50, 196.35, 163.87, 130.55, 129.69, 113.95, 83.69, 69.15, 55.66, 46.47, 37.60, 28.06, 26.06, 18.61.

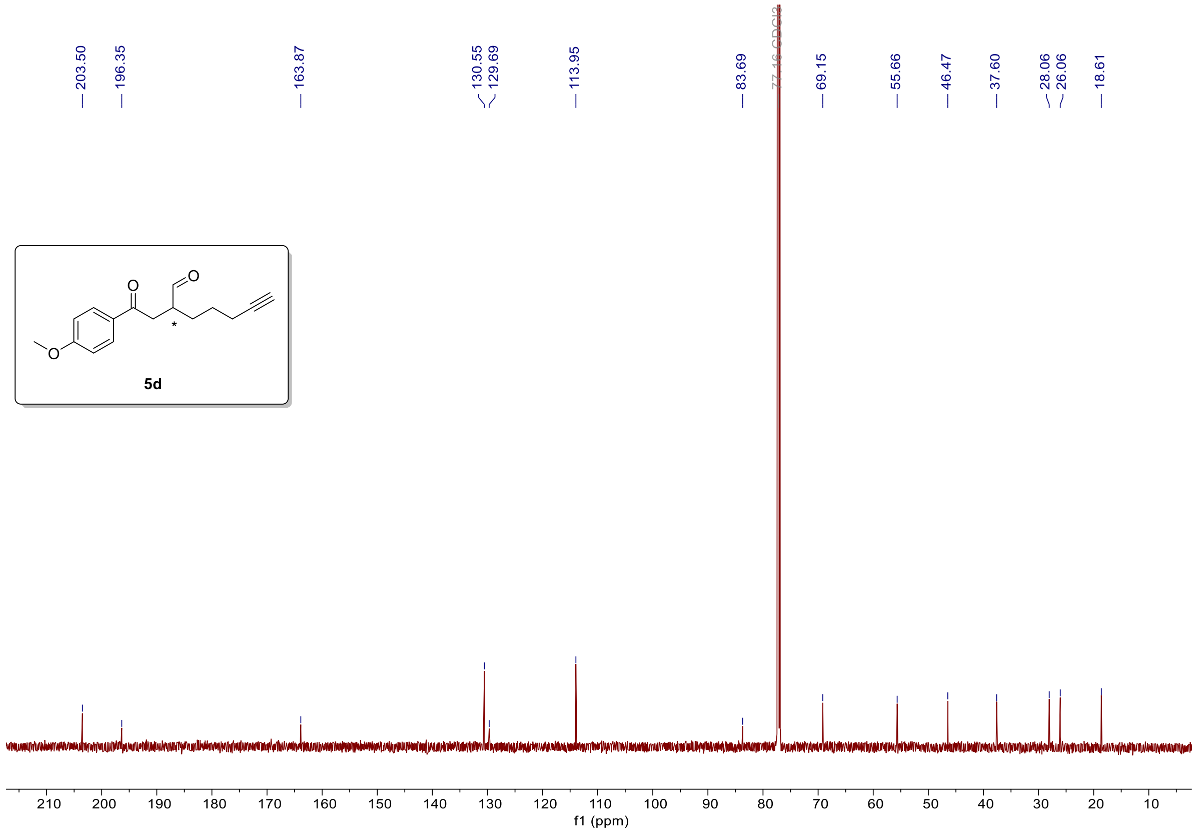

^{1}H NMR (600 MHz, $CDCl_3$) δ 9.82 (d, *J* = 1.1 Hz, 1H), 7.95 (d, *J* = 8.9 Hz, 2H), 6.94 (d, *J* = 8.8 Hz, 2H), 3.87 (s, 3H), 3.42 (dd, *J* = 17.5, 7.7 Hz, 1H), 3.17–3.04 (m, 1H), 3.00 (dd, *J* = 17.5, 5.0 Hz, 1H), 2.18 (td, *J* = 7.0, 2.7 Hz, 2H), 1.93 (t, *J* = 2.6 Hz, 1H), 1.85–1.75 (m, 1H), 1.59–1.33 (m, 7H).

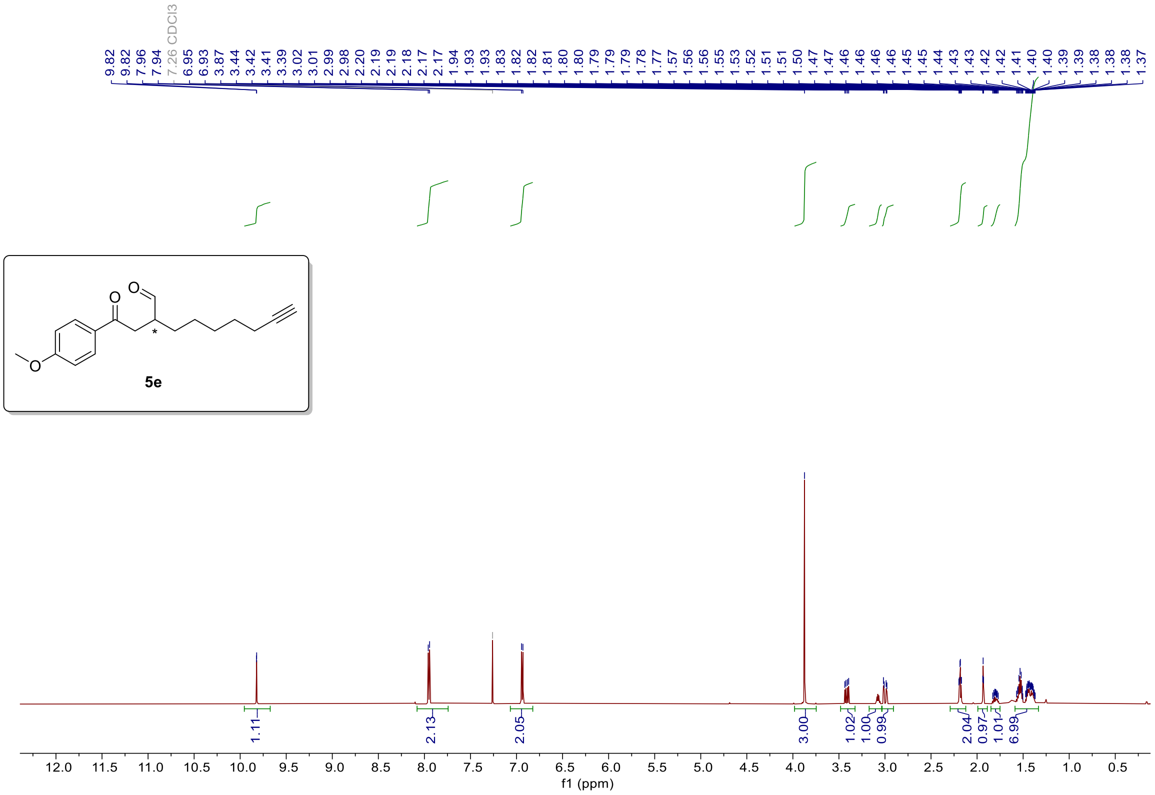

^{13}C NMR (151 MHz, $CDCl_3$) δ 203.87, 196.55, 163.81, 130.53, 129.76, 113.92, 84.51, 68.51, 55.64, 46.88, 37.54, 28.92, 28.85, 28.28, 26.76, 18.43.

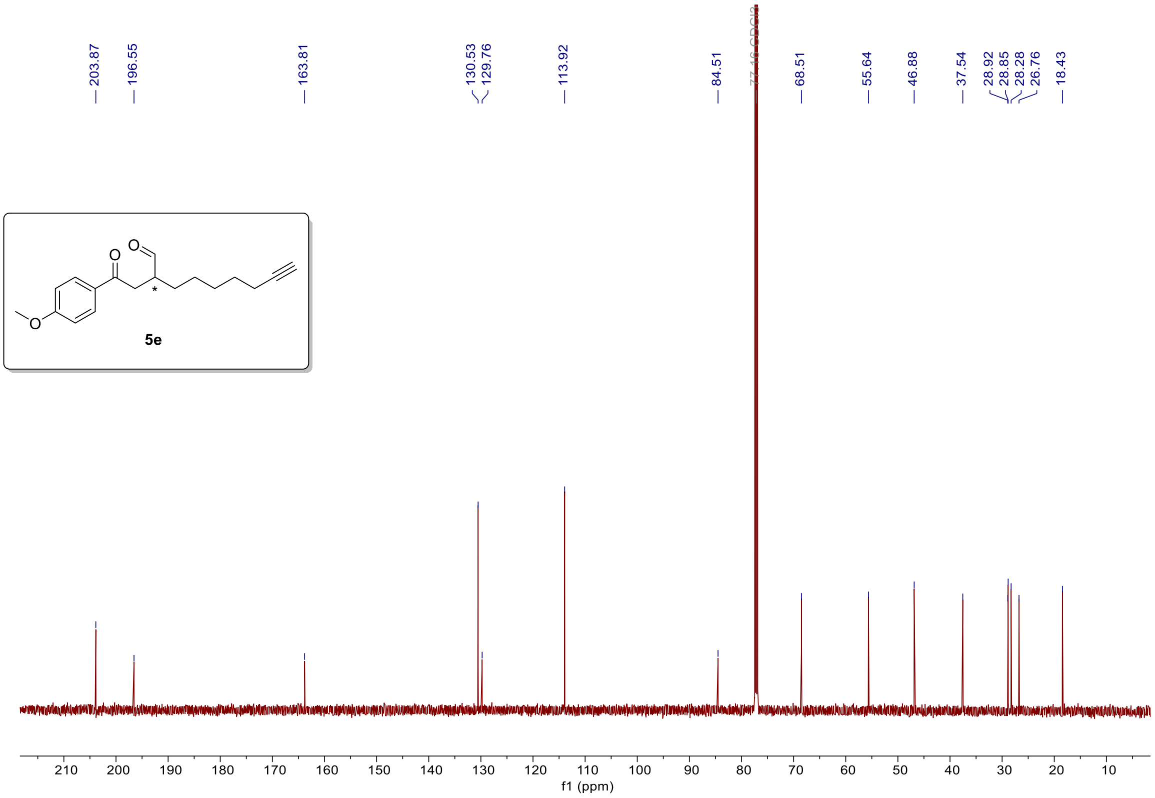

9. References

1. Cynthia A. Bunders, Justin J. Richards, Christian Melander, Identification of aryl 2-aminoimidazoles as biofilm inhibitorsin Gram-negative bacteria. *Bioorg. Med. Chem. Lett.* **20,** 3797-3800 (2010).
2. Guangliang Tu, Dongjie Wang, Chunchen Yuan, Jingyu Zhang, and Yingsheng Zhao, Palladium-Catalyzed Para-Selective Difluoromethylation of Arene Esters. *J. Org. Chem.* **85**, 16, 10740–10749 (2020).
3. Alarcon, K., Martz, A., Mony, L., Neyton, J., Paoletti, P., Goeldner, M., & Foucaud, B. Reactive derivatives for affinity labeling in the ifenprodil site of NMDA receptors. *Bioorg. Med. Chem. Lett.* **18**, 2765–2770 (2008).
4. Hans, R. H., Guantai, E. M., Lategan, C., Smith, P. J., Wan, B., Franzblau, S. G., Chibale, K. Synthesis, antimalarial and antitubercular activity of acetylenic chalcones. *Bioorg. Med. Chem. Lett.* **20**, 942–944 (2010).
5. S. Hashiguchi, A. Fujii, J. Takehara, T. Ikariya, R. Noyori, Asymmetric Transfer Hydrogenation of Aromatic Ketones Catalyzed by Chiral Ruthenium(II) Complexes. *J. Am. Chem. Soc*. **117**, 7562-7563 (1995).
6. Breder, A., Rode, K., Palomba, M., Ortgies, S., & Rieger, R. Aerobic Allylation of Alcohols with Non-Activated Alkenes Enabled by Light-Driven Selenium-π-Acid Catalysis. *Synthesis*. **50,** 3875-3885 (2018).
7. Thomas H. Graham, Benjamin D. Horning, David W. C. MacMillan, The preparation of (2R,5S)-2-*t*-butyl-3,5-dimethyllimidazolidin-4-one. *Org. Synth*. **88**, 42-54 (2011).
